# Supplementary material for: Reprograming of the ubiquitin ligase Ubr1 by intrinsically disordered Roq1 through cooperating multifunctional motifs
Source: EMBO J. 2025 Feb 7;44(6):1774–803. doi: 10.1038/s44318-025-00375-7 (PMC11914429; doi:10.1038/s44318-025-00375-7)
Supplement: Supplementary file 1 — Appendix [file 44318_2025_375_MOESM1_ESM.pdf]

Appendix for

Reprograming of the ubiquitin ligase Ubr1 by intrinsically disordered Roq1 through cooperating multifunctional motifs

TABLE OF CONTENTS

p2 - Appendix Table S1 - Oligonucleotides used in this study

p6 - Appendix Figure S1 - Isolation and purity of recombinant proteins

Appendix Table S1 - Oligonucleotides used in this study.

Oligonucleotide	Sequence
Pho8* fw	tttaagaaggagatatacatatgtctgcatcacacaagaagaag
Pho8* rev	ctcagtggtggtggtggtggtgtatttctgtagcatcaaaatctg
His$_6$-SUMO-Roq1(22-104)(R22A) fw	tggtcccgctggctcgcaccaccaatctgttc
His$_6$-SUMO-Roq1(22-104)(R22A) rev	gaacagattggtggtgcgagccagcgggacca
KGEQ replacement fw	tcacagagaacagattggtggtaggagcaaggggggaagaactgttcacgggtg
KGEQ replacement revPhe-L-GFP rev	cacccgtgaacagttcttccccccttgctcctaccaccaatctgttctctgtga
Phe-L-GFP rev	tcttccccccttgctgaaaccaccaatctgttctctgtgagcc
Phe-L-GFP fw	ggctcacagagaacagattggtggtttcagcaaggggggaaga
Cup9 fw	gttcctgactatgcgaattataactgcgaaatacaaaacagg
Cup9 rev	gtggtggtggtggtgattcatatcagggttggatagc
pCA528 Pho8* fw	ggctcacagagaacagattggtgggatgtctgcatcacacaagaagaagaatg
pCA528 Pho8* rev	ggagctcgaattcggatccggtcttcatatttctgtagcatcaaaatctgatgtgtg
pCA528 open fw	agaccggatccgaattcg
pCA528 open rev	cccaccaatctgttctctg
Pho8* MBP fw	atcagattttgatgctacagaaataatgaaaatcgaagaaggtaaactgg
Pho8* MBP rev	agctcgaattcggatccggtcttcaagtctgcgcgtctttcag
Pho8* MBP open fw	tgaagaccggatccgaattc
Pho8* MBP open rev	tatttctgtagcatcaaaatctgatg
Pho8* fw seq	ccaatacgttttggagtttgctg
Pho8* intern seq fw	aagtcaaggtggctttggg
MBP intern seq. rev	ttgtaacgtacggcatccc
MBP intern seq. fw	aaatcatgccgaacatccc
Pho8 fw	gttggctttcgatgaagcatttcaatacgttttggag
Pho8 rev	ctccaaaacgtattgaaatgcttcatcgaaagccaac
Mgt1 fw	ggctcacagagaacagattggtgggatgaaggaactgctttactatacattc
Mgt1 rev	cacccgaaccaccacccgaaccacccaatctactaaggcttaagctattttcc
pCA528 Mgt1 open rev	cccaccaatctgttctctgtgagc
pCA528 Mgt1 open fw	ggtggttcgggtggtggt
Ubr1 -102 bp	tcgtcattgttctcgttcc
Ubr1 0 bp	atgtggtctcatccgcagtttg
Ubr1 +928 bp	agacgagcccctctaatagc
Ubr1 +1880 bp	aggtggtgtattgatctgg
Ubr1 + 2863 bp	agctactaagatcagttcc
Ubr1 +3819 bp	gaatttatgcccatgtgggatgg
Ubr1 +4844 bp	agattctgaaaatgaaacgc
Roq1(22-60)-HA fw	tacccatacgatgttcctgactatgcgtgaactaatttaggcatacaggaaaacacaagc
Roq1(22-60)-HA rev	cagctctacaaagtagtatatcacgcc
pho-HA-tag fw	tacccatacgatgttcctgactatgcgtgaggatccgaattcgagct
FLAG-Ubr1 His removal fw	taactcgagatcatgtaattagttatgtcacgc
FLAG-Ubr1 His removal rev	ccaaatctctcgctcatcagagtc
Ubr1 (I687D) rev	ggaggtggtatattctaaataggagtcgaaattttgatcttcgtgaagaaca
Ubr1 (I687D) fw	tgttcttcacgaagatcaaaatttcgactcctatttagaatataccacctcc
Ubr1 (Y823D) rev	taattcgggattgtttttataatccgatgcttgatgtaacacagaca
Ubr1 (Y823D) fw	tgtctgtgttacatcaagcatcggattataaaaacaatcccgaatta
MBP intern seq. rev	ttgtaacgtacggcatccc
HA-term fw	tacccatacgatgttcctgactatgcgtgaggatccgaattcgagct
Roq1-HA rev	cgcatagtcaggaacatcgtatgggtatgaacaacggcgagagtgaa
His-SUMO-Roq1 fw	tgaggatccgaattcgagct
His-SUMO-Roq1 rev	tgaacaacggcgagagtgaa
Open FLAG-Ubr1 Chk1 rev	gtcgactagaggatccccg

Open FLAG-Ubr1 Chk1 fw	taactcgagatcatgtaattagttatgtcacgc
Chk1-ALFA-FLAG fw	atgagtctctcgcaggtgtcacctttacccc
Chk1-ALFA-FLAG rev	cttgtcatcgtcgtccttgtagtcacccgaacccggctcggtcaatcttctcc
Roq1_I54K fw	gctctacaaagtagtatttcacgccgccag
Roq1_I54K rev	acacagcctggcgtgaaatactac
FLAG fw_new	gattacaaggacgacgatgacaag
ADH rev_new	tctagagcggccagcttggagt
Ubr1 fw	tccgttgctgatgatgatttaggat
Roq1-22 fw	aggagccagcgggacca
Ubi-Roq1-22 rev	ctggtcccgctggctcctaccacctcttagccttagca
GPD-Ubi fw	accagaacttagtttcgacggattctagaactagtatgc
Roq1_V58E fw	gcctaaattagtcagctcttcaaagtagtatatcacgcca
Roq1_V58E rev	tggcgtgatatactactttgaagagctgactaatttaggc
Roq1_Y55H fw	gctctacaaagtagtgtatcacgccaggctgtgtag
Roq1_Y55H rev	ctacacagcctggcgtgatacactactttgtagagc
Roq1_Q50K fw	cacgccaggctttgtaggctggttattgatccc
Roq1_Q50K rev	gggatcaataaccagcctacaaagcctggcgtg
Roq1_G40C fw	tgatcccgaaatcgcacatgggagactcctc
Roq1_G40C rev	gaggagtctcccatgtgcgatttcgggatca
Roq1_Y56N fw	agtcagctctacaaagttgtatatcacgccaggct
Roq1_Y56N rev	agcctggcgtgatatacaactttgtagagctgact
Roq1_E67K fw	gttattattactgcttgtgtttttctgtatgcctaaattagtcagct
Roq1_E67K rev	agctgactaatttaggcatacagaaaaacacaagcagtaataataac
Roq1_M39A_fw	ggtagaggagtctcccgcgggcgatttcgggatc
Roq1_M39A_rev	gatcccgaaatcgcccgcgggagactcctctacc
Roq1_Y55A_fw	cctacacagcctggcgtgatagcctactttgtagagctgac
Roq1_Y55A_rev	gtcagctctacaaagtaggctatcacgccaggctgtgtagg
Roq1_N62A_fw	gatatactactttgtagagctgactgctttaggcatacaggaaaacacaagc
Roq1_N62A_rev	gcttgtgttttcctgtatgcctaaagcagtcagctctacaaagtagtatatc
Roq1_I65A_fw	ctttgtagagctgactaatttaggcgcacaggaaaacacaagcagtaataat
Roq1_I65A_rev	attattactgcttgtgttttcctgtgcgcctaaattagtcagctctacaaag
Roq1_E67A_fw	gctgactaatttaggcatacaggcaaacacaagcagtaataataaca
Roq1_E67A_rev	tgttattattactgcttgtgtttgcctgtatgcctaaattagtcagc
Roq1_Y55N fw	ctacacagcctggcgtgataaactactttgtagagc
Roq1_Y55N rev	gctctacaaagtagtttatcacgccaggctgtgtag
Roq1_Y55F_fw	acagcctggcgtgatattctactttgtagagctg
Roq1_Y55F_rev	cagctctacaaagtagaatatcacgccaggctgt
Roq1_E36A_fw	ttccttggtagaggcgtctcccatgggcg
Roq1_E36A_rev	cgcccatgggagacgcctctaccaaggaa
Roq1_N45A_fw	gctgtgtaggctggttagcgatcccgaaatcgccca
Roq1_N45A_rev	tgggcgatttcgggatcgctaaccagcctacacagc
Roq1_Q50A_fw	tcacgccaggcgctgtaggctggttattgatccc
Roq1_Q50A_rev	gggatcaataaccagcctacagcgcctggcgtga
Roq1_I54A_fw	cagctctacaaagtagtatgccacgccaggctgtgtaggc
Roq1_I54A_rev	gcctacacagcctggcgtggcatactactttgtagagctg
Roq1_I54E_fw	gtcagctctacaaagtagtactccacgccaggctgtgtaggct
Roq1_I54E_rev	agcctacacagcctggcgtggagtactactttgtagagctgac
Roq1_Y56D_fw	agtcagctctacaaagtcgtatatcacgccaggct
Roq1_Y56D_rev	agcctggcgtgatatacgactttgtagagctgact

Roq1_L60E_fw	tttcctgtatgcctaaattagtctcctctacaaagtagtatatcacgc
Roq1_L60E_rev	gcgtgatatactactttgtagaggagactaatttaggcatacaggaaa
Roq1_E67A_fw_new	agttattattactgcttgtgtttgcctgtatgcctaaattagtcagc
Roq1_E67A_rev_new	gctgactaatttaggcatacaggcaaacacaagcagtaataataact
Roq1_Y55D_fw	ctacacagcctggcgtgatagactactttgtagagc
Roq1_Y55D_rev	gctctacaaagtagtctatcacgccaggctgtgtag
Roq1_V53E_fw	ctacaaagtagtatatctcgccaggctgtgtagg
Roq1_V53E_rev	cctacacagcctggcgagatatactactttgtag
Roq1_Y56F_fw	tagtcagctctacaaagaagtatatcacgccaggc
Roq1_Y56F_rev	gcctggcgtgatatacttctttgtagagctgacta
Roq1_Y56A_fw	aattagtcagctctacaaaggcgtatatcacgccaggctgtg
Roq1_Y56A_rev	cacagcctggcgtgatatacgcctttgtagagctgactaatt
Roq1_F57D_fw	cctaaattagtcagctctacatcgtagtatatcacgccaggctg
Roq1_F57D_rev	cagcctggcgtgatatactacgatgtagagctgactaatttagg
Roq1_F57A_fw	cctaaattagtcagctctacagcgtagtatatcacgccaggctg
Roq1_F57A_rev	cagcctggcgtgatatactacgctgtagagctgactaatttagg
Roq1_V58A_fw	gcctaaattagtcagctctgcaaagtagtatatcacgcca
Roq1_V58A_rev	tggcgtgatatactactttgcagagctgactaatttaggc
Roq1 up res74_fw	ggaaaacacaagcagtaataataac
Roq1 down R22_rev	caaggaatccactctagtctgg
Roq1(22-104) R22A fw	ggctaagaggtggtgcgagccagcgggacc
Roq1(22-104) R22A rev	ggtcccgctggctcgcaccacctcttagcc
Roq1-LVEE_fw	ccagactagagtggattcctctcccacacagcctggc
Roq1-LVEE_rev	caggctgtgtgggagaggaatccactctagtctggtccc
Roq1-VDSLVEE_fw	cagcgggaccagactagatctcccacacagcctggc
Roq1-VDSLVEE_rev	ccaggctgtgtgggagatctagtctggtcccgctggc
Roq1-QTRVDSLVEE_fw	gtaggagccagcgggactctcccacacagcctggc
Roq1-QTRVDSLVEE_rev	caggctgtgtgggagagtcccgctggctcctacc
Roq1(22-60)HA 58E fw	cgtatgggtacagctcttcaaagtagtatatcacgc
Roq1(22-60)HA 58E rev	gcgtgatatactactttgaagagctgtacccatacg
Roq1(22-60)4A fw	tacccatacgatgttcctgactatgcgtgaactaatttaggcatacagg
Roq1(22-60)4A rev	tcacgcatagtcaggaacatcgtatgggtacagctctgcagctgcagc
Roq1 GGS linker fw	tctggtggctccggtcctggcgtgatatactactttgtagagctg
Roq1 GGS linker rev	tccgcctgaacctccgctcctaccacctcttagccttagcac
Roq1 GSP linker fw	cctgggggatccggtcctggcgtgatatactactttgtagagctg
Roq1 GSP linker rev	agaacctggtgatccgctcctaccacctcttagccttagcac
Roq1 pCA GGS_rev	tccgcctgaacctccgctcctaccaccaatctgttctctgtgag
Roq1 pCA GSP_rev	agaacctggtgatccgctcctaccaccaatctgttctctgtgag
pCAminimal Roq1_fw	agatctcccacacagcctggcgtgatatactactttgtagagctg
pCAminimal Roq1_rev	agtctggtcccgctggctcctaccaccaatctgttctctgtgag
Roq1_I54K fw	gctctacaaagtagtatttcacgccgccag
Roq1_I54K rev	acacagcctggcgtgaaatactac
gBlockALFA_for	ctgactaatttaggcatacaggaaaacacaag
gBlockALFA_rev	gccgtatcgactgccgt
GS10 27 31 gBlock for	tcaacaatatacaggacacacaaaaaaagcaac
GS10 27 gBlock rev	tatgcctaaattagtcagctctacaaagtagtatatcac
GS31 gBlockrev	tgtgttttcctgtatgcctaaattagtcagc
GS27 rev	ccagatccgcctgaacctccaggaccggagccaccagatcc
GS27 fw	tggctccggtccaggaggttcaggcggatctggtcctggcgtgatatactac
GS31 rev	cctgaacctcctggaccggagccaccagatccgcctgaacctccaggaccggagcc
GS31 fw	cggatctggtggcagcccaggttactactttgtagagctgactaatttaggc

Roq1 (19aa) fw	actaatttaggcatacaggaaaacacaagc
Roq1 (19aa) rev	cagctctacaaagtagtatatcacgc
P2S-Roq1N74 short	gagctgactaatttaggcatacaggaaaacacaagcagtaataataactagggataacagggtaatccgcgcgttggccgattcat
P1-Roq1-ALFA-T61	aatcttgagttattattactgcttgtgttttcctgtatgcctaaattagtttcgtacgctgcaggtcgac
X-tFT insert fw	cggattctagaactagtggatccatgcagattttcgtcaagactttgac
X-tFT insert rev	ccagcaccagcacctgctccgtcgagcttgtacagctcgtccatg
GPD seq	ccagttccctgaaattattccc
sfGFP rev seq	cgtccagttctaccaaaatgg
pRSS237 open fw	ctcgacggagcaggtg
pRSS237 open rev	ggatccactagttctagaatccg
GS27_31 genomic fw	catcttgtgctaaggctaagaggtggtaggagcggaggttcag
GS27_31 genomic rev	gcatagtcaggaacatcgtatgggtagttattattactgcttgtgttttcc
GS27_31 open fw	tacccatacgatgttcctgac
GS27_31 open rev	accacctcttagccttagc
gBlockALFA_for	ctgactaatttaggcatacaggaaaacacaag
P2S-Roq1L21	aaacttcaacaatatacaggacacacaaaaaaagcaacagtagtatactctagggataacagggtaatccgcgcgttggccgattcat
P2S-Roq1G52	agtctcccatgggcgatttcgggatcaataaccagcctacacagcctggctagggataacagggtaatccgcgcgttggccgattcat
P2S-Roq1N74	tagagctgactaatttaggcatacaggaaaacacaagcagtaataataactagggataacagggtaatccgcgcgttggccgattcat
P1-Roq1P51	tcctgtatgcctaaattagtcagctctacaaagtagtatatcacgccaggttcgtacgctgcaggtcgac
P1-Roq1T61	ttgttgttgttattattactgcttgtgttttcctgtatgcctaaattagt ttcgtacgctgcaggtcgac
P1-Roq1N75 neu	ccgtggccgtatcgactgccgttttcatcgtcaccatgattattgttgttttcgtacgctgcaggtcgac
Roq1-335bp	atcatcgaggacgttgagtg
P2S-Roq1L21	aaacttcaacaatatacaggacacacaaaaaaagcaacagtagtatactctagggataacagggtaatccgcgcgttggccgattcat
P2S-Roq1G52	agtctcccatgggcgatttcgggatcaataaccagcctacacagcctggctagggataacagggtaatccgcgcgttggccgattcat
P2S-Roq1N74	tagagctgactaatttaggcatacaggaaaacacaagcagtaataataactagggataacagggtaatccgcgcgttggccgattcat
P1-Roq1P51	tcctgtatgcctaaattagtcagctctacaaagtagtatatcacgccaggttcgtacgctgcaggtcgac

Appendix Figure S1

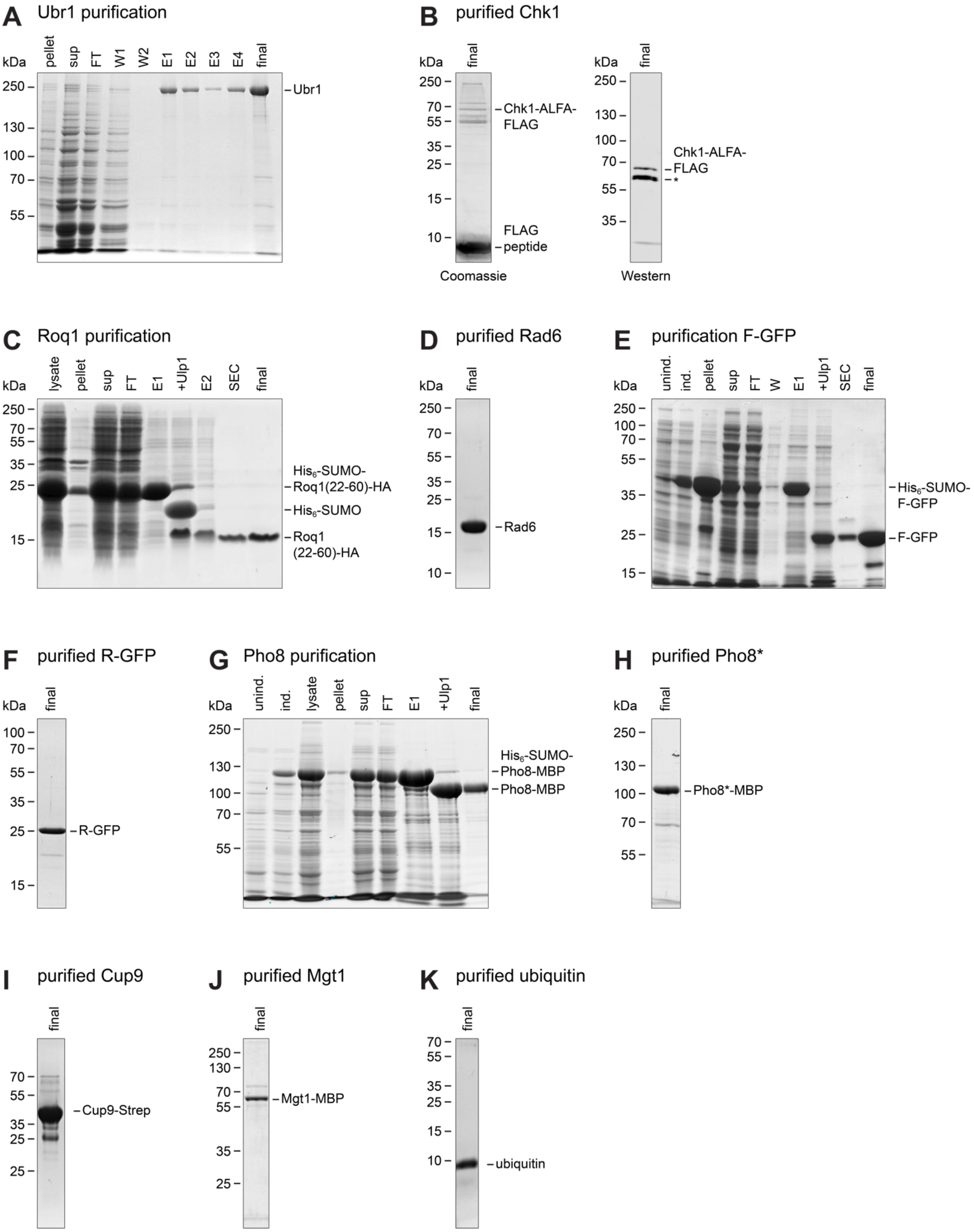

Appendix Figure S1. Isolation and purity of recombinant proteins.

A Coomassie-stained SDS-PAGE gel of FLAG-Ubr1 purification. Shown are the pellet (pellet) and supernatant (sup) of the centrifuged yeast lysate, the flow-through after incubation of the supernatant with anti-FLAG beads (FT), the washes of the anti-FLAG beads (W1, W2), the protein-containing fractions resulting from the elution with FLAG peptide (E1-E4) and the purified FLAG-Ubr1 (final).

B Coomassie-stained SDS-PAGE gel (left) and western blot (right) of purified Chk1-ALFA-FLAG. The purification of Chk1 followed the same strategy as that of Ubr1. The band of FLAG peptide in the Coomassie-stained gel stems from the elution from the anti-FLAG beads. The asterisk (*) denotes a degradation product that arose during expression or purification.

C Coomassie-stained SDS-PAGE gel of Roq1 purification, with Roq1(22-60)-HA as an example. Shown are the lysate of bacteria expressing His_6-SUMO-Roq1(22-60)-HA (lysate), the pellet (pellet) and supernatant (sup) of the centrifuged lysate, the flow-through (FT) and eluate (E1) of the immobilized metal affinity chromatography with nickel beads, the eluate after incubation with Ulp1 protease to cleave off His_6-SUMO (+Ulp1), the eluate after the second immobilized metal affinity chromatography with nickel beads (E2), the eluate of the size exclusion chromatography (SEC) and the purified Roq1(22-60)-HA (final).

D Coomassie-stained SDS-PAGE gel of purified Rad6. The purification of Rad6 followed the same strategy as that of Roq1.

E Coomassie-stained SDS-PAGE gel of F-GFP purification. Shown are the lysate of bacteria before and after induction of His_6-SUMO-F-GFP expression (unind. and ind.), the pellet (pellet) and supernatant (sup) of the centrifuged lysate, the flow-through (FT), wash (W) and eluate (E1) of the immobilized metal affinity chromatography with nickel beads, the eluate after incubation with Ulp1 protease to cleave off His_6-SUMO (+Ulp1), the eluate of the size exclusion chromatography (SEC) and the purified F-GFP (final).

F Coomassie-stained SDS-PAGE gel of purified R-GFP. The purification of R-GFP followed the same strategy as that of F-GFP.

G Coomassie-stained SDS-PAGE gel of Pho8-MBP purification. Shown are the lysed bacteria before induction (unind.) and after induction of His_6-SUMO-Pho8-MBP expression (ind.), the cell lysate used for purification (lysate), the pellet (pellet) and supernatant (sup) of the centrifuged lysate, the flow-through (FT) and eluate from the immobilized metal affinity chromatography with nickel beads (E1), the eluate after incubation with Ulp1 protease to cleave off His_6-SUMO (+Ulp1) and the eluate of the size exclusion chromatography (final).

H Coomassie-stained SDS-PAGE gel of purified Pho8*-MBP. The purification Pho8* followed the same strategy as that of Pho8.

I Coomassie-stained SDS-PAGE gel of purified Cup9-Strep. The purification of Cup9 followed the same strategy as that of Roq1.

J Coomassie-stained SDS-PAGE gel of purified Mgt1-MBP. The purification of Mgt1 followed the same strategy as that of Roq1.

K Coomassie-stained SDS-PAGE gel of purified ubiquitin.
