# Supplementary figures and images for: Reprograming of the ubiquitin ligase Ubr1 by intrinsically disordered Roq1 through cooperating multifunctional motifs
Source: EMBO J. 2025 Feb 7;44(6):1774–803. doi: 10.1038/s44318-025-00375-7 (PMC11914429; doi:10.1038/s44318-025-00375-7)

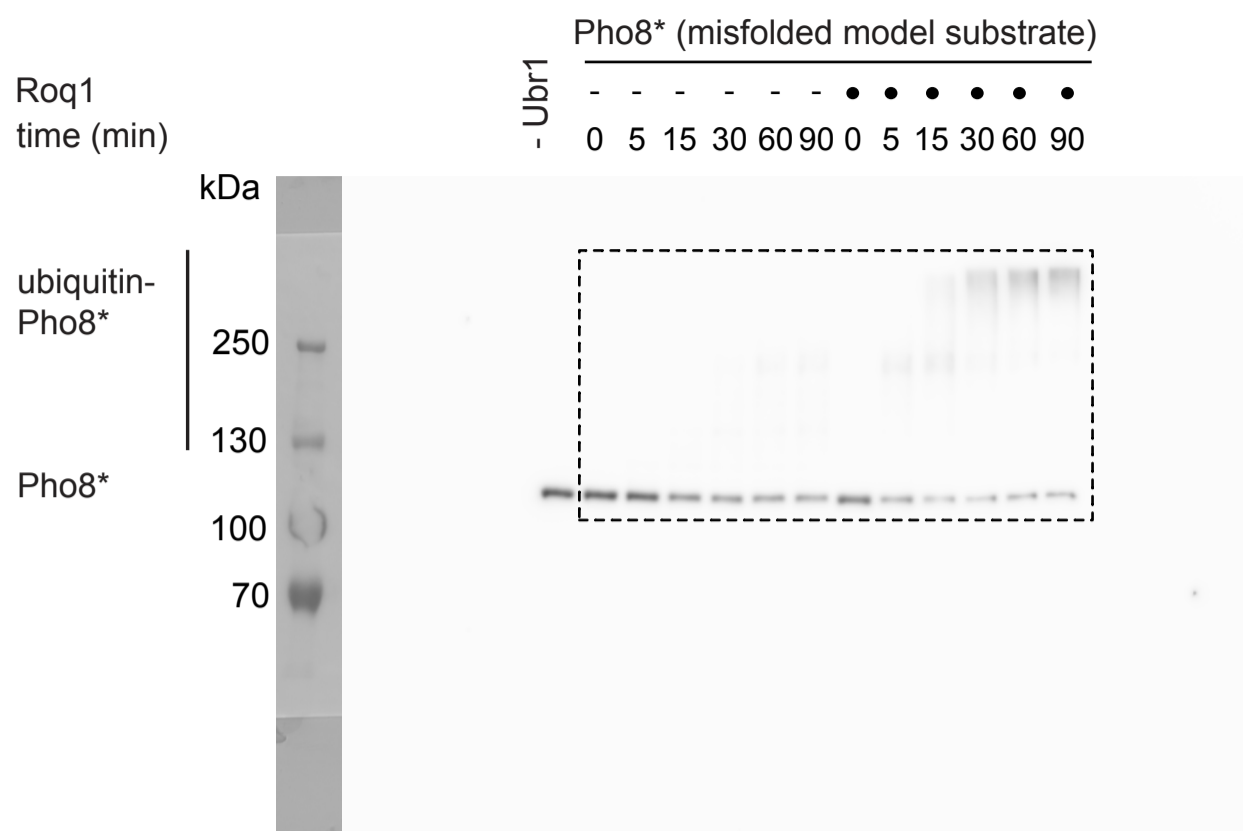

The boxed area was used for the final figure.

Supplement: Supplementary file 3 — Source data Fig. 1 [file 44318_2025_375_MOESM3_ESM.zip › Figure 1/Figure 1B.pdf]

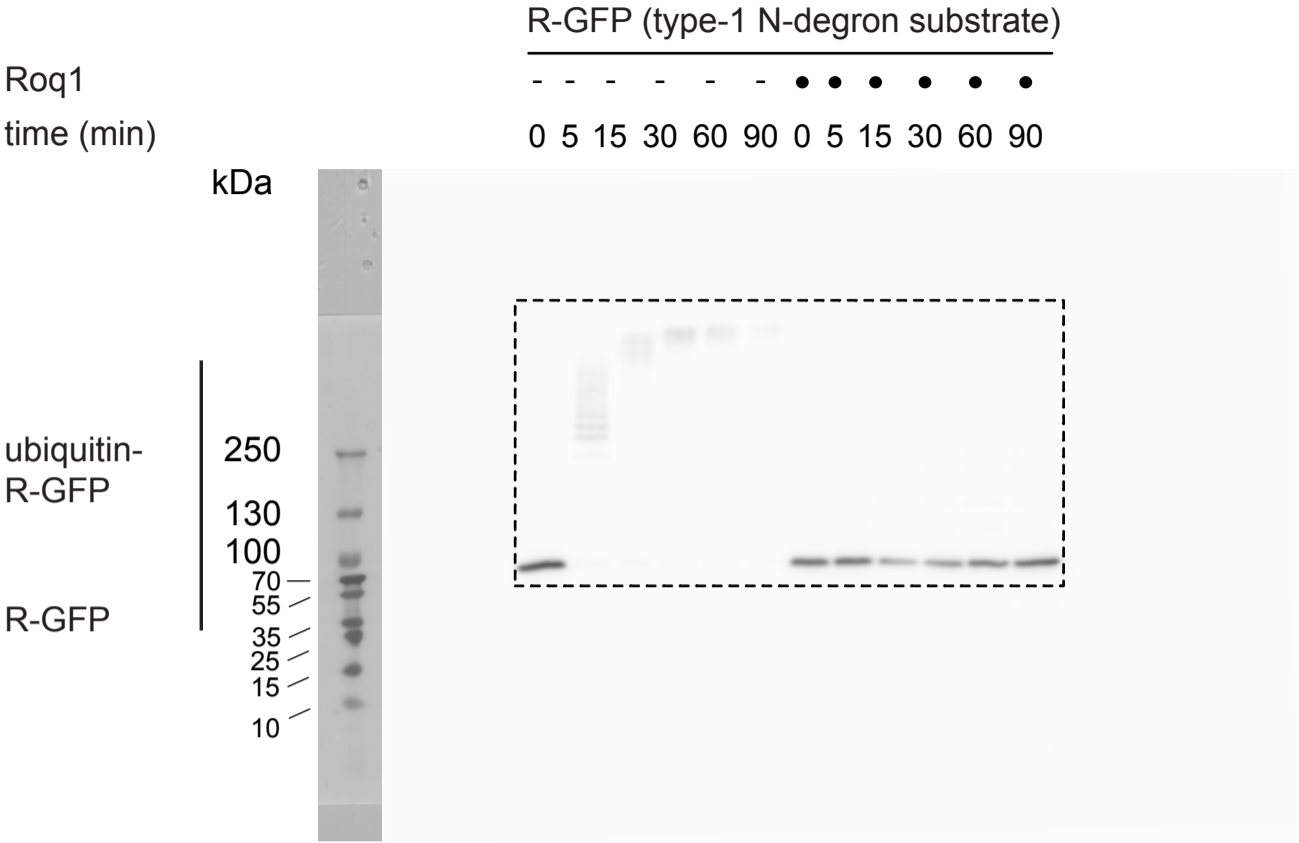

The boxed area was used for the final figure.

Supplement: Supplementary file 3 — Source data Fig. 1 [file 44318_2025_375_MOESM3_ESM.zip › Figure 1/Figure 1F.pdf]

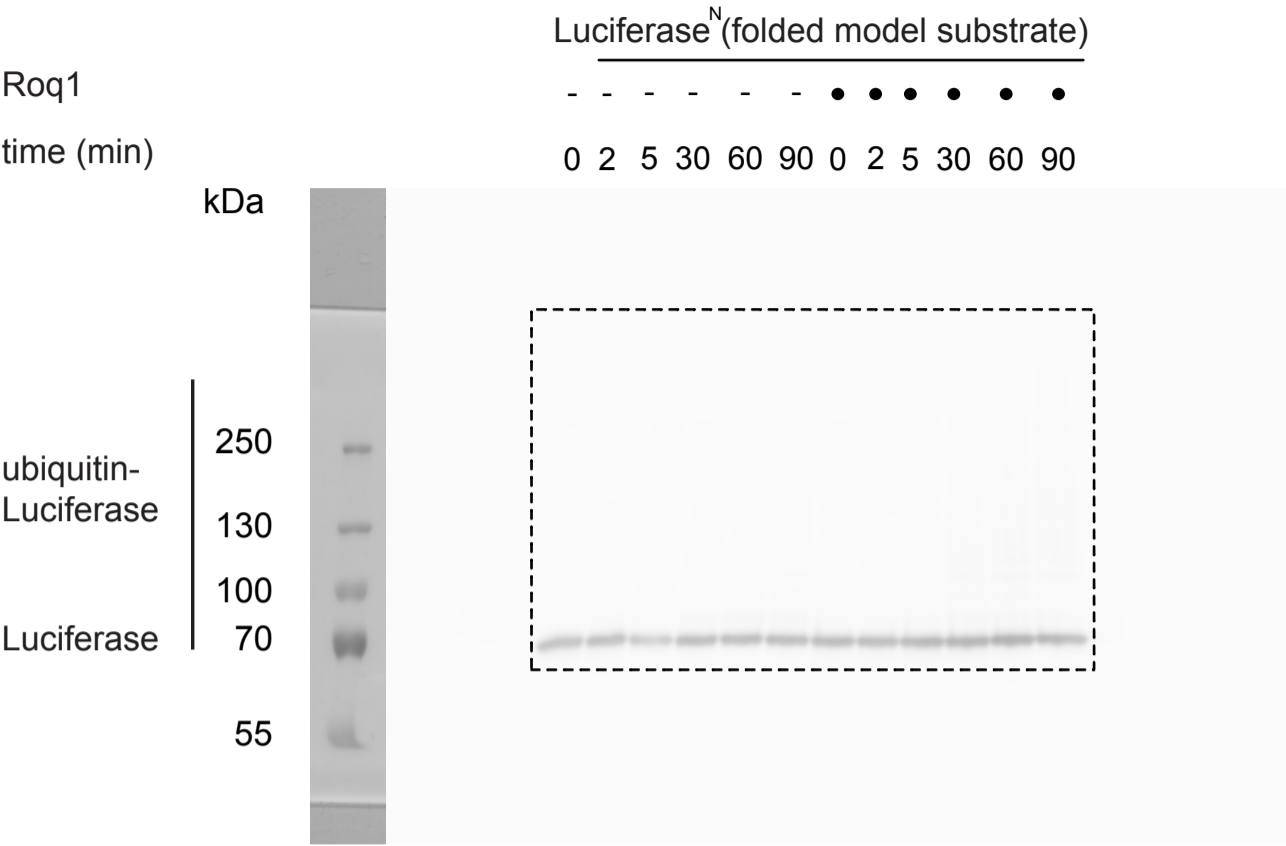

The boxed area was used for the final figure.

Supplement: Supplementary file 3 — Source data Fig. 1 [file 44318_2025_375_MOESM3_ESM.zip › Figure 1/Figure 1E.pdf]

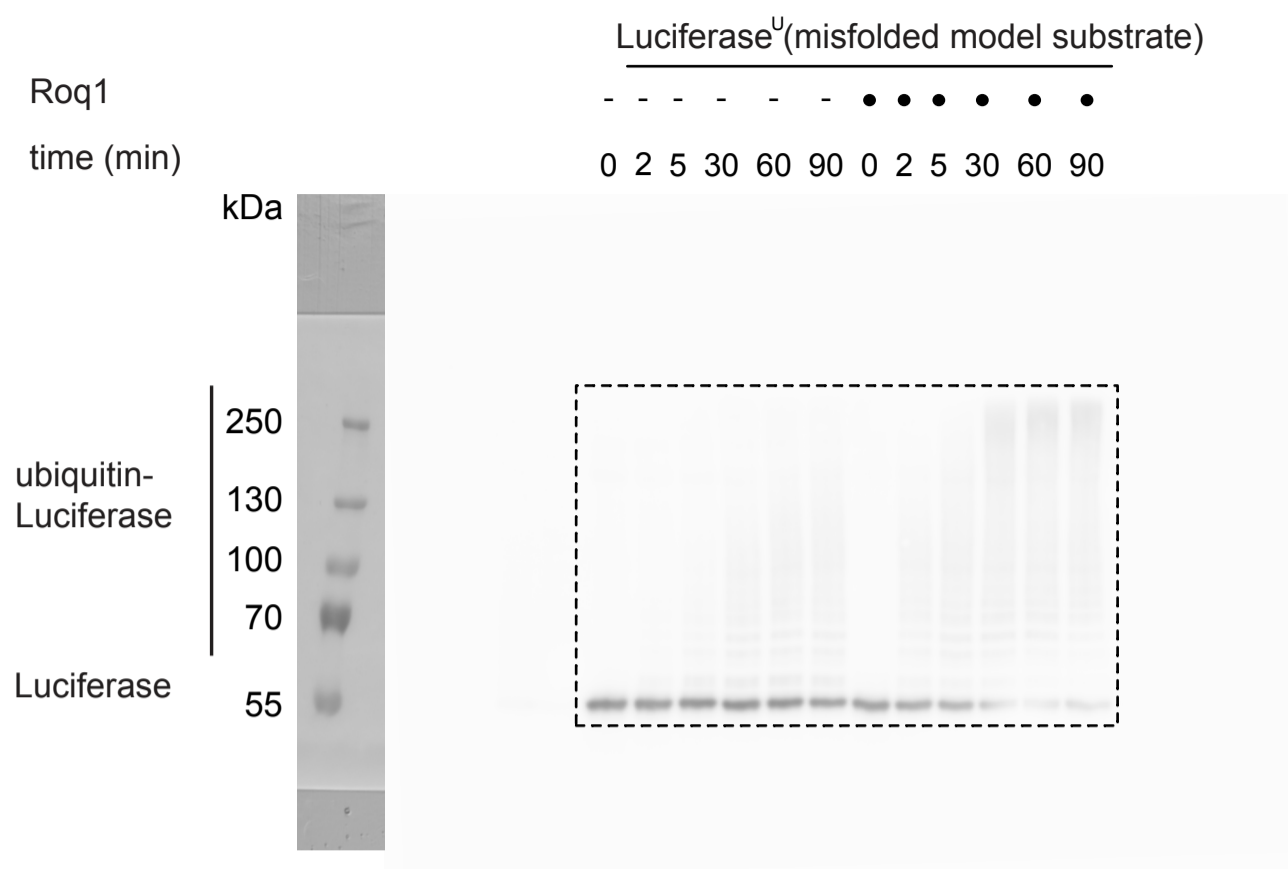

The boxed area was used for the final figure.

Supplement: Supplementary file 3 — Source data Fig. 1 [file 44318_2025_375_MOESM3_ESM.zip › Figure 1/Figure 1D.pdf]

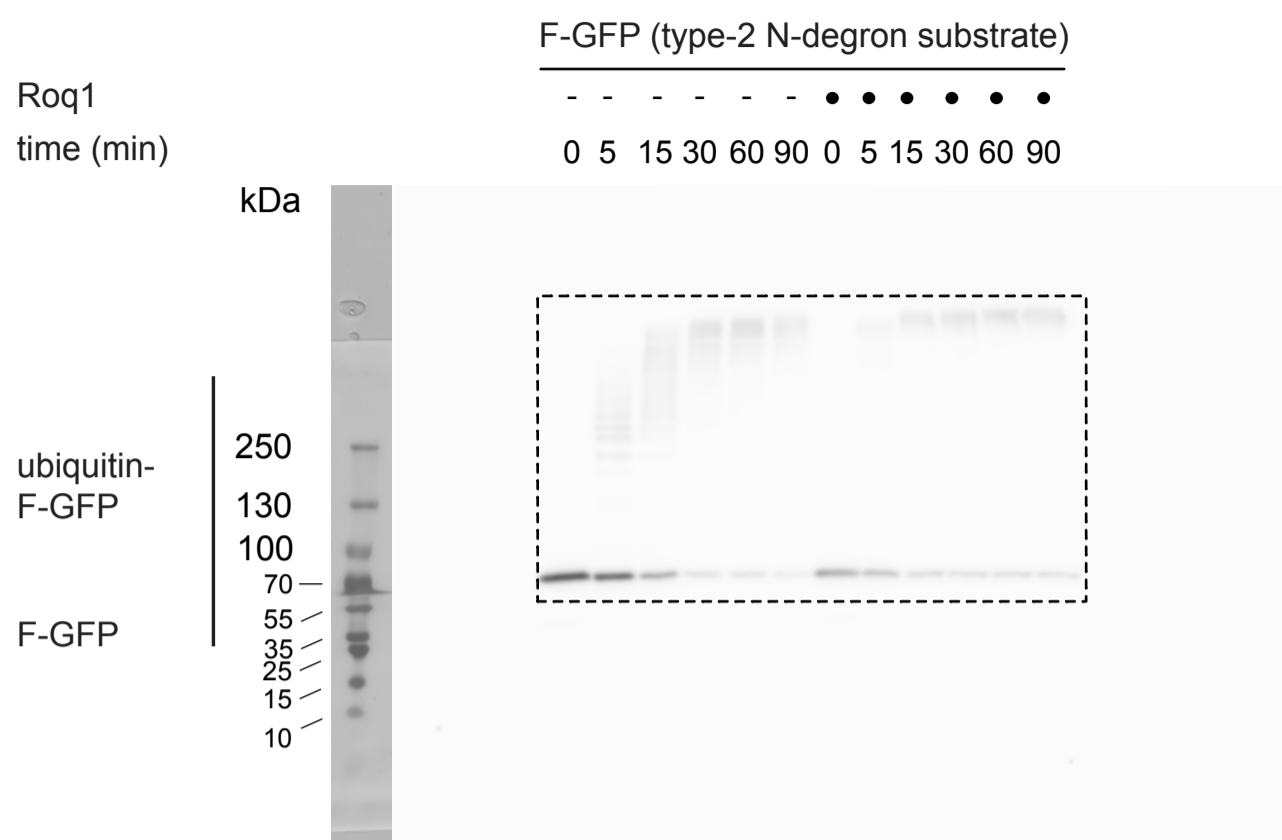

The boxed area was used for the final figure.

Supplement: Supplementary file 3 — Source data Fig. 1 [file 44318_2025_375_MOESM3_ESM.zip › Figure 1/Figure 1G.pdf]

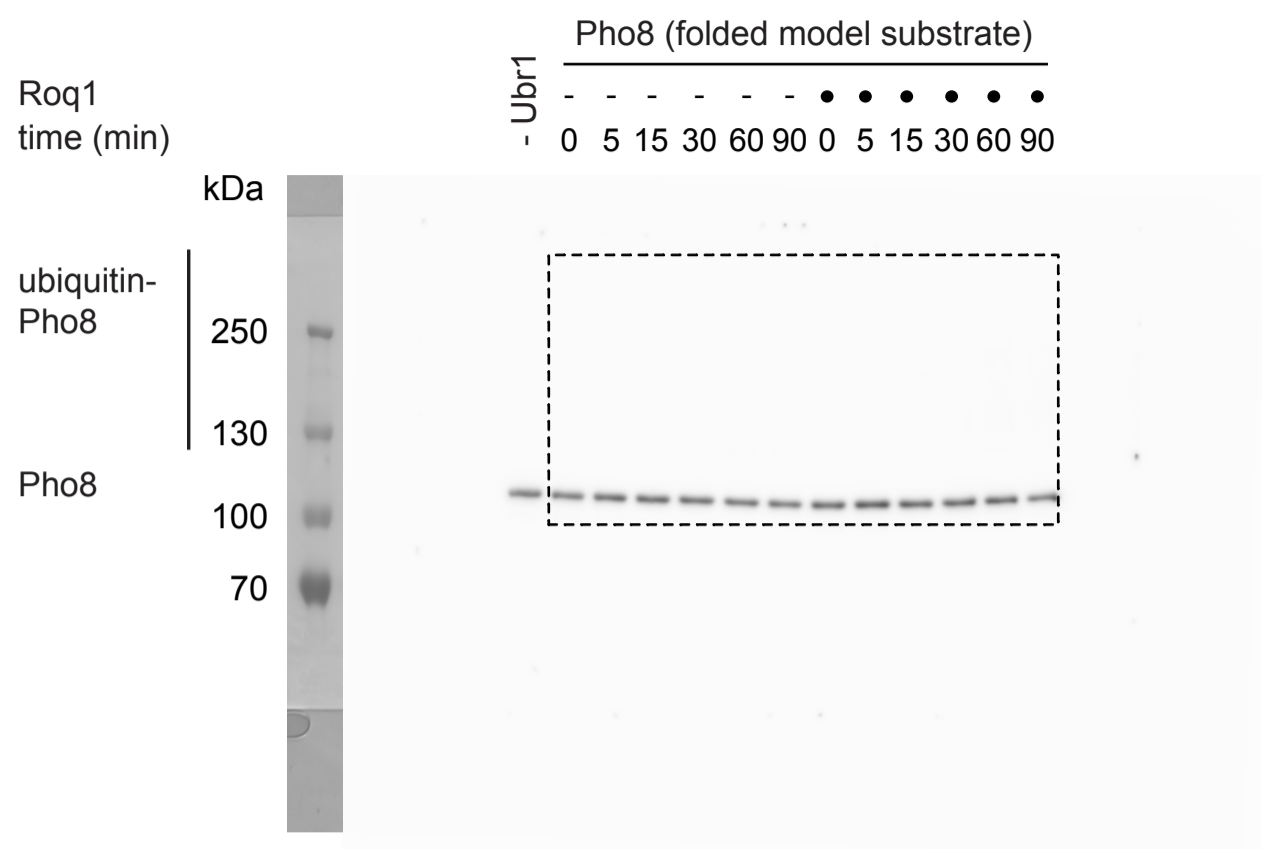

The boxed area was used for the final figure.

Supplement: Supplementary file 3 — Source data Fig. 1 [file 44318_2025_375_MOESM3_ESM.zip › Figure 1/Figure 1C.pdf]

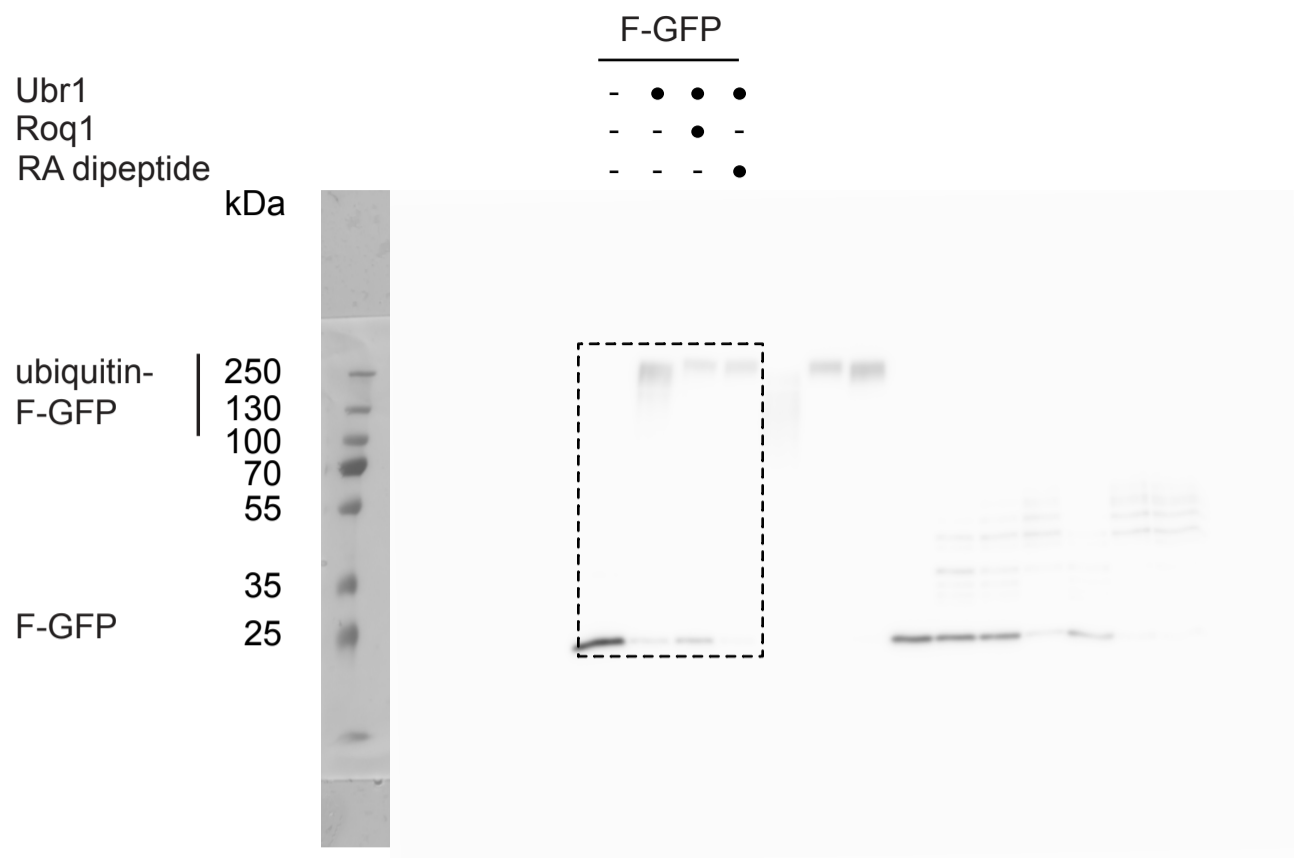

The boxed area was used for the final figure.

Supplement: Supplementary file 4 — Source data Fig. 2 [file 44318_2025_375_MOESM4_ESM.zip › Figure 2/Figure 2B.pdf]

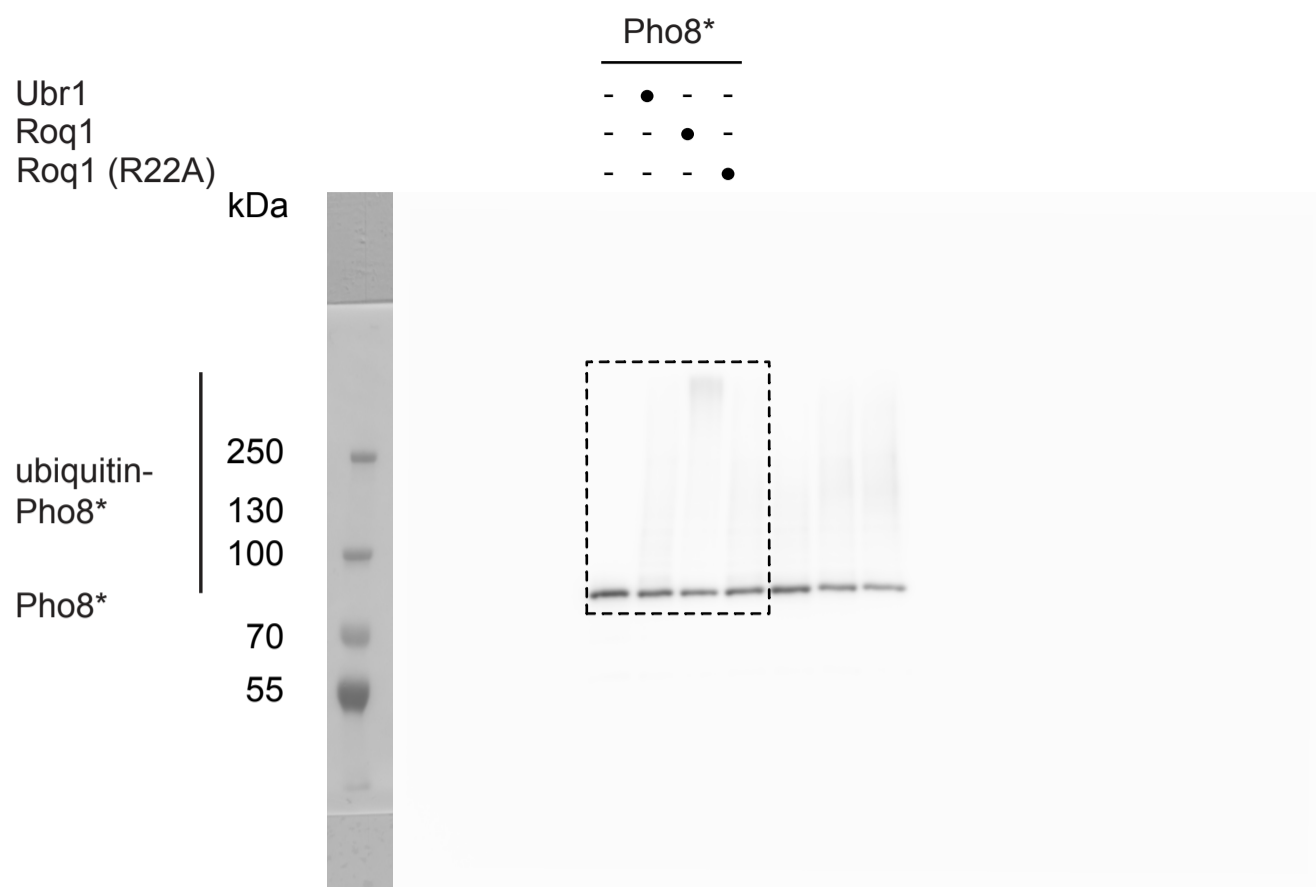

The boxed area was used for the final figure.

Supplement: Supplementary file 4 — Source data Fig. 2 [file 44318_2025_375_MOESM4_ESM.zip › Figure 2/Figure 2A.pdf]

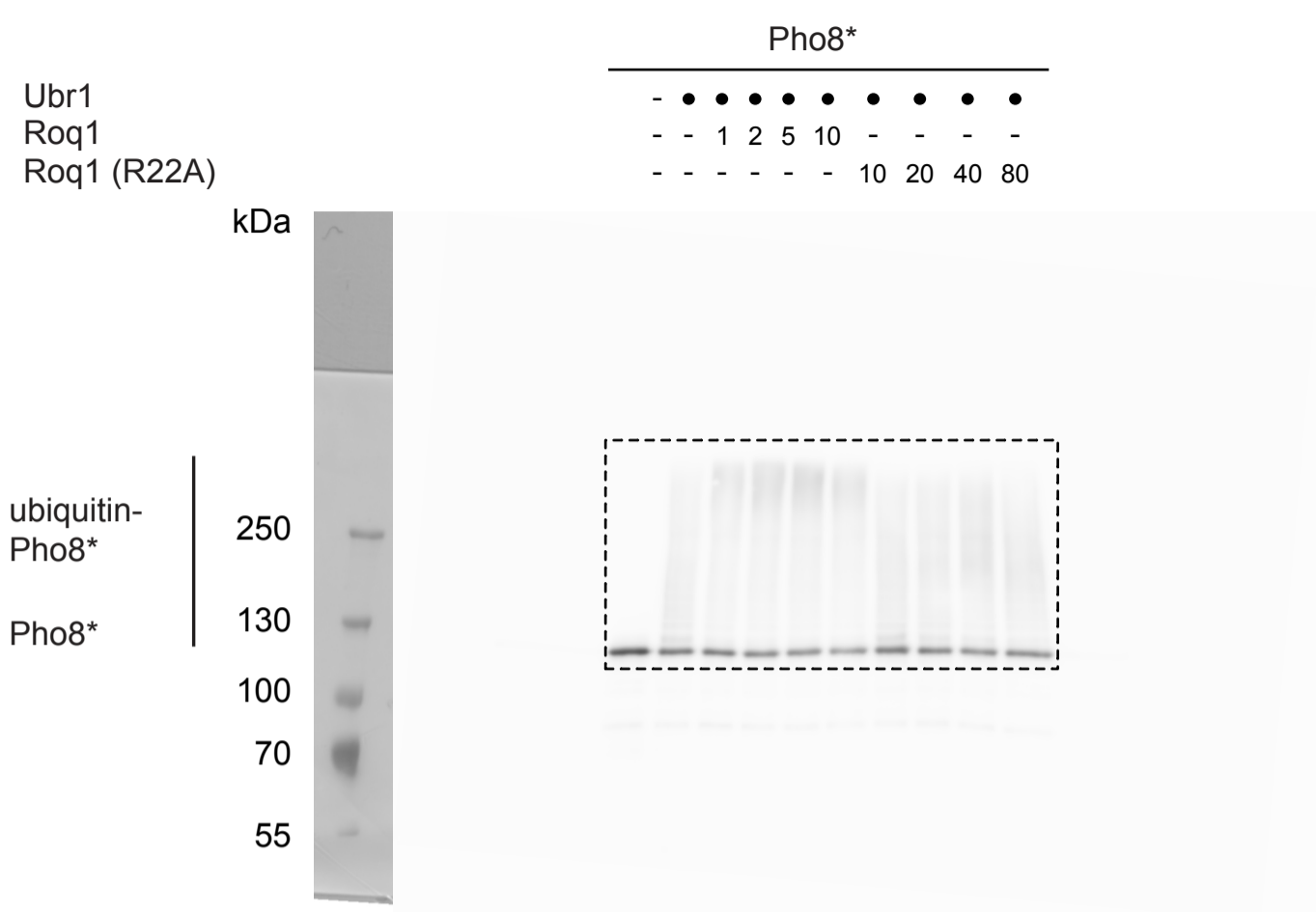

The boxed area was used for the final figure.

Supplement: Supplementary file 4 — Source data Fig. 2 [file 44318_2025_375_MOESM4_ESM.zip › Figure 2/Figure 2E.pdf]

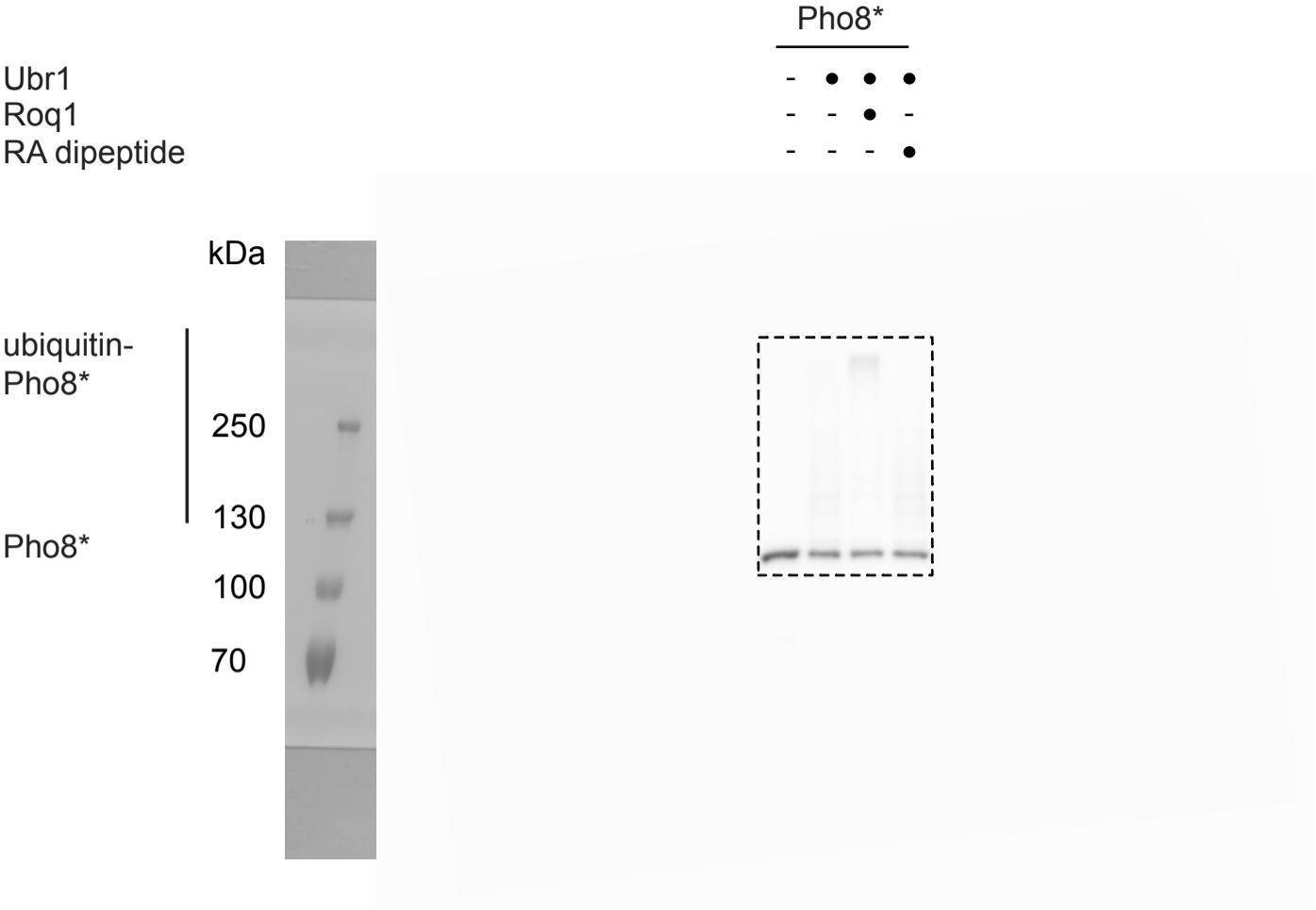

The boxed area was used for the final figure.

Supplement: Supplementary file 4 — Source data Fig. 2 [file 44318_2025_375_MOESM4_ESM.zip › Figure 2/Figure 2C.pdf]

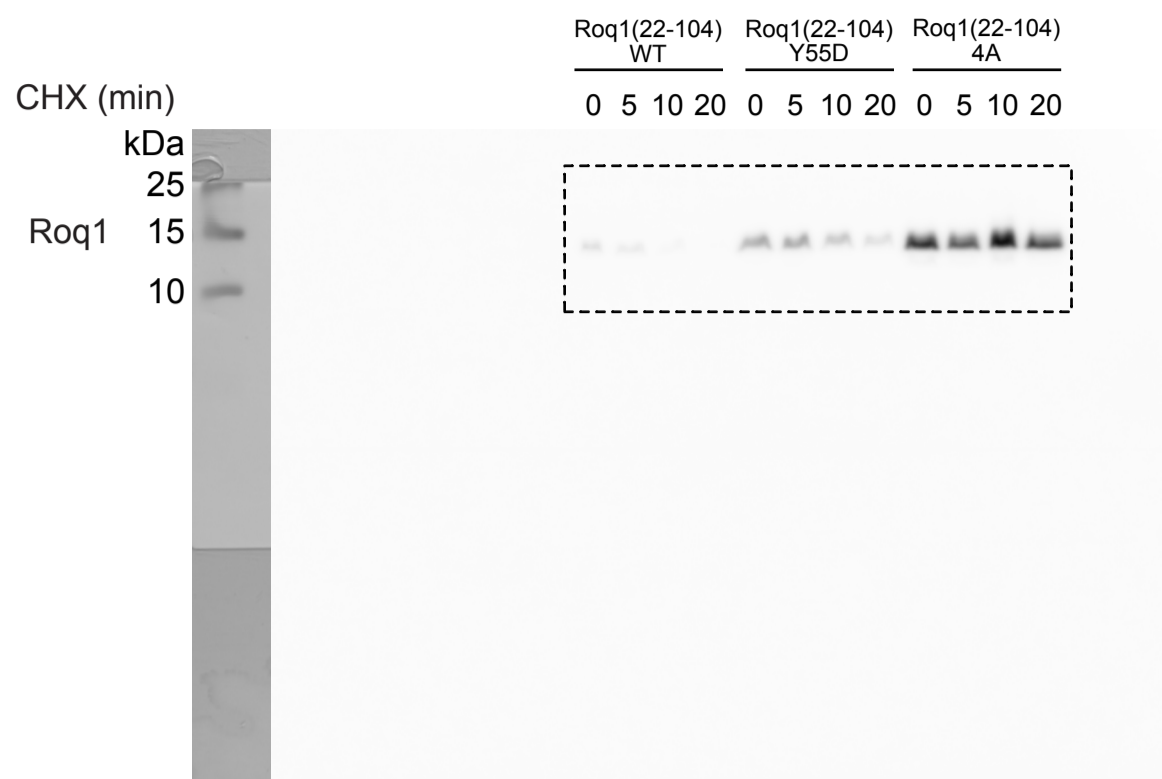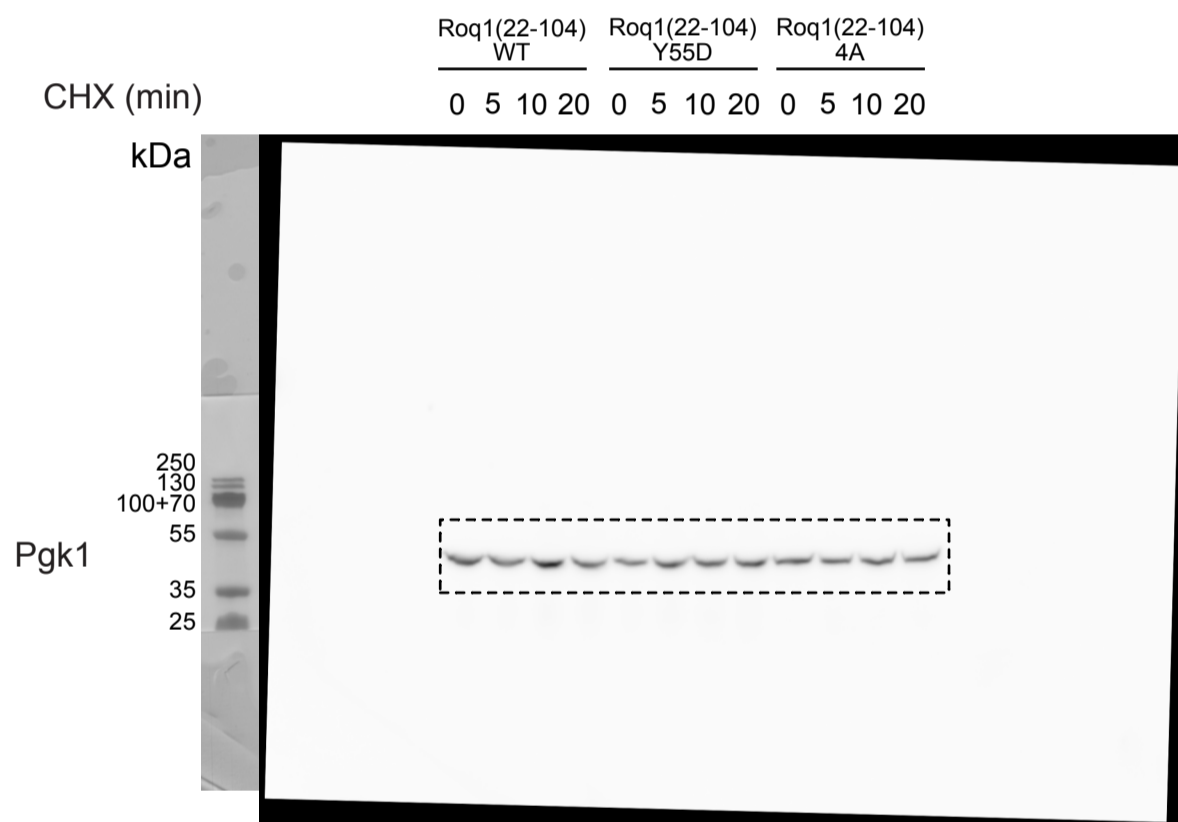

The boxed area was used for the final figure.

Supplement: Supplementary file 5 — Source data Fig. 3 [file 44318_2025_375_MOESM5_ESM.zip › Figure 3/Figure 3F.pdf]

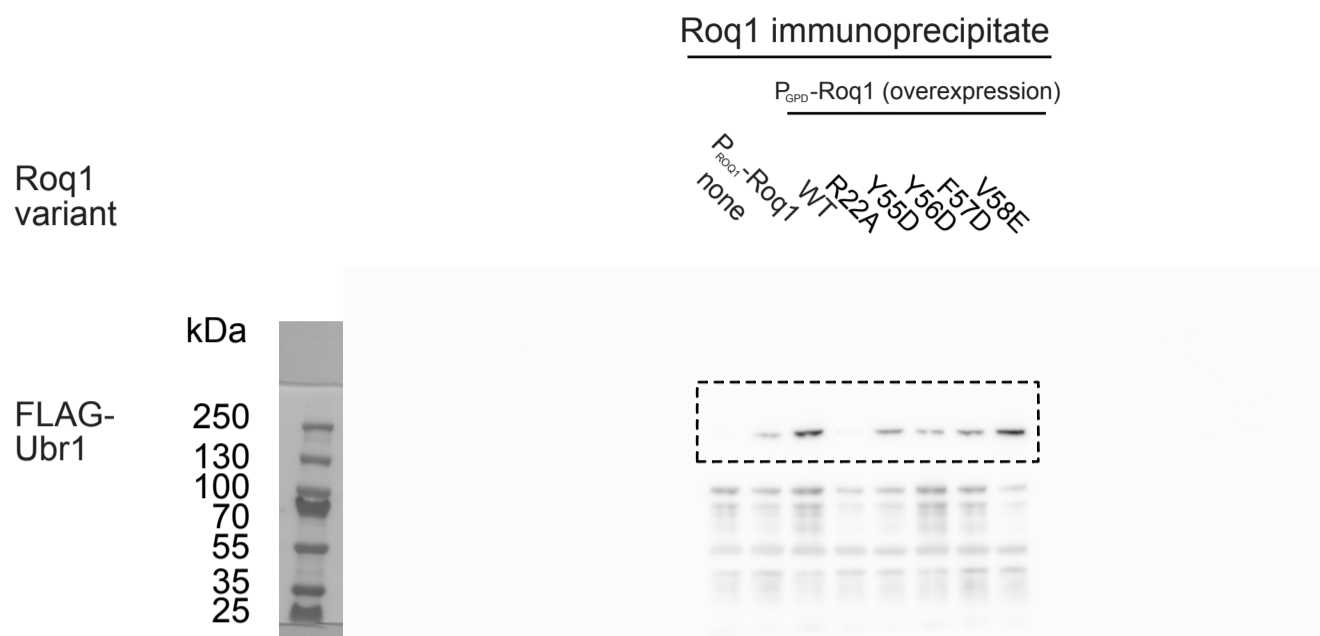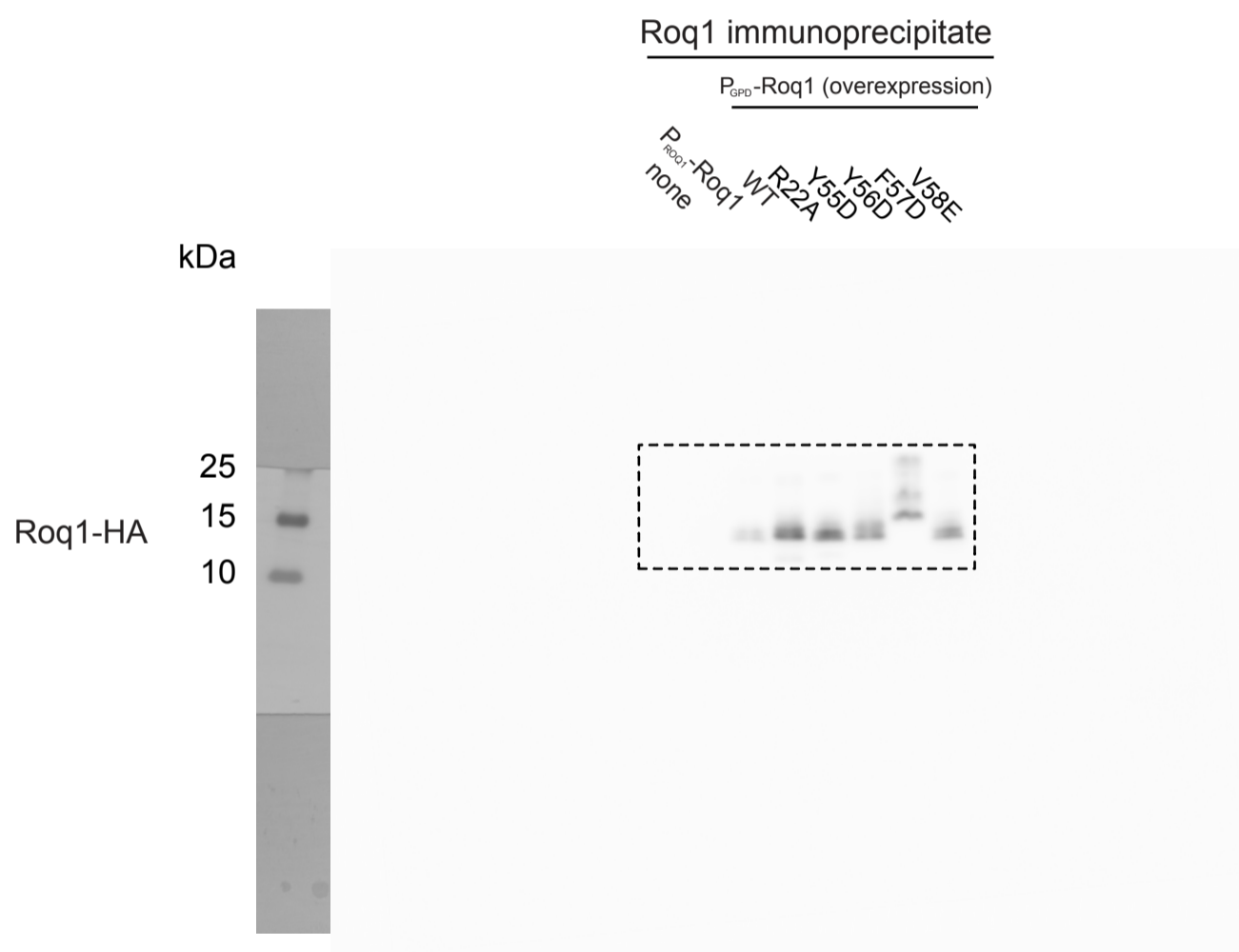

The boxed area was used for the final figure.

Supplement: Supplementary file 6 — Source data Fig. 4 [file 44318_2025_375_MOESM6_ESM.zip › Figure 4/Figure 4A/Figure 4A immunoprecipitates.pdf]

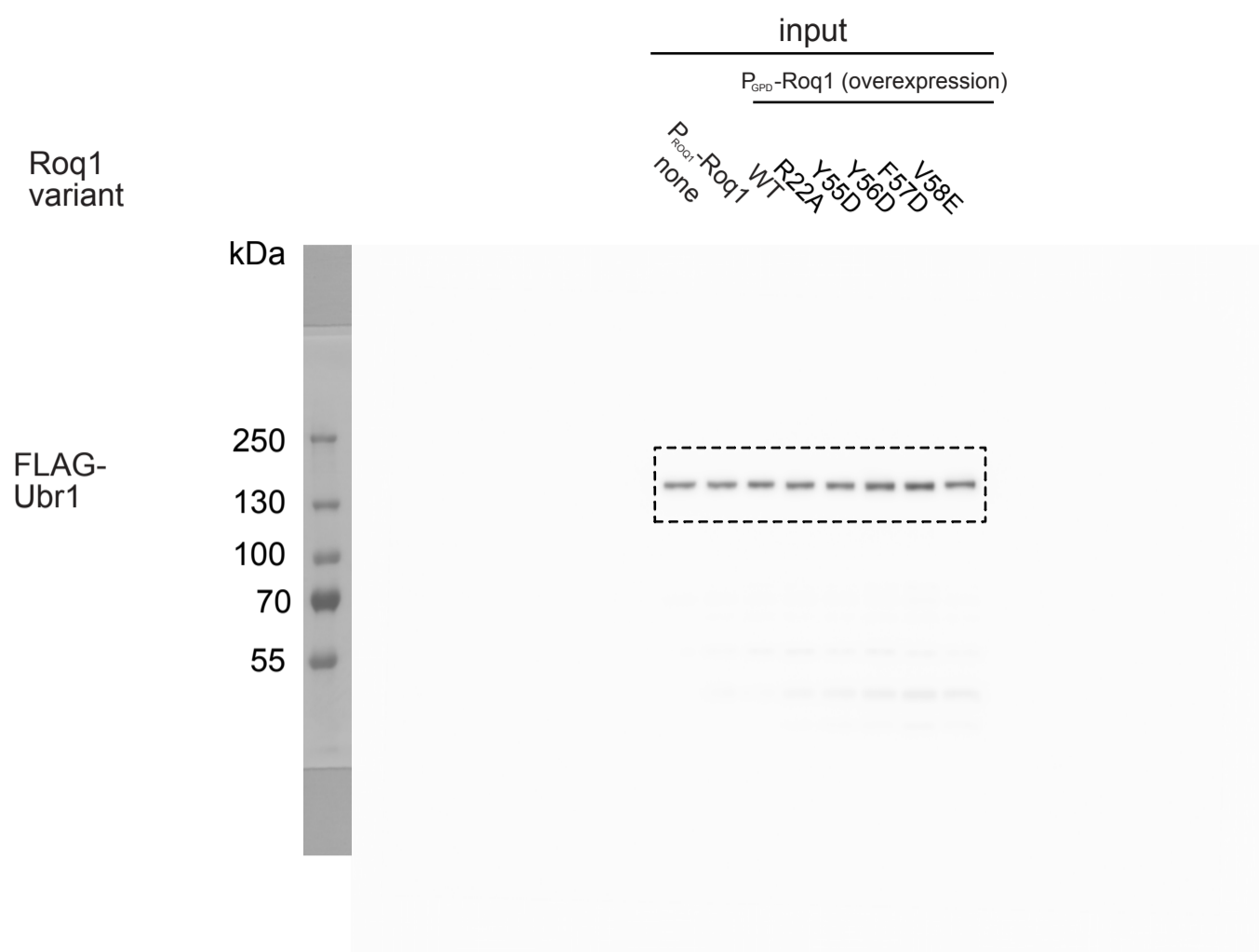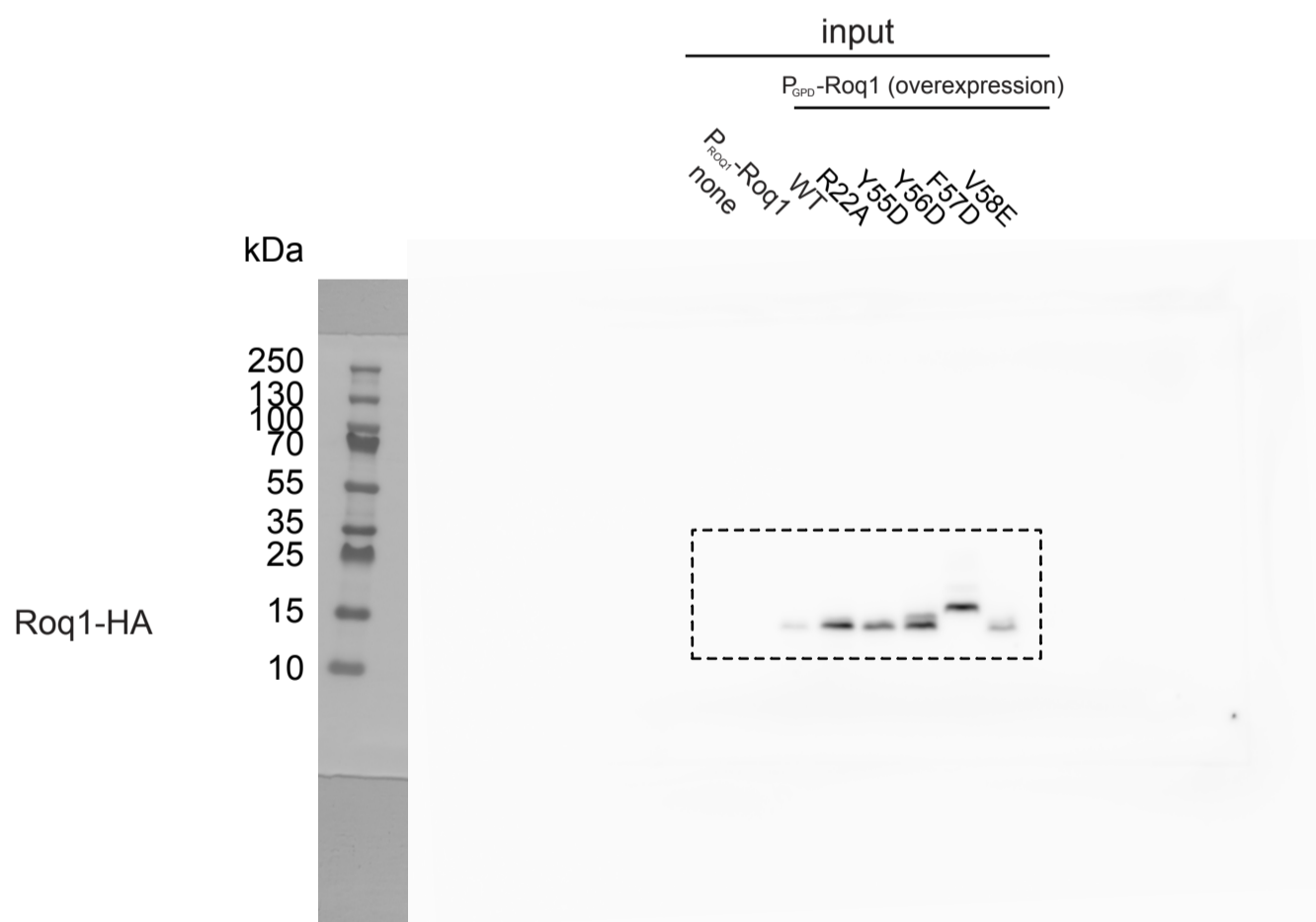

The boxed area was used for the final figure.

Supplement: Supplementary file 6 — Source data Fig. 4 [file 44318_2025_375_MOESM6_ESM.zip › Figure 4/Figure 4A/Figure 4A inputs.pdf]

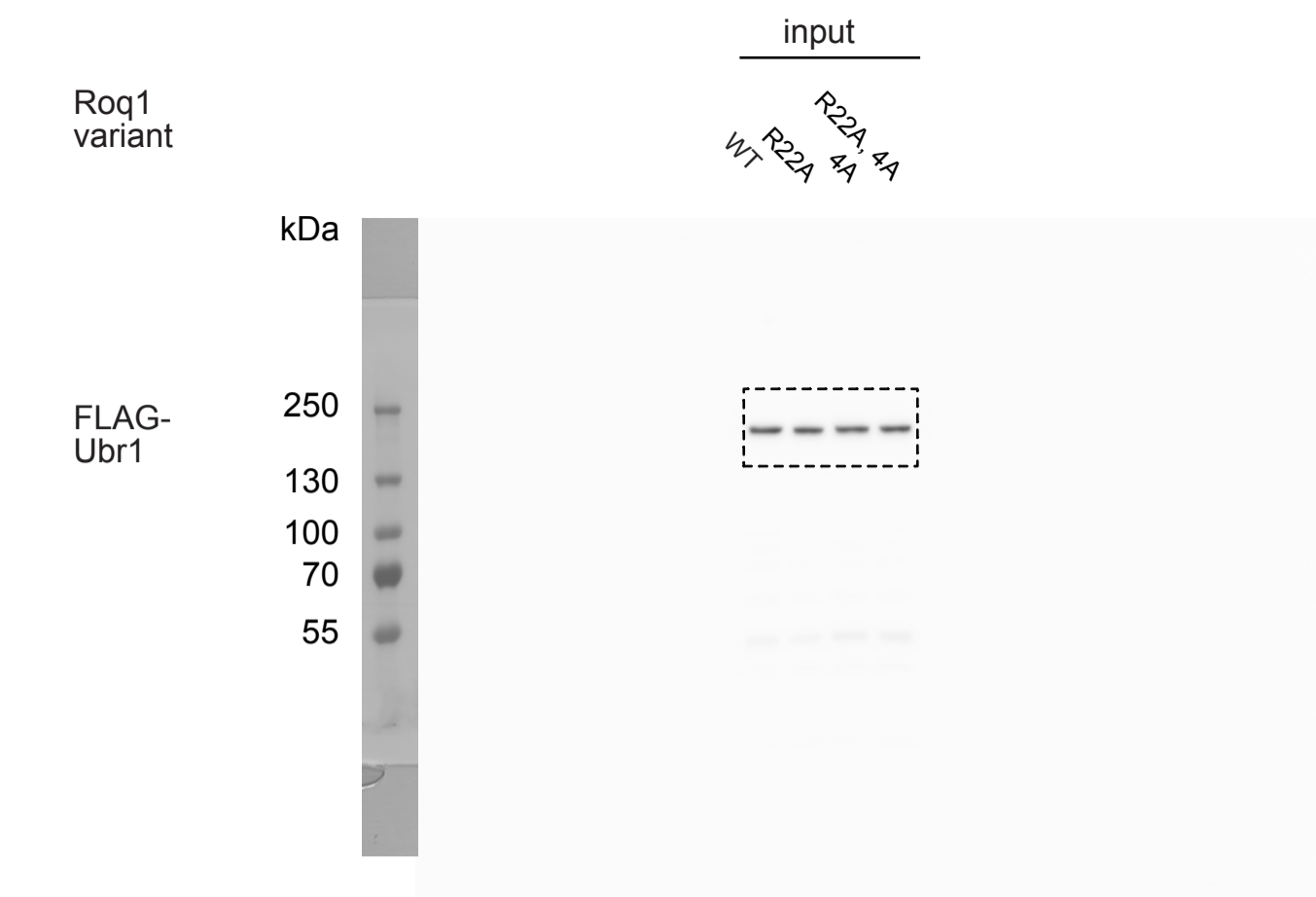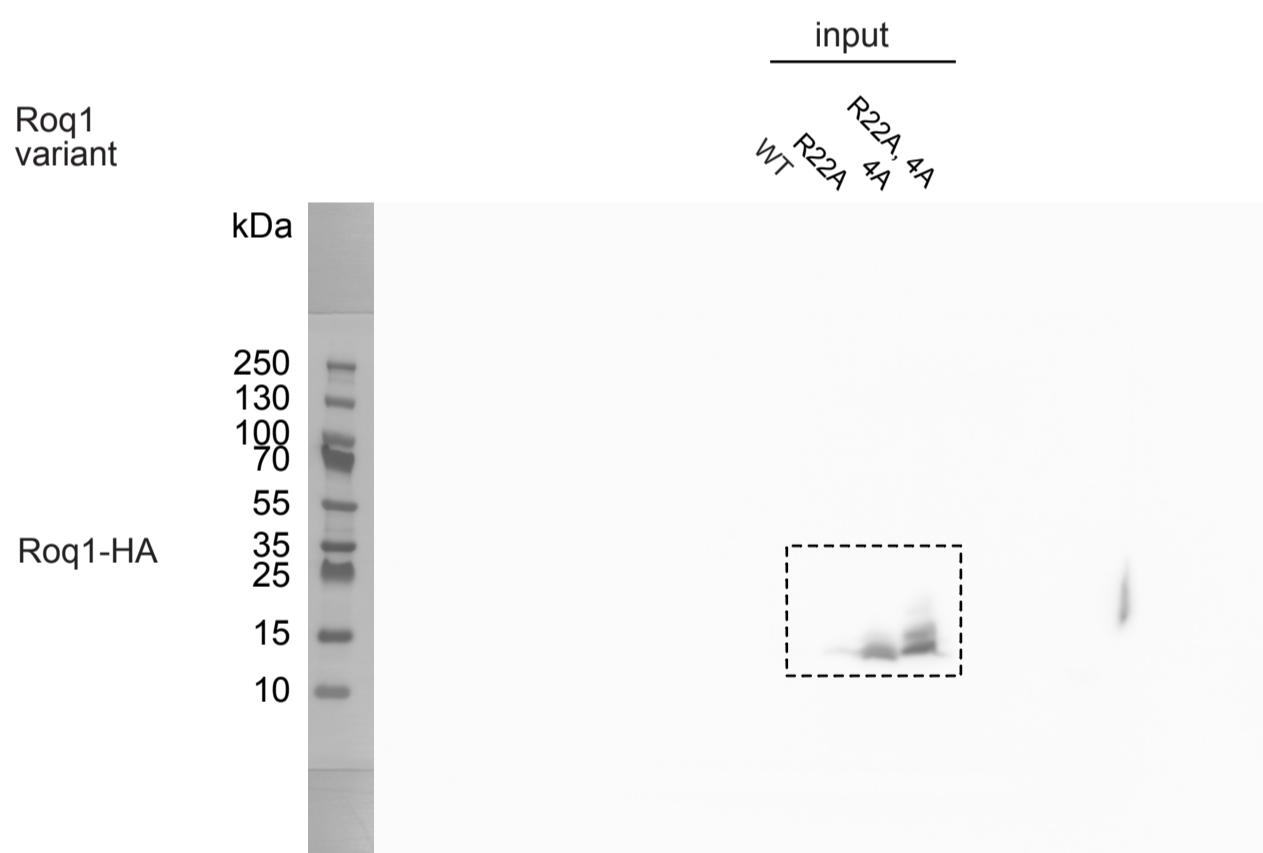

The boxed area was used for the final figure.

Supplement: Supplementary file 6 — Source data Fig. 4 [file 44318_2025_375_MOESM6_ESM.zip › Figure 4/Figure 4B/Figure 4B inputs.pdf]

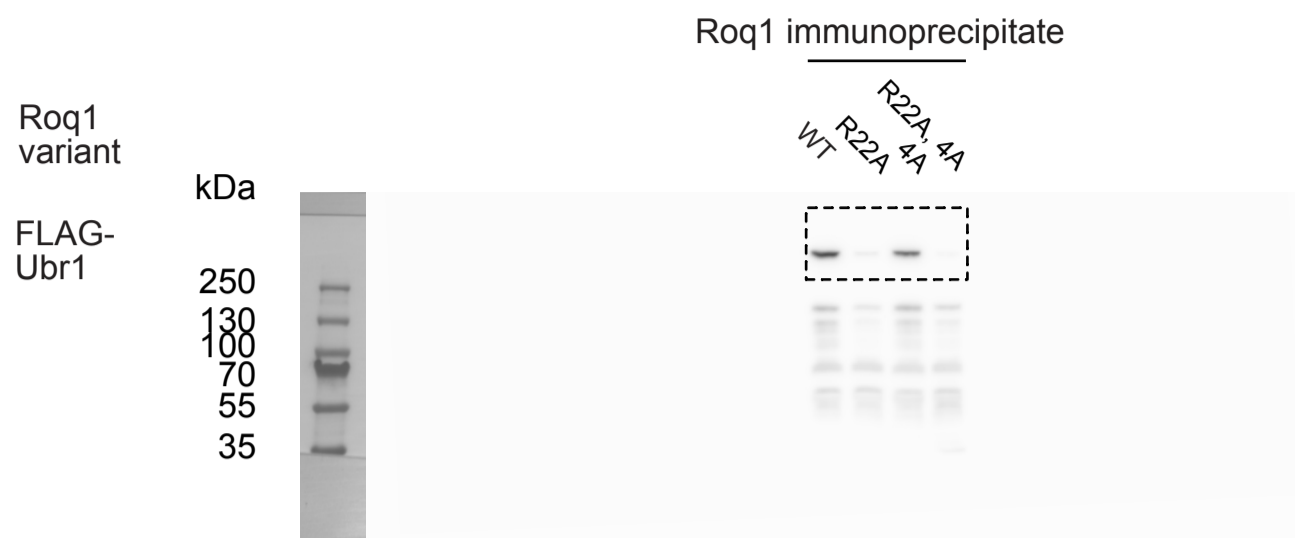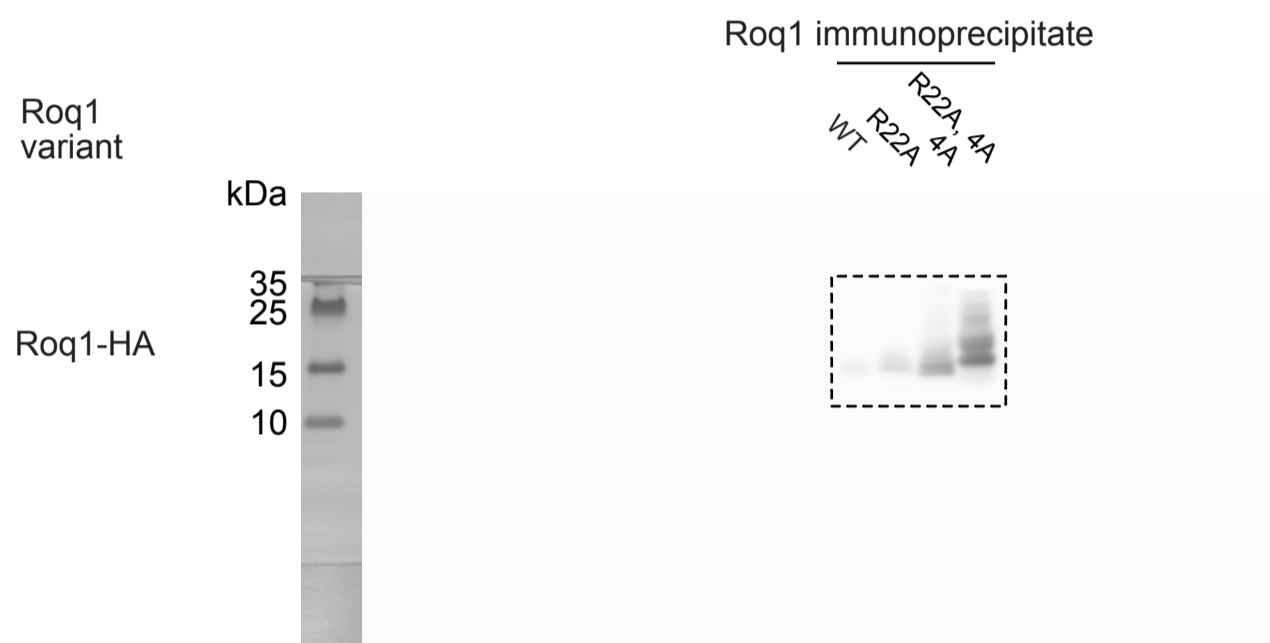

The boxed area was used for the final figure.

Supplement: Supplementary file 6 — Source data Fig. 4 [file 44318_2025_375_MOESM6_ESM.zip › Figure 4/Figure 4B/Figure 4B immunoprecipitates.pdf]

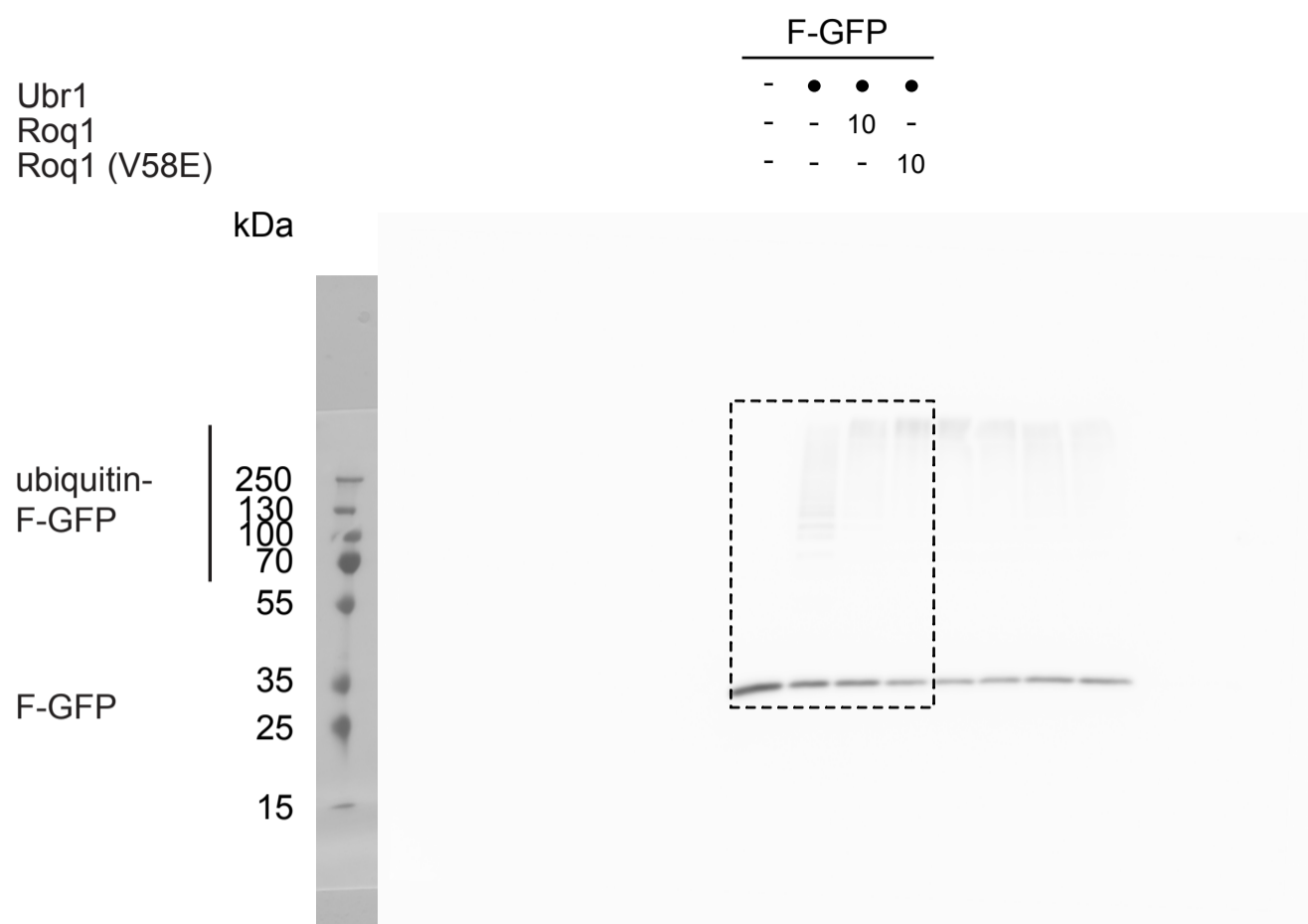

The boxed area was used for the final figure.

Supplement: Supplementary file 7 — Source data Fig. 5 [file 44318_2025_375_MOESM7_ESM.zip › Figure 5/Figure 5E.pdf]

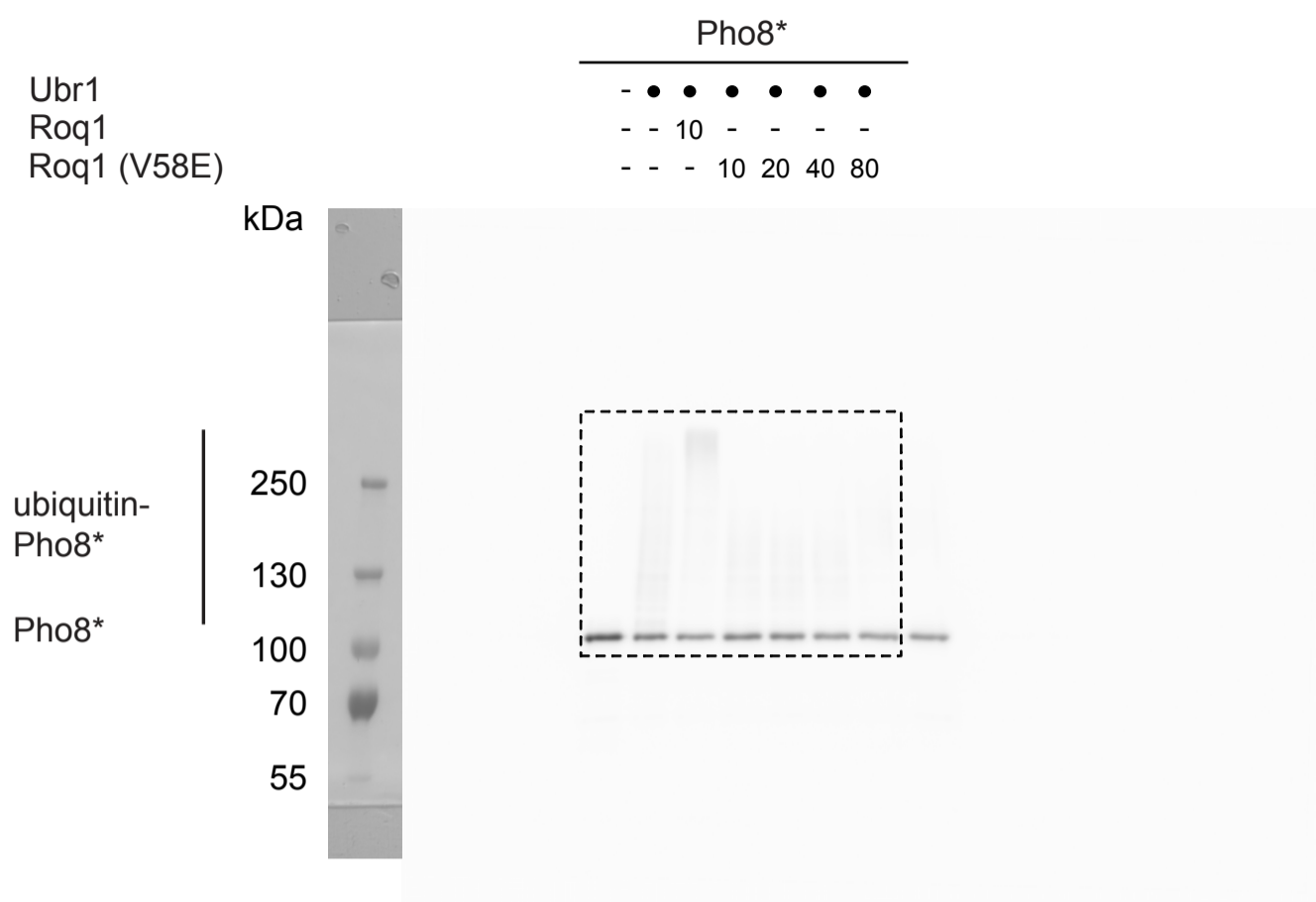

The boxed area was used for the final figure.

Supplement: Supplementary file 7 — Source data Fig. 5 [file 44318_2025_375_MOESM7_ESM.zip › Figure 5/Figure 5B.pdf]

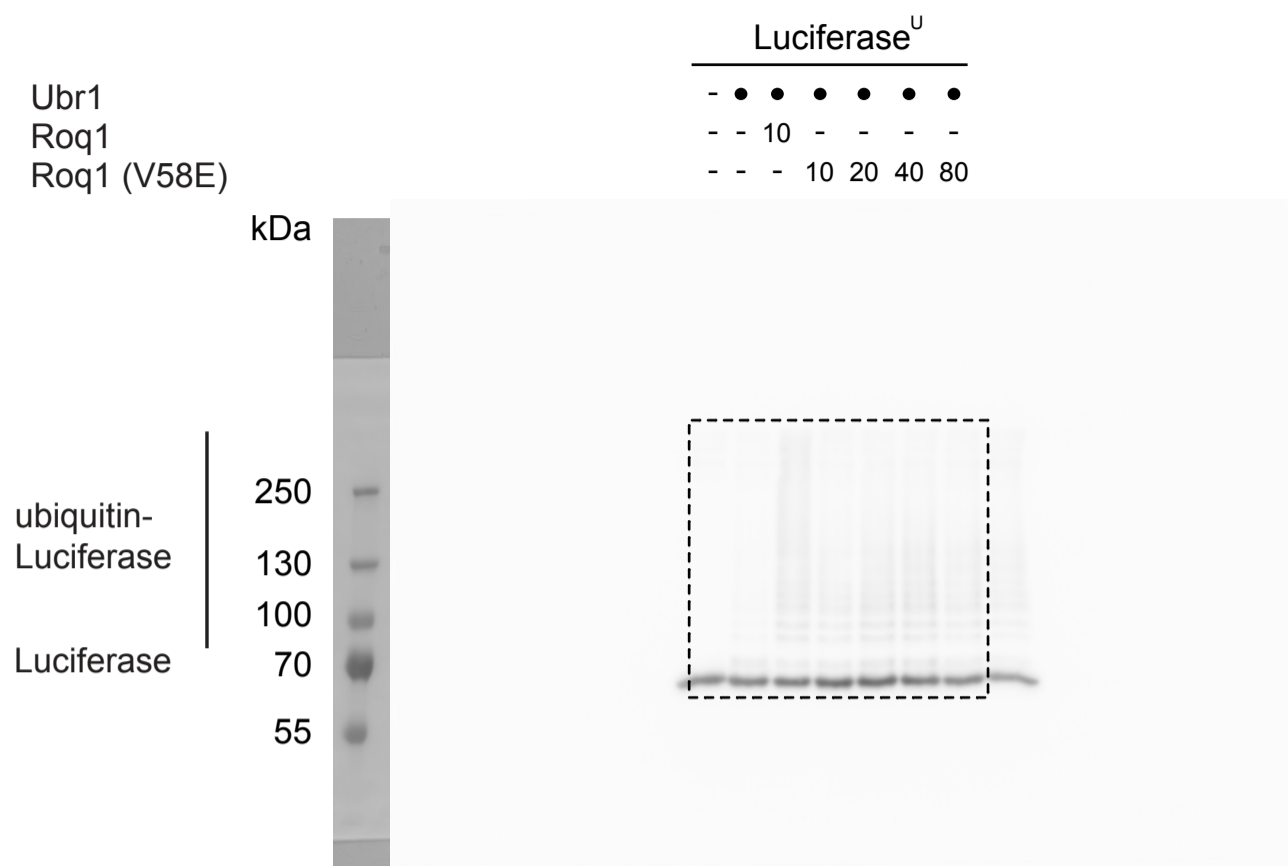

The boxed area was used for the final figure.

Supplement: Supplementary file 7 — Source data Fig. 5 [file 44318_2025_375_MOESM7_ESM.zip › Figure 5/Figure 5C.pdf]

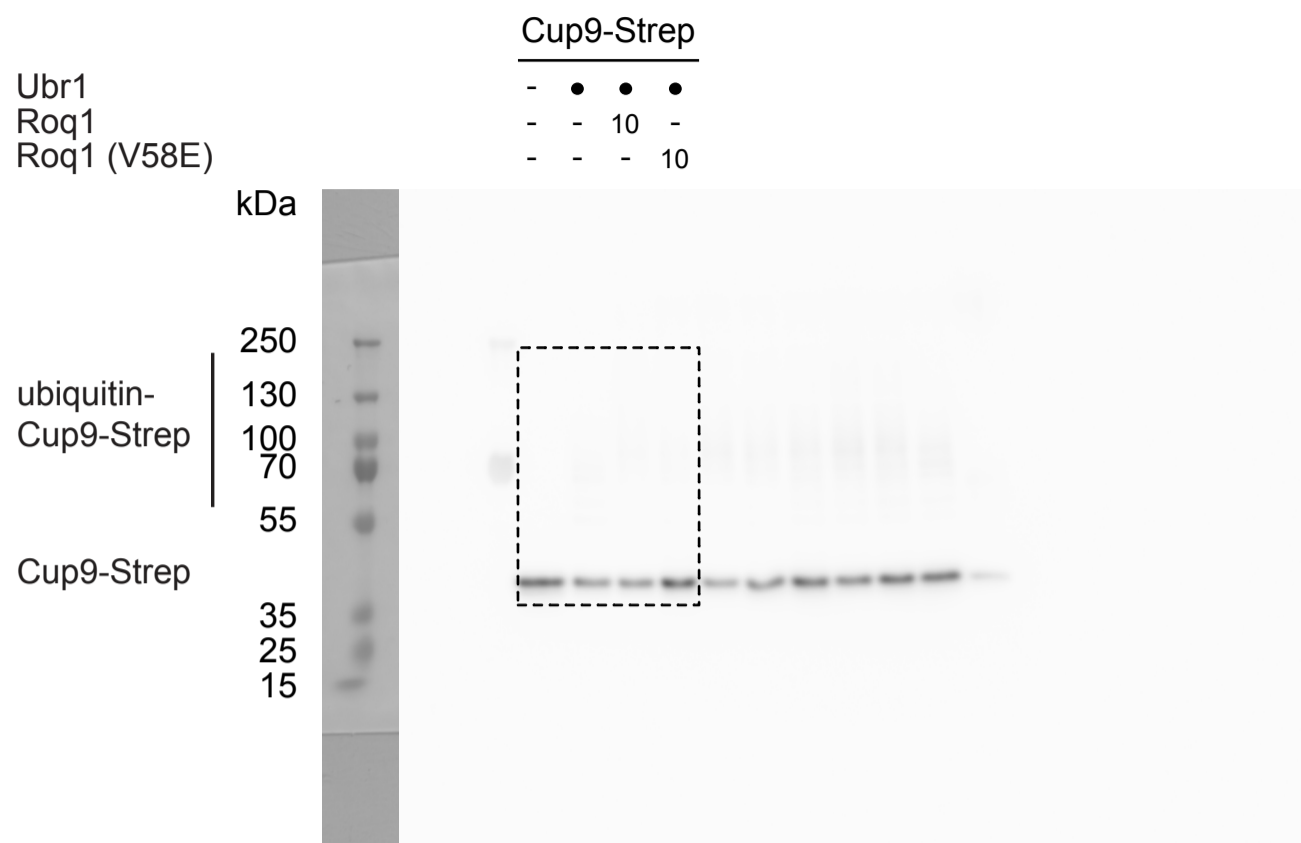

The boxed area was used for the final figure.

Supplement: Supplementary file 7 — Source data Fig. 5 [file 44318_2025_375_MOESM7_ESM.zip › Figure 5/Figure 5F.pdf]

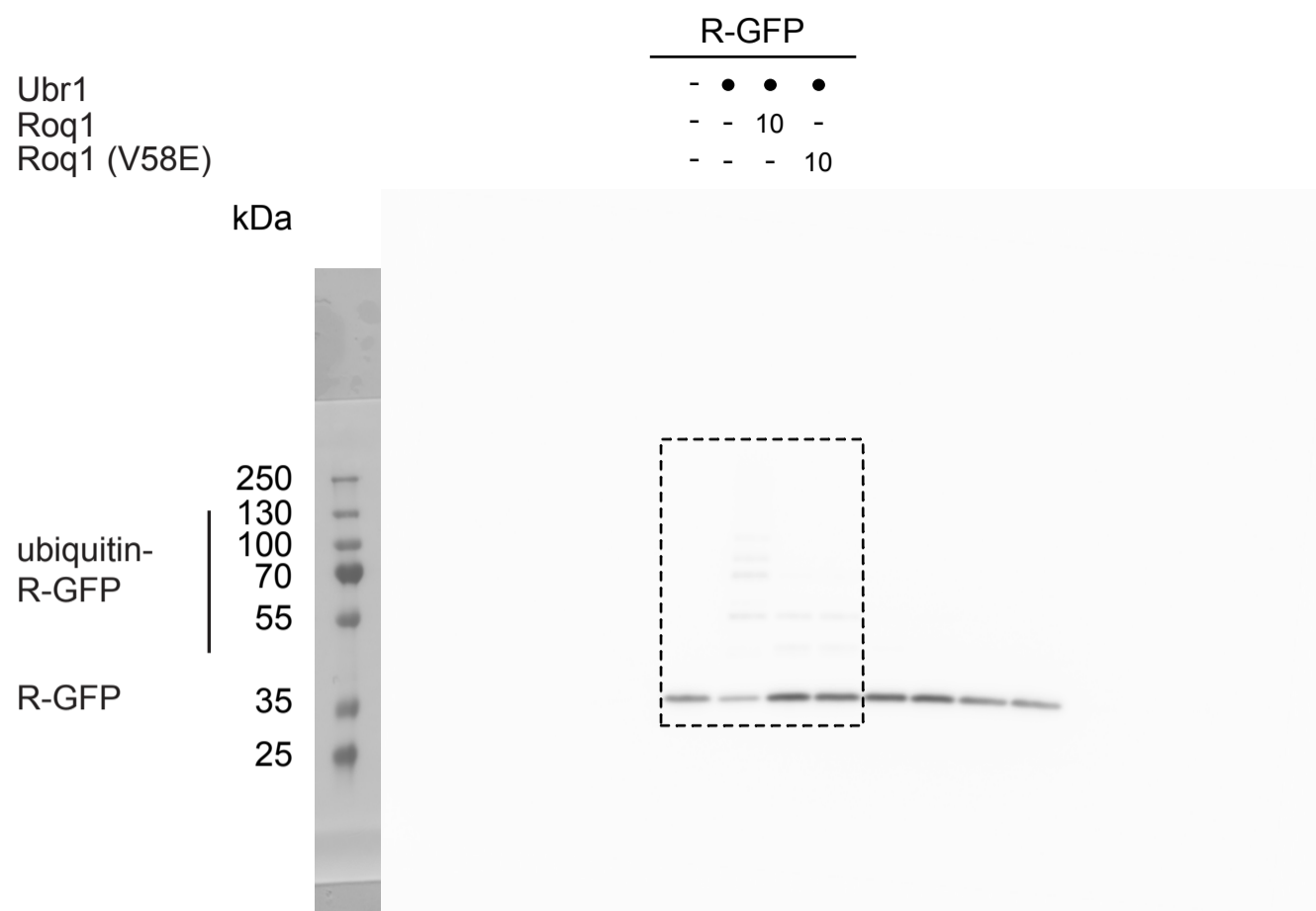

The boxed area was used for the final figure.

Supplement: Supplementary file 7 — Source data Fig. 5 [file 44318_2025_375_MOESM7_ESM.zip › Figure 5/Figure 5D.pdf]

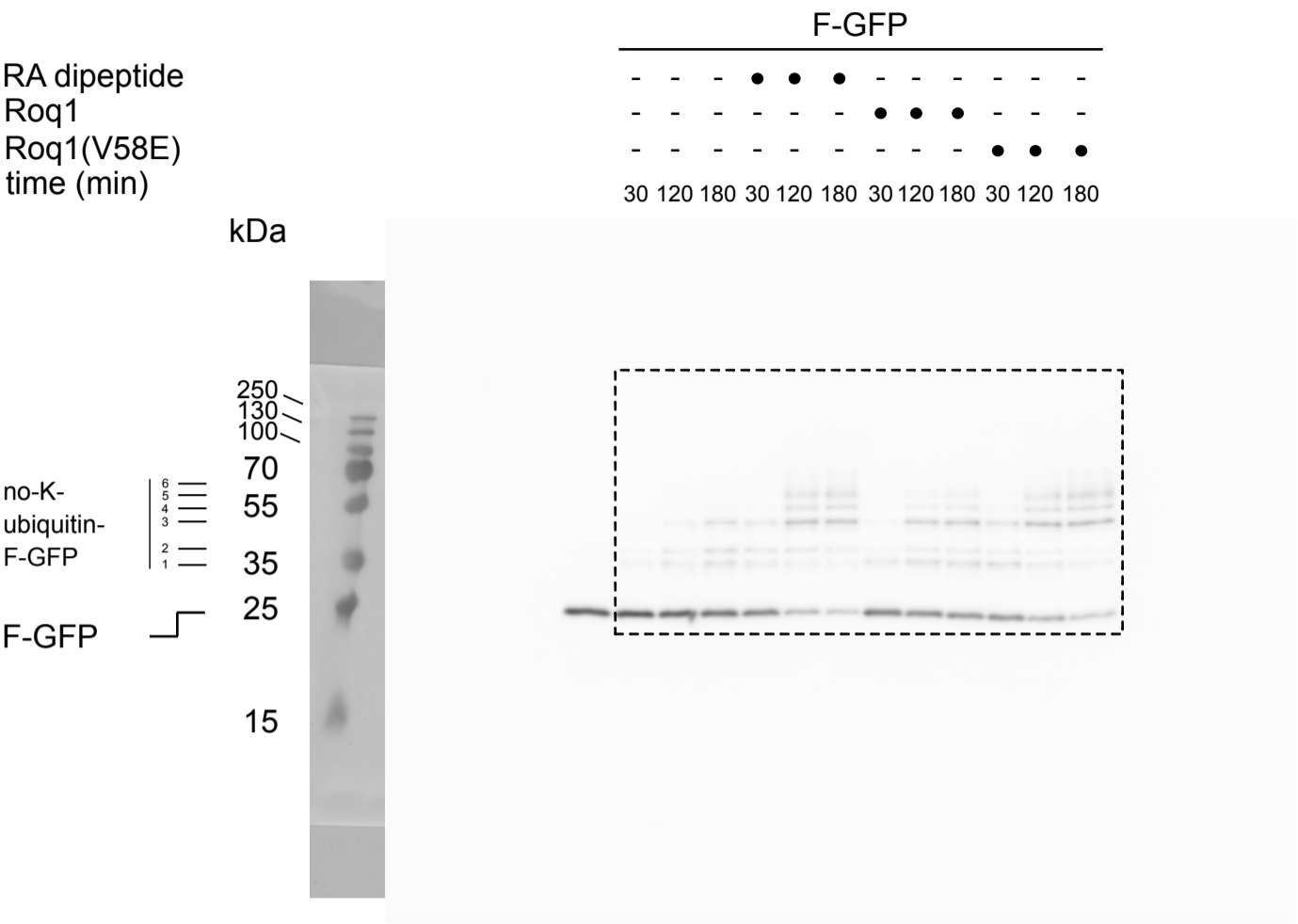

The boxed area was used for the final figure.

Supplement: Supplementary file 8 — Source data Fig. 6 [file 44318_2025_375_MOESM8_ESM.zip › Figure 6/Figure 6B.pdf]

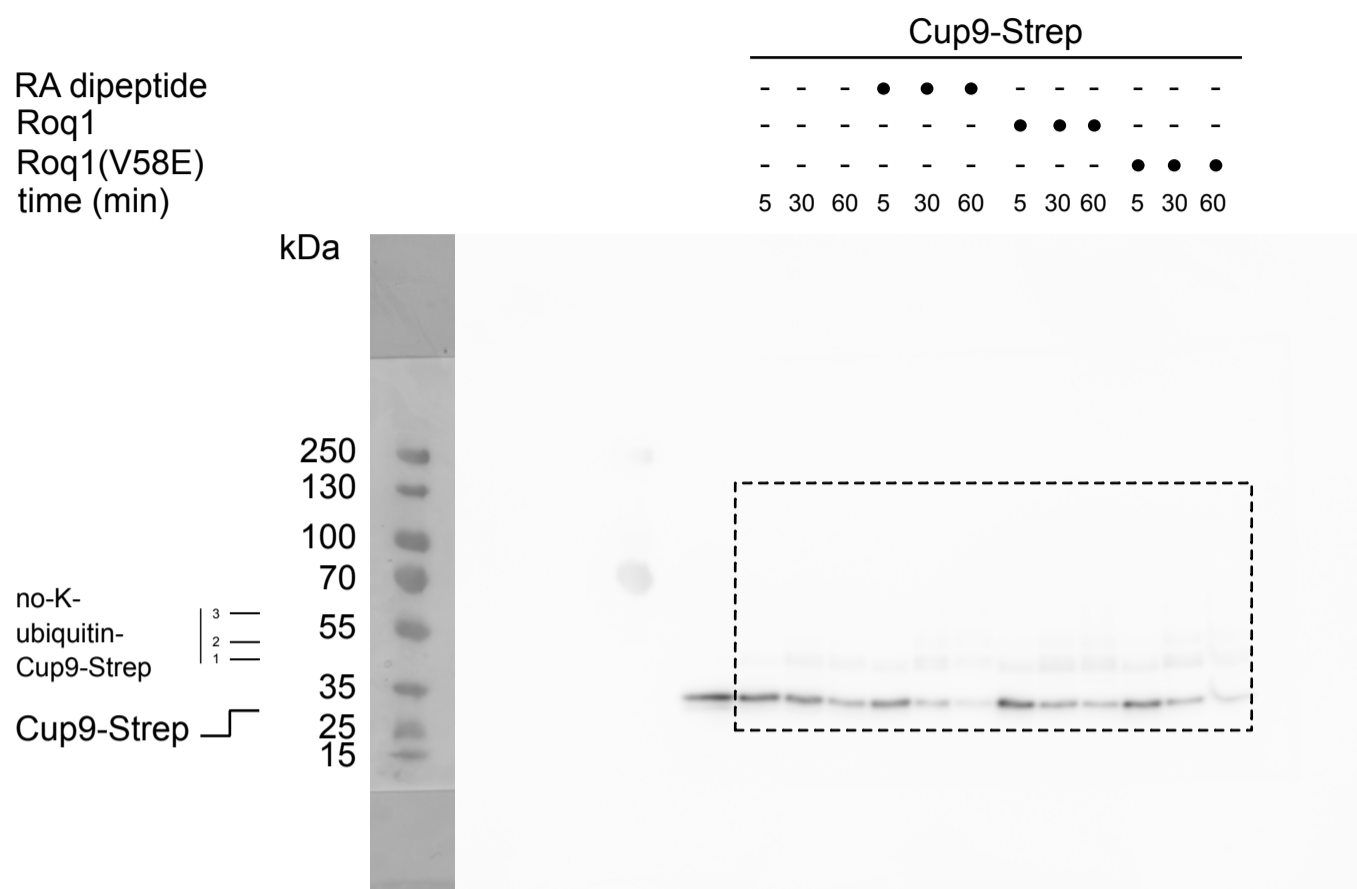

The boxed area was used for the final figure.

Supplement: Supplementary file 8 — Source data Fig. 6 [file 44318_2025_375_MOESM8_ESM.zip › Figure 6/Figure 6C.pdf]

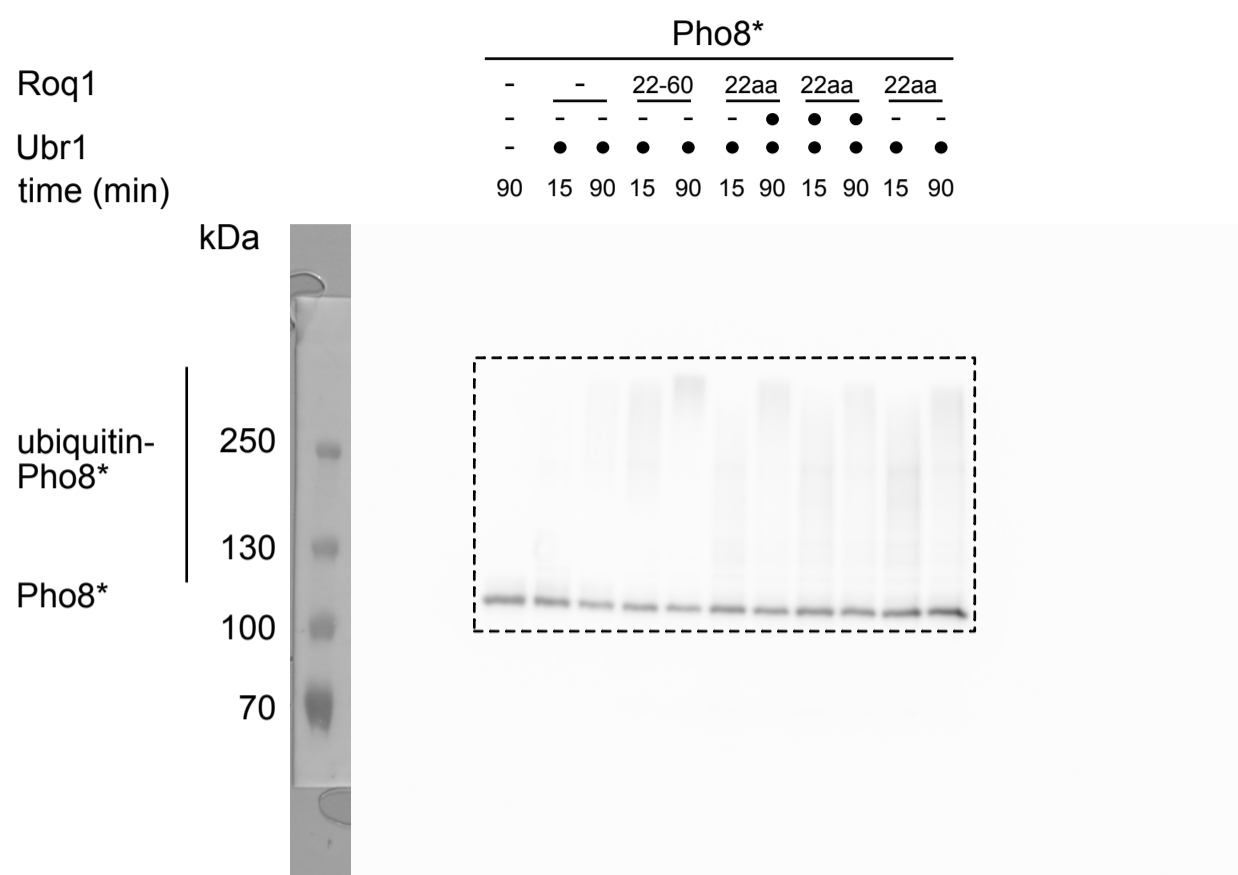

The boxed area was used for the final figure.

Supplement: Supplementary file 10 — Source data Fig. 8 [file 44318_2025_375_MOESM10_ESM.zip › Figure 8/Figure 8D.pdf]

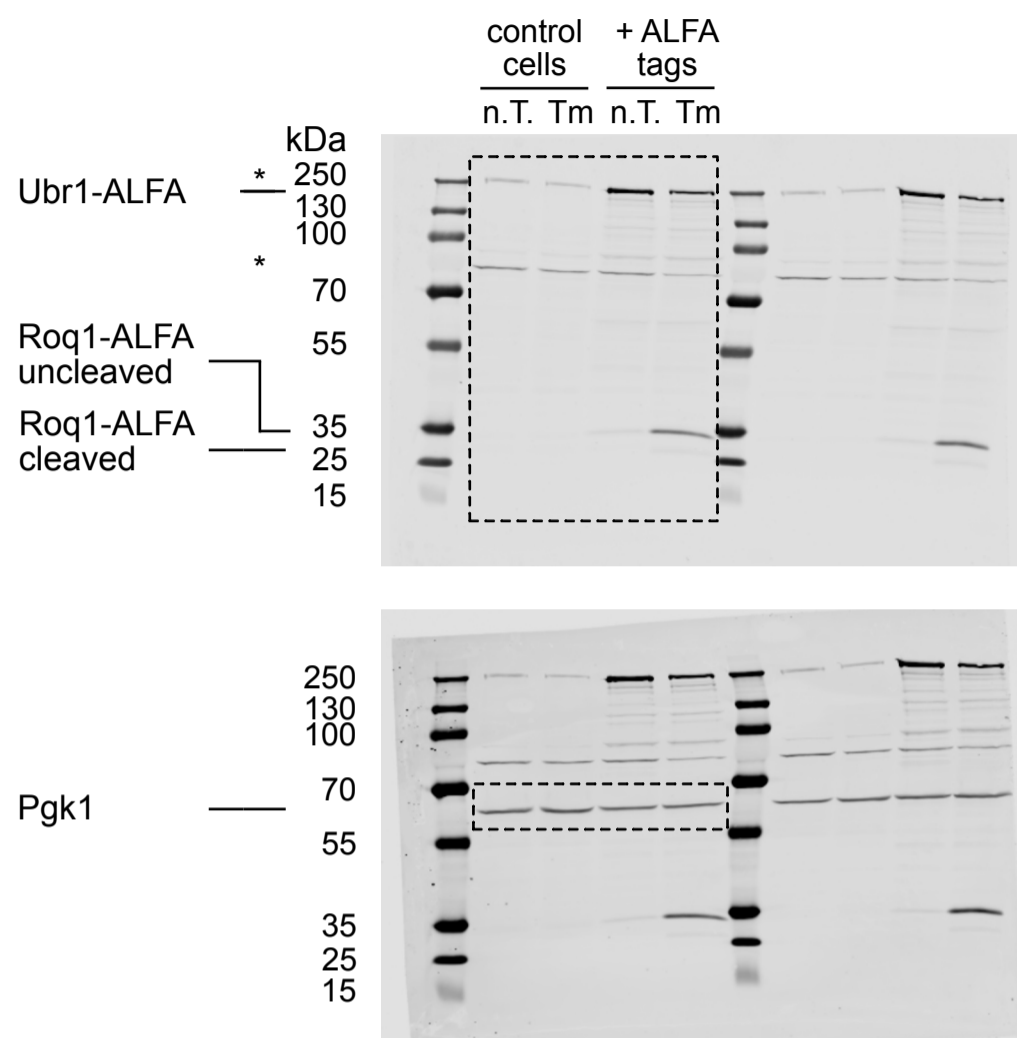

The boxed area was used for the final figure.

Supplement: Supplementary file 11 — Figure EV1 Source Data [file 44318_2025_375_MOESM11_ESM.zip › Figure EV1/Figure EV1G.pdf]

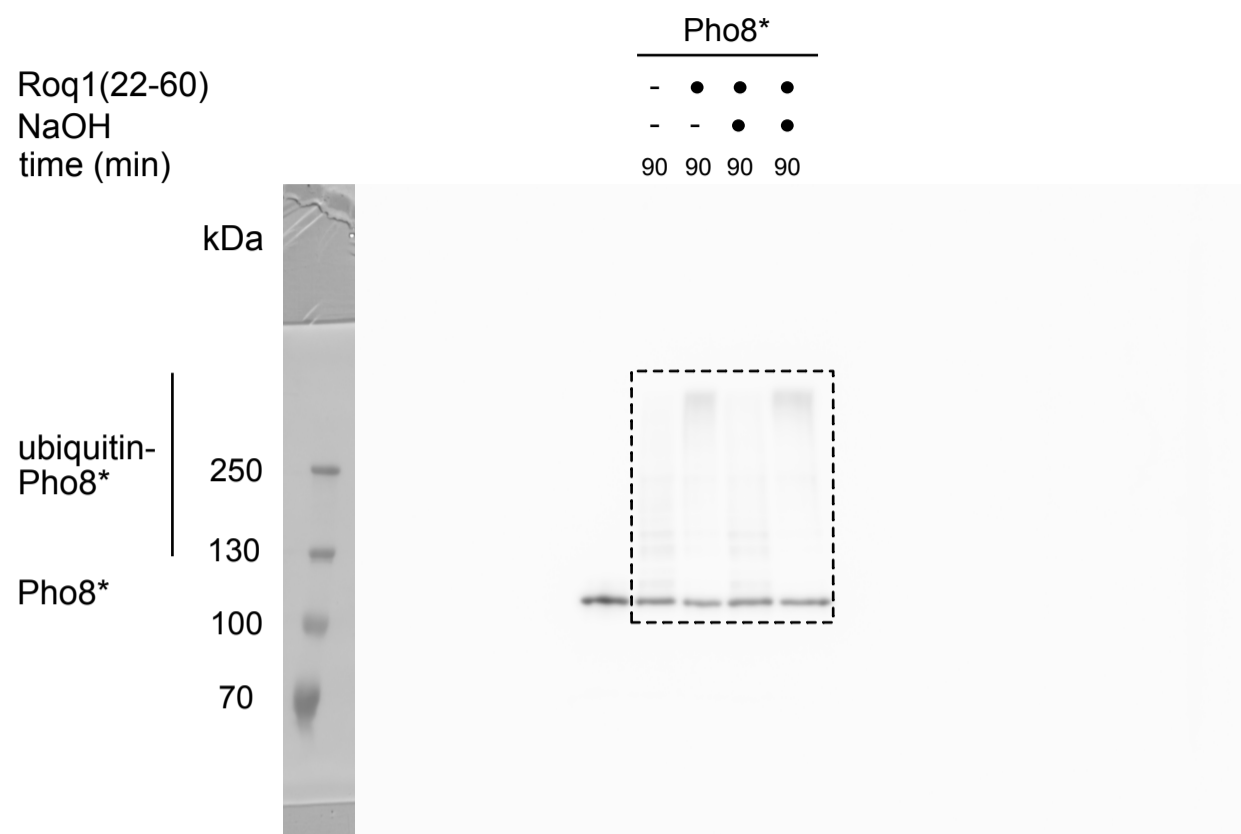

The boxed area was used for the final figure.

Supplement: Supplementary file 11 — Figure EV1 Source Data [file 44318_2025_375_MOESM11_ESM.zip › Figure EV1/Figure EV1E.pdf]

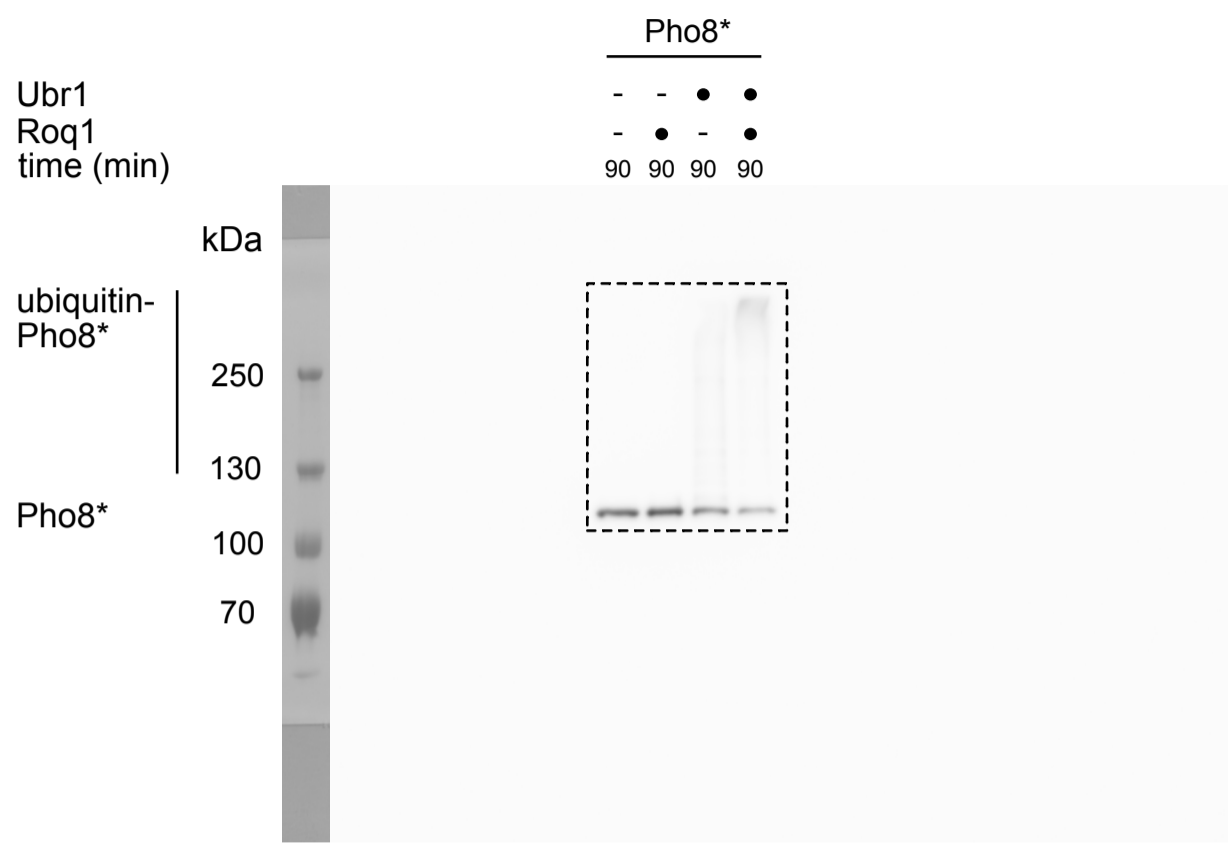

The boxed area was used for the final figure.

Supplement: Supplementary file 11 — Figure EV1 Source Data [file 44318_2025_375_MOESM11_ESM.zip › Figure EV1/Figure EV1D.pdf]

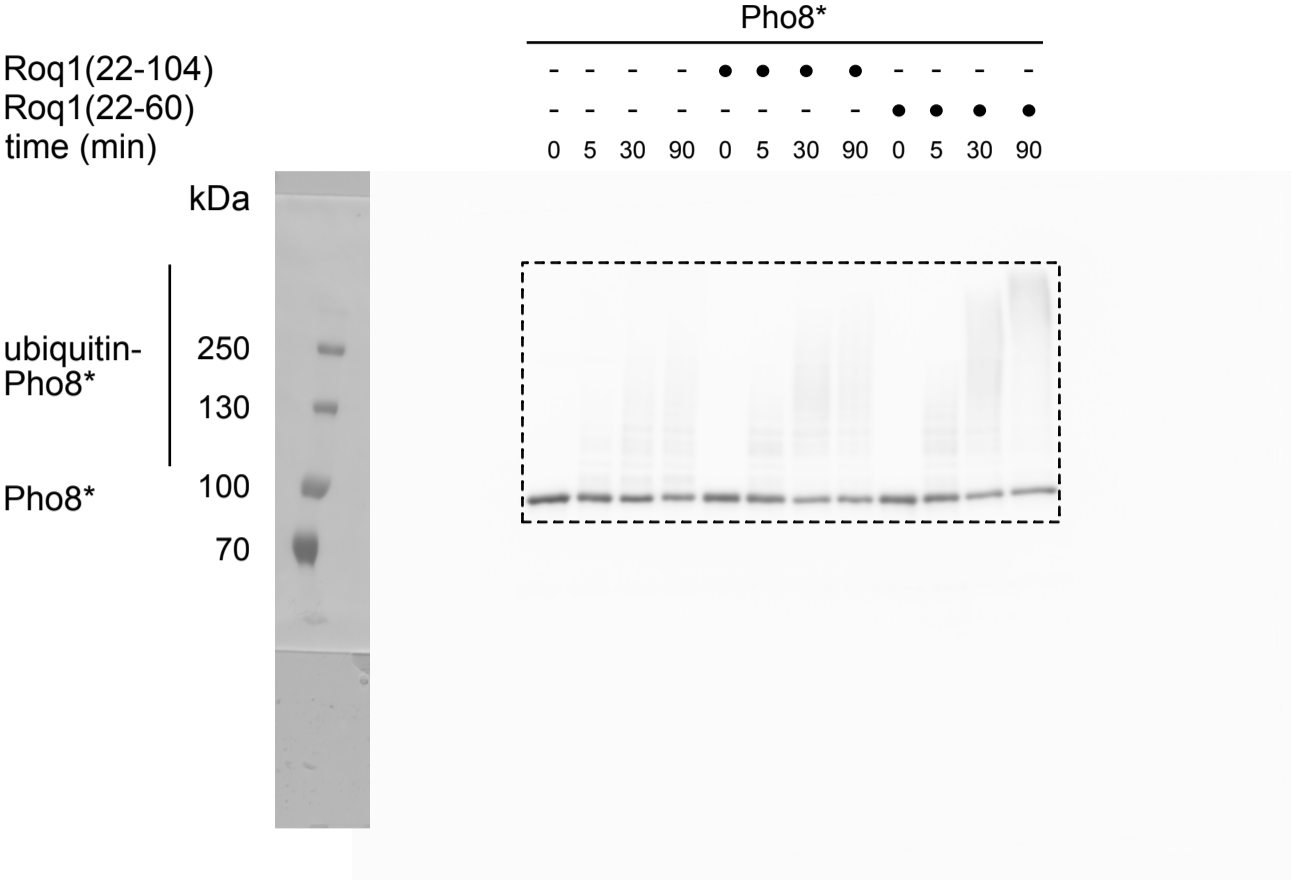

Supplement: Supplementary file 11 — Figure EV1 Source Data [file 44318_2025_375_MOESM11_ESM.zip › Figure EV1/Figure EV1H.pdf]

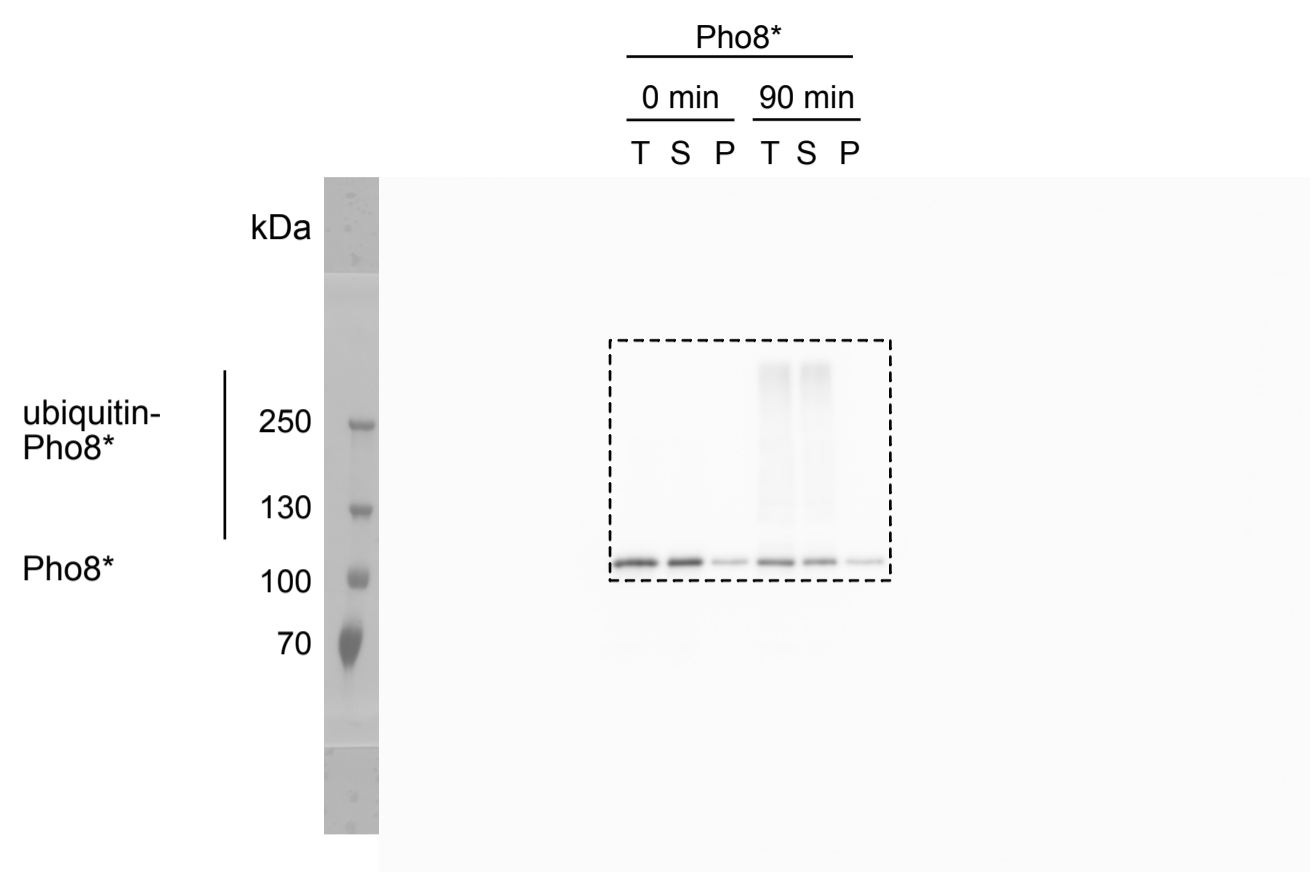

Supplement: Supplementary file 11 — Figure EV1 Source Data [file 44318_2025_375_MOESM11_ESM.zip › Figure EV1/Figure EV1C.pdf]

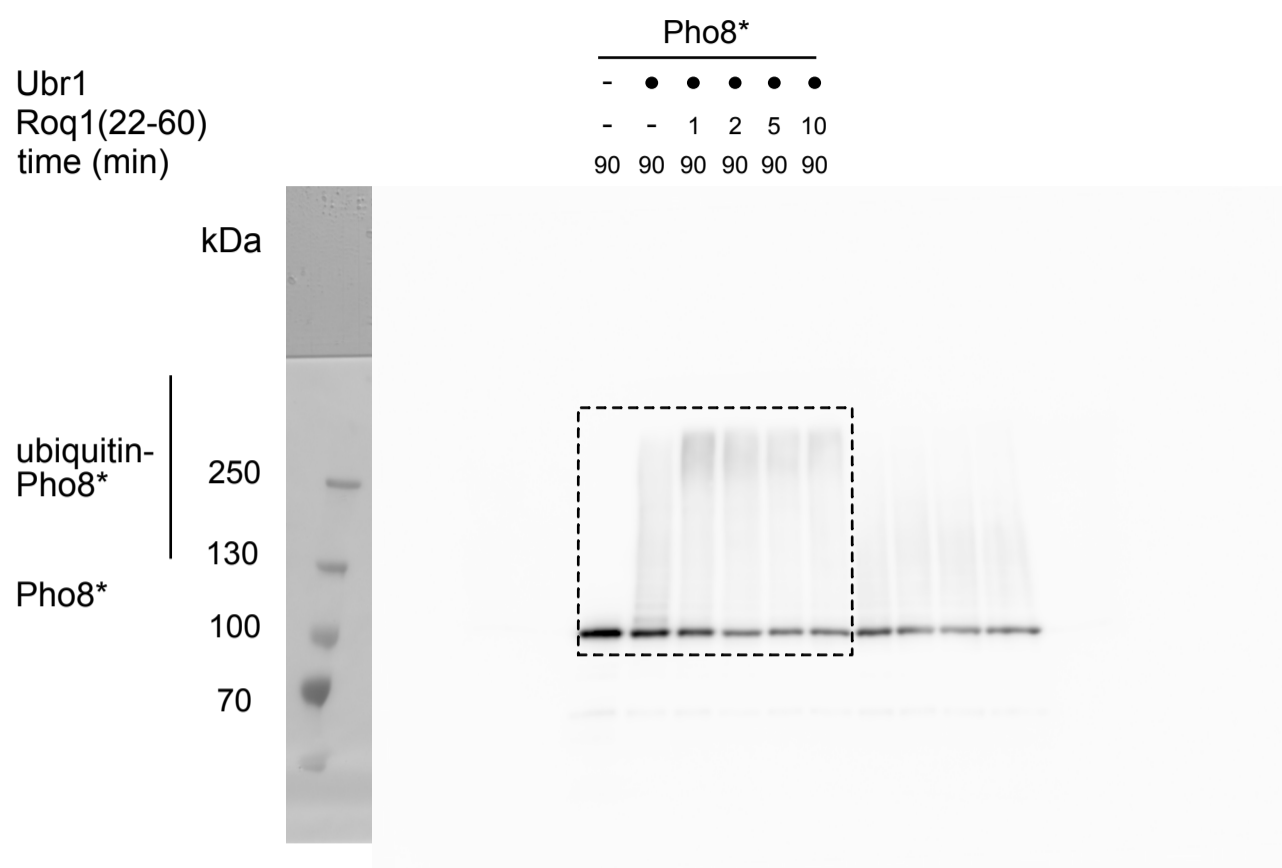

The boxed area was used for the final figure.

Supplement: Supplementary file 11 — Figure EV1 Source Data [file 44318_2025_375_MOESM11_ESM.zip › Figure EV1/Figure EV1F.pdf]

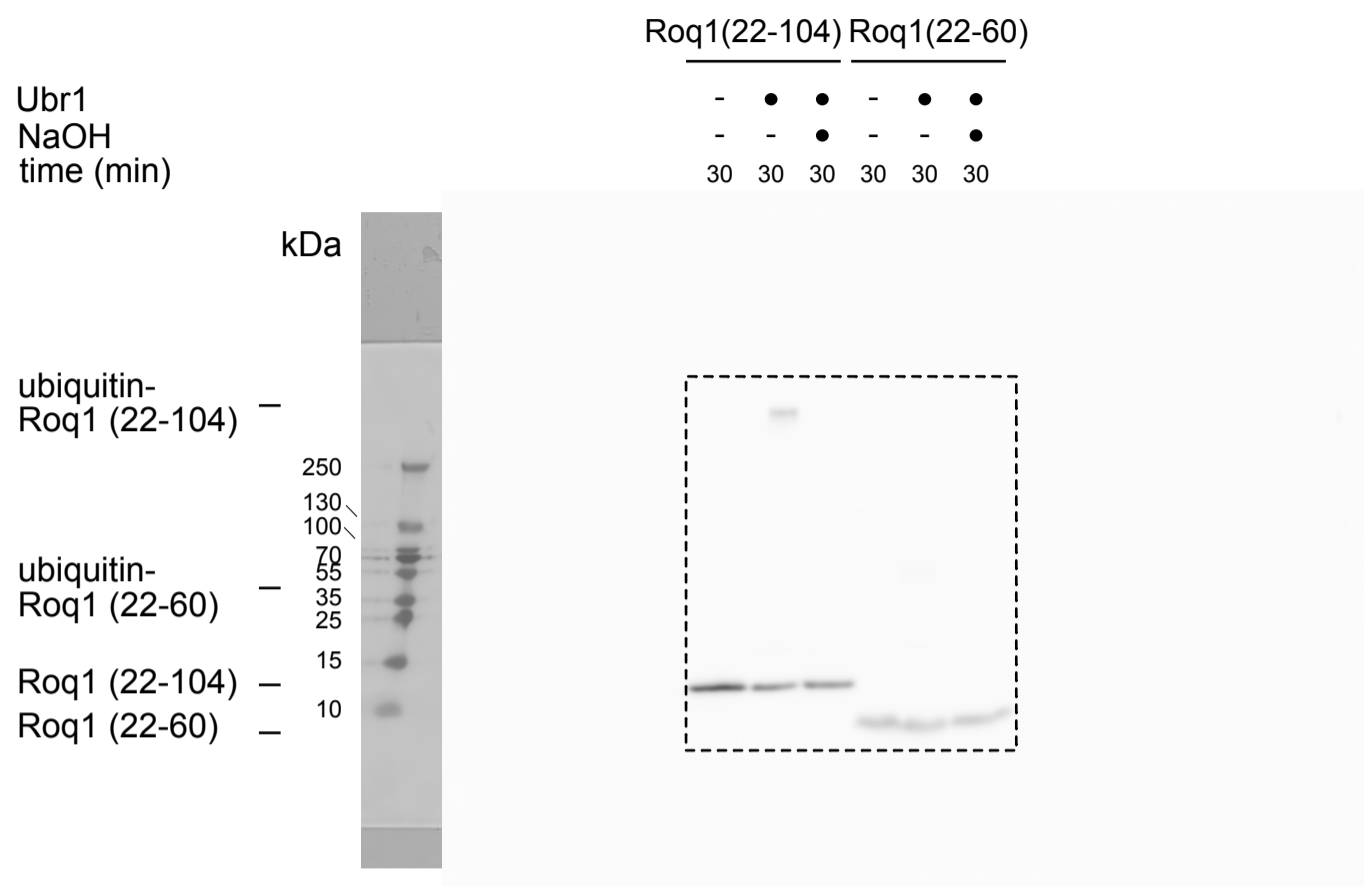

The boxed area was used for the final figure.

Supplement: Supplementary file 11 — Figure EV1 Source Data [file 44318_2025_375_MOESM11_ESM.zip › Figure EV1/Figure EV1A.pdf]

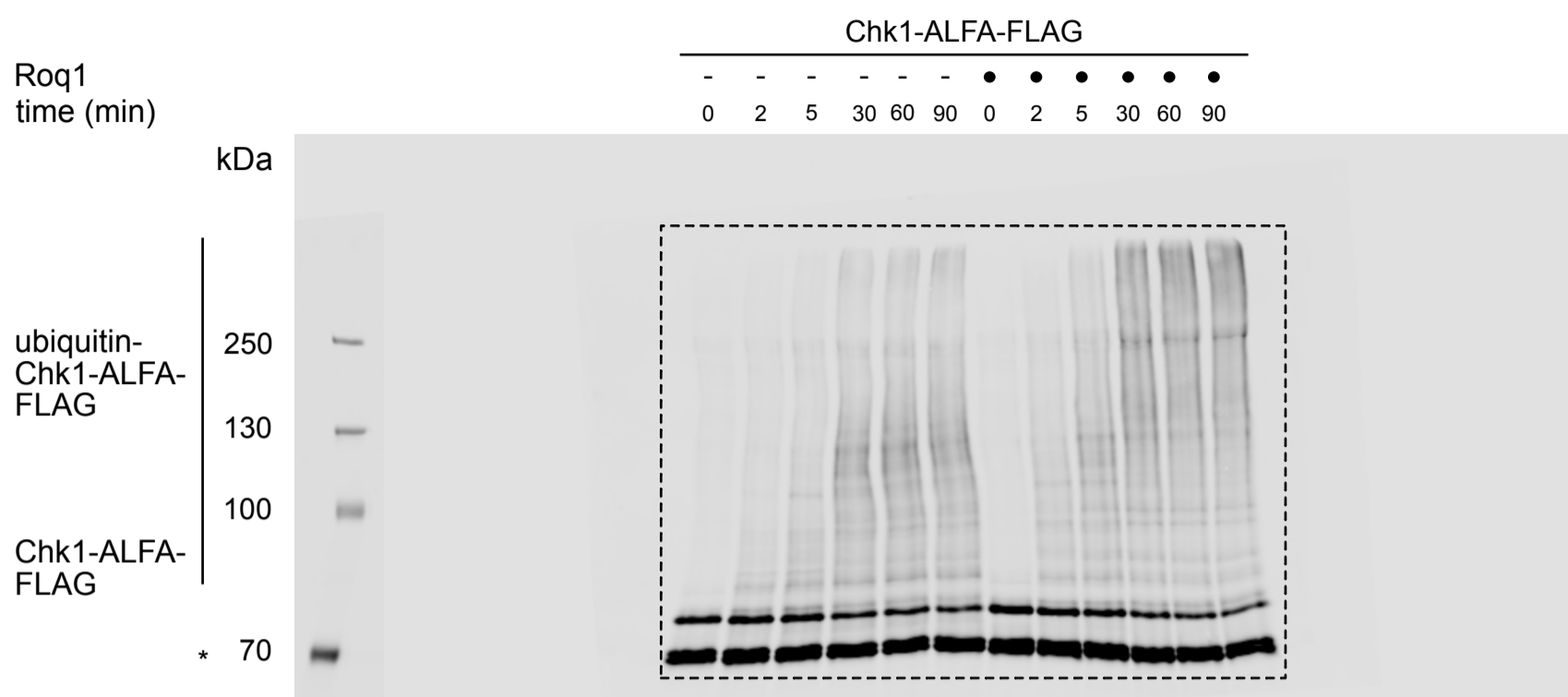

The boxed area was used for the final figure.

Supplement: Supplementary file 12 — Figure EV2 Source Data [file 44318_2025_375_MOESM12_ESM.zip › Figure EV2/Figure EV2C.pdf]

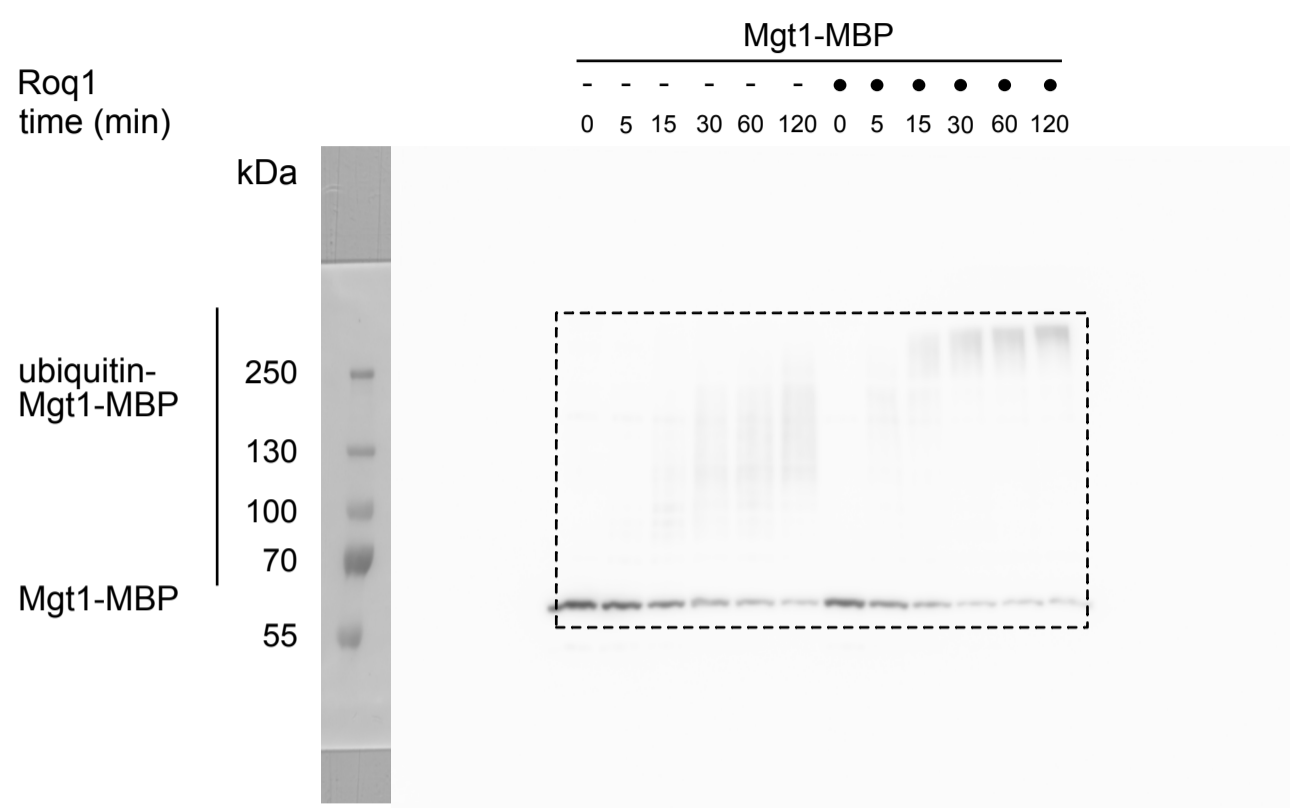

The boxed area was used for the final figure.

Supplement: Supplementary file 12 — Figure EV2 Source Data [file 44318_2025_375_MOESM12_ESM.zip › Figure EV2/Figure EV2B.pdf]

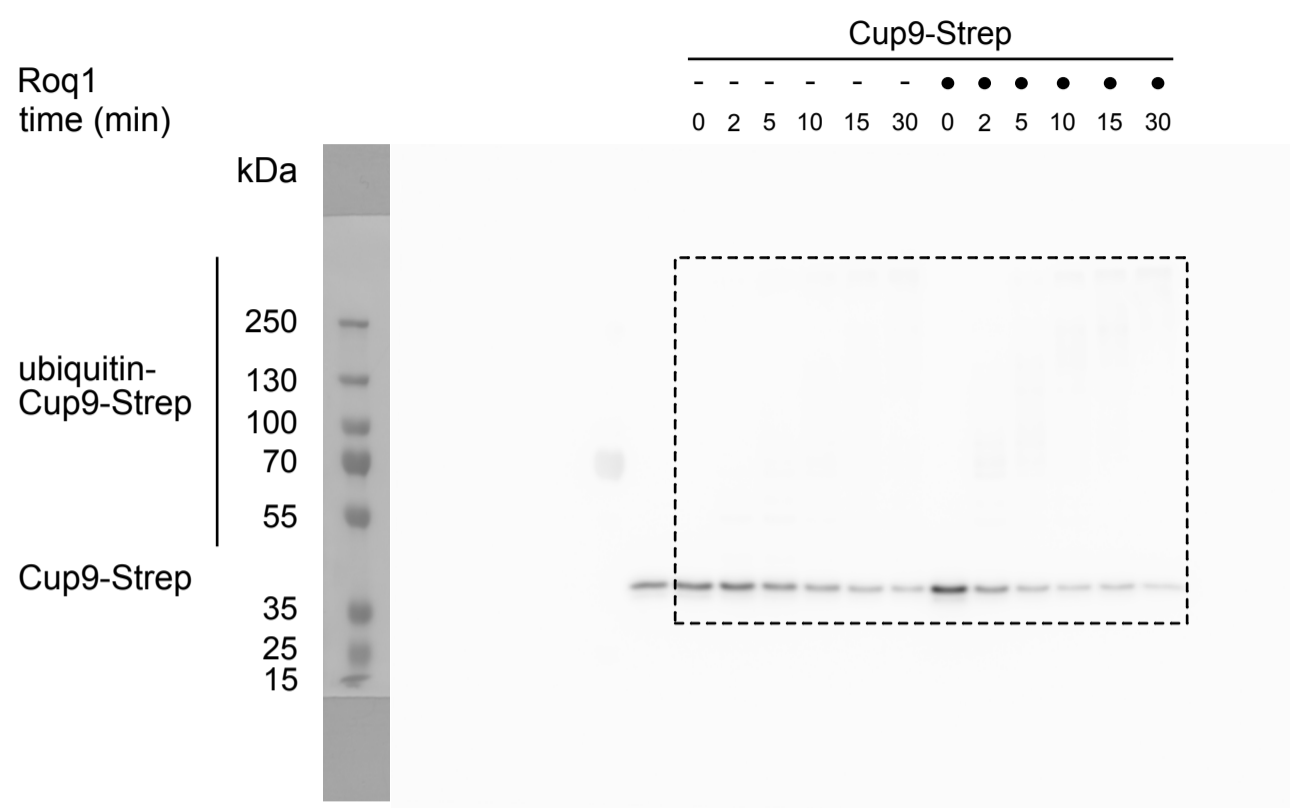

The boxed area was used for the final figure.

Supplement: Supplementary file 12 — Figure EV2 Source Data [file 44318_2025_375_MOESM12_ESM.zip › Figure EV2/Figure EV2A.pdf]

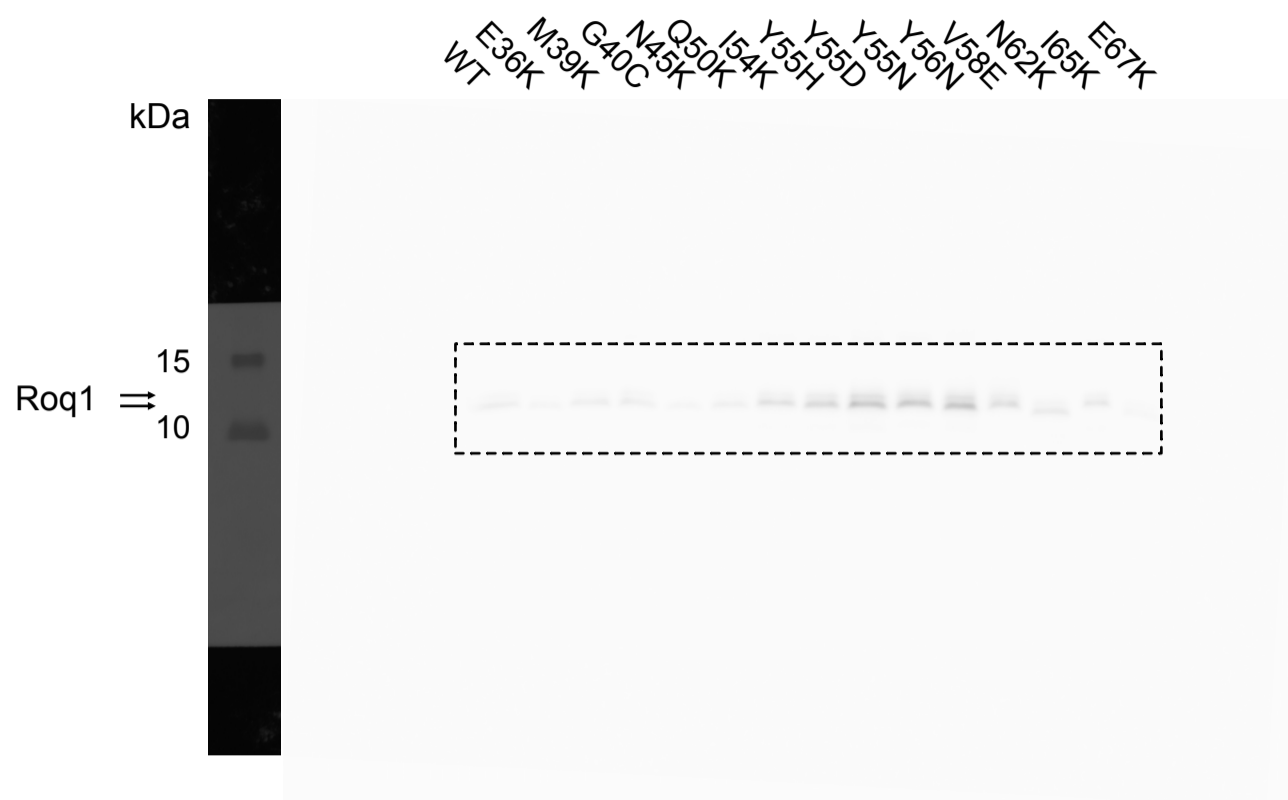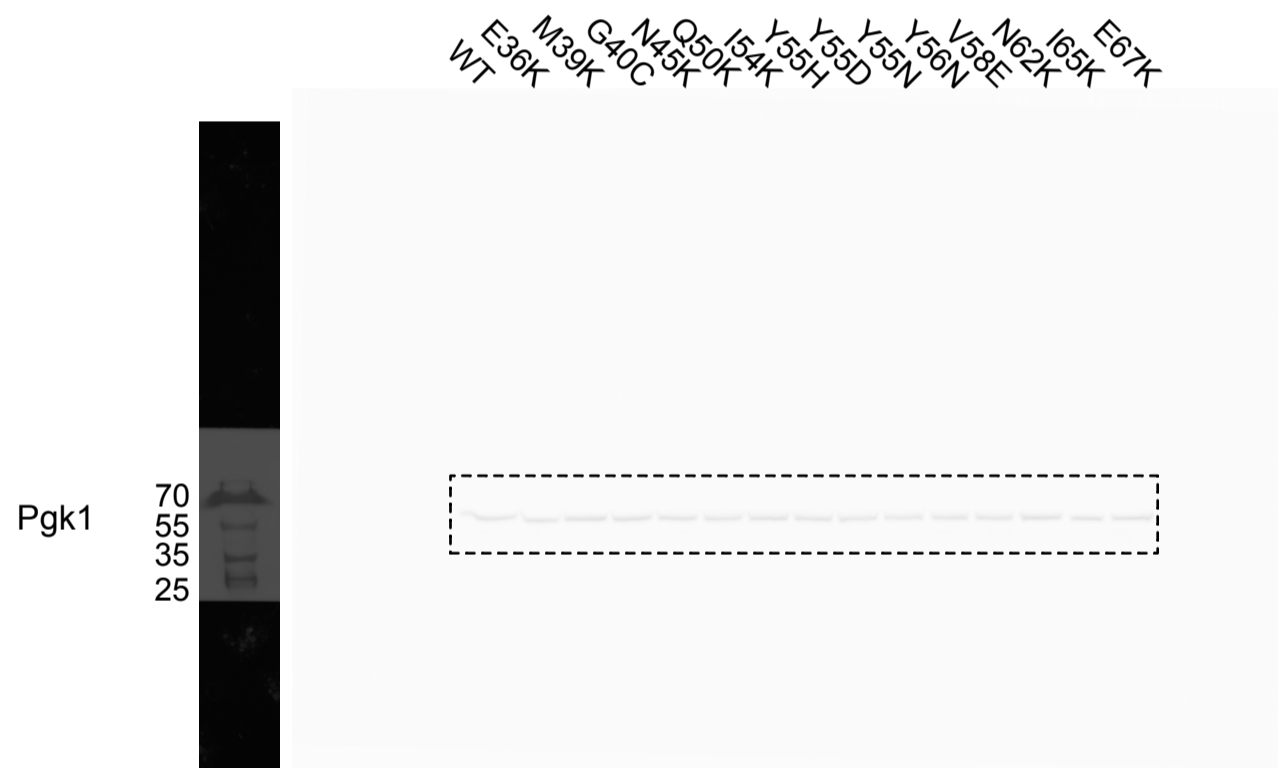

The boxed area was used for the final figure.

Supplement: Supplementary file 14 — Figure EV4 Source Data [file 44318_2025_375_MOESM14_ESM.zip › Figure EV4/Figure EV4D.pdf]

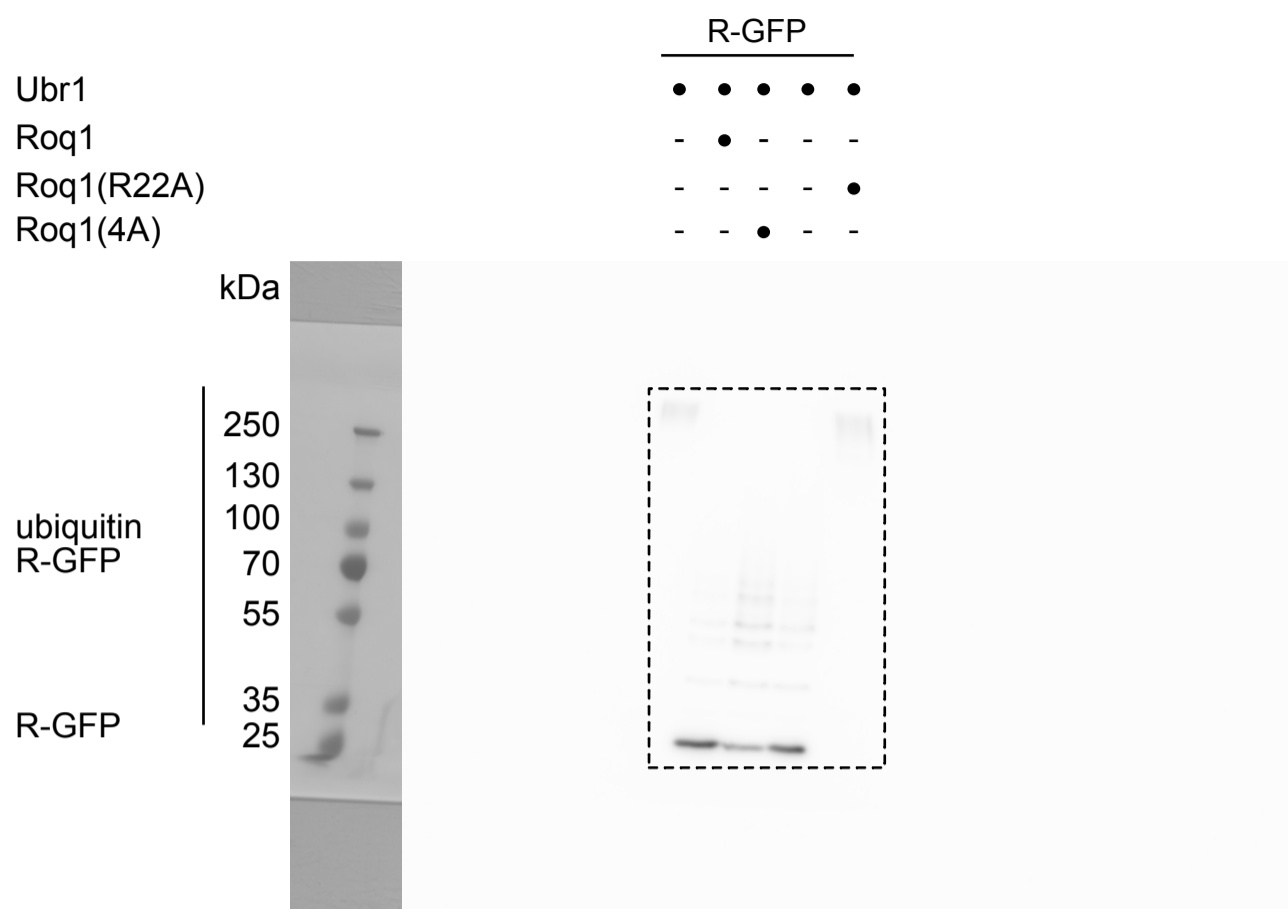

The boxed area was used for the final figure.

Supplement: Supplementary file 15 — Figure EV5 Source Data [file 44318_2025_375_MOESM15_ESM.zip › Figure EV5/Figure EV5C.pdf]

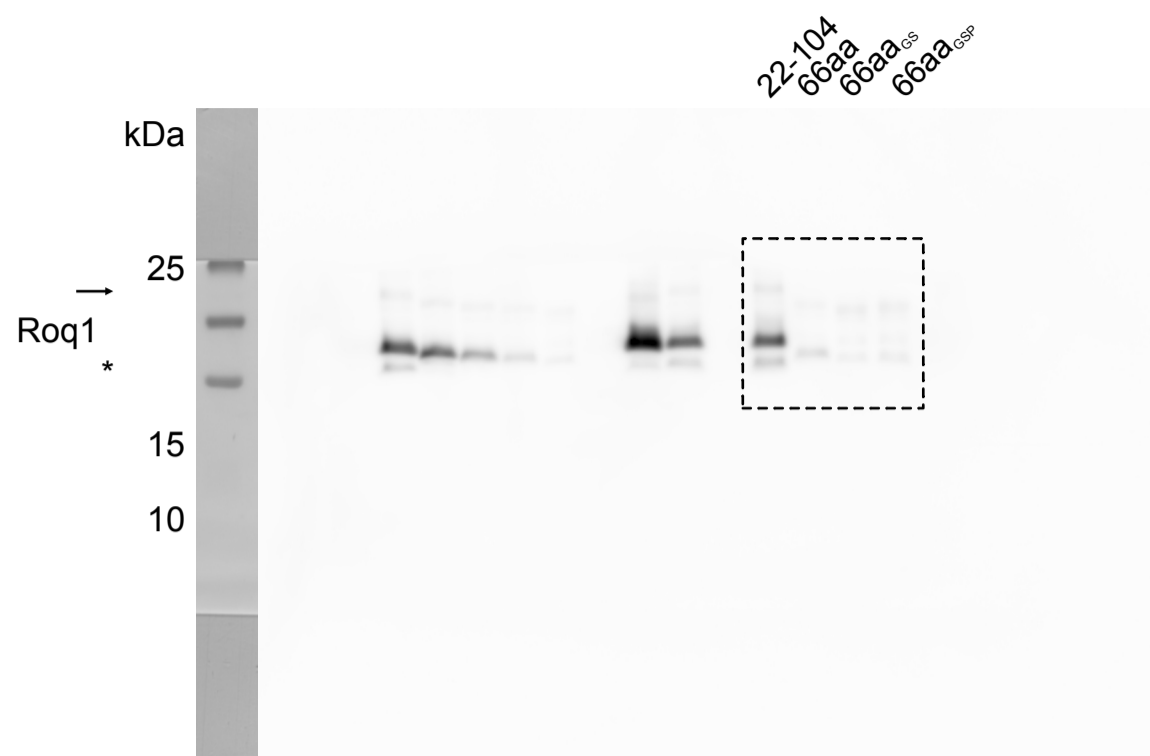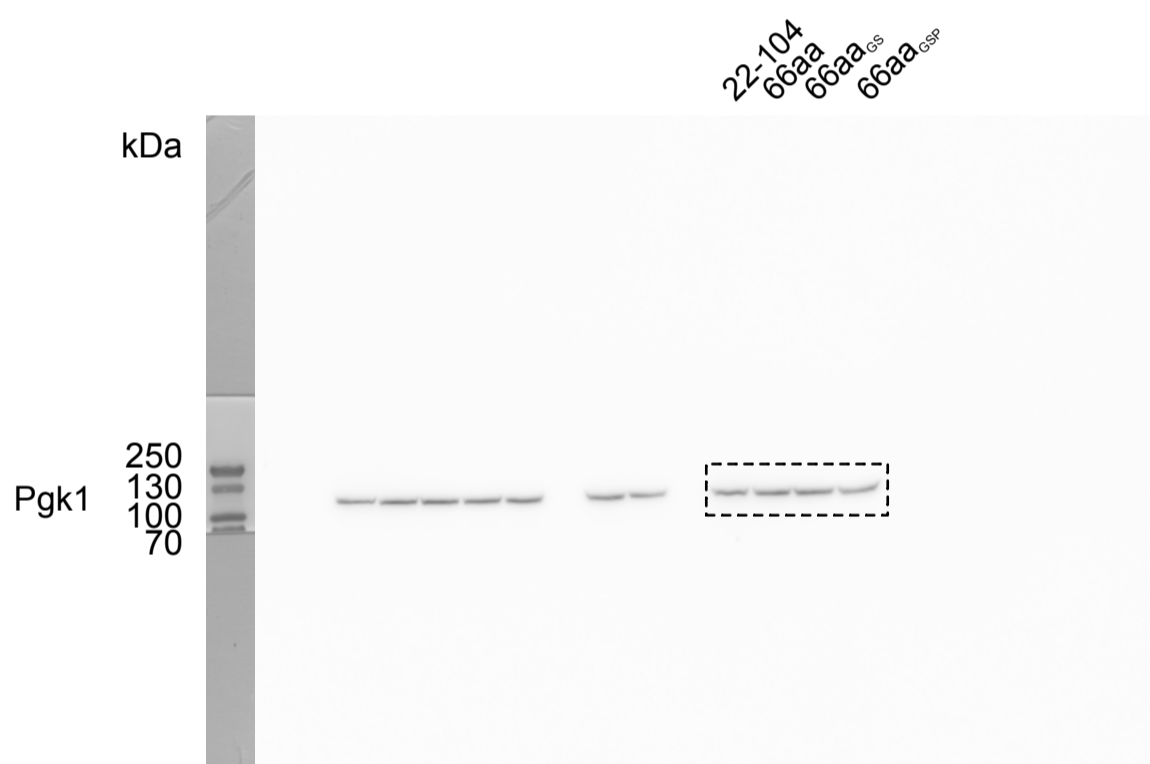

The boxed area was used for the final figure.

Supplement: Supplementary file 15 — Figure EV5 Source Data [file 44318_2025_375_MOESM15_ESM.zip › Figure EV5/Figure EV5G.pdf]

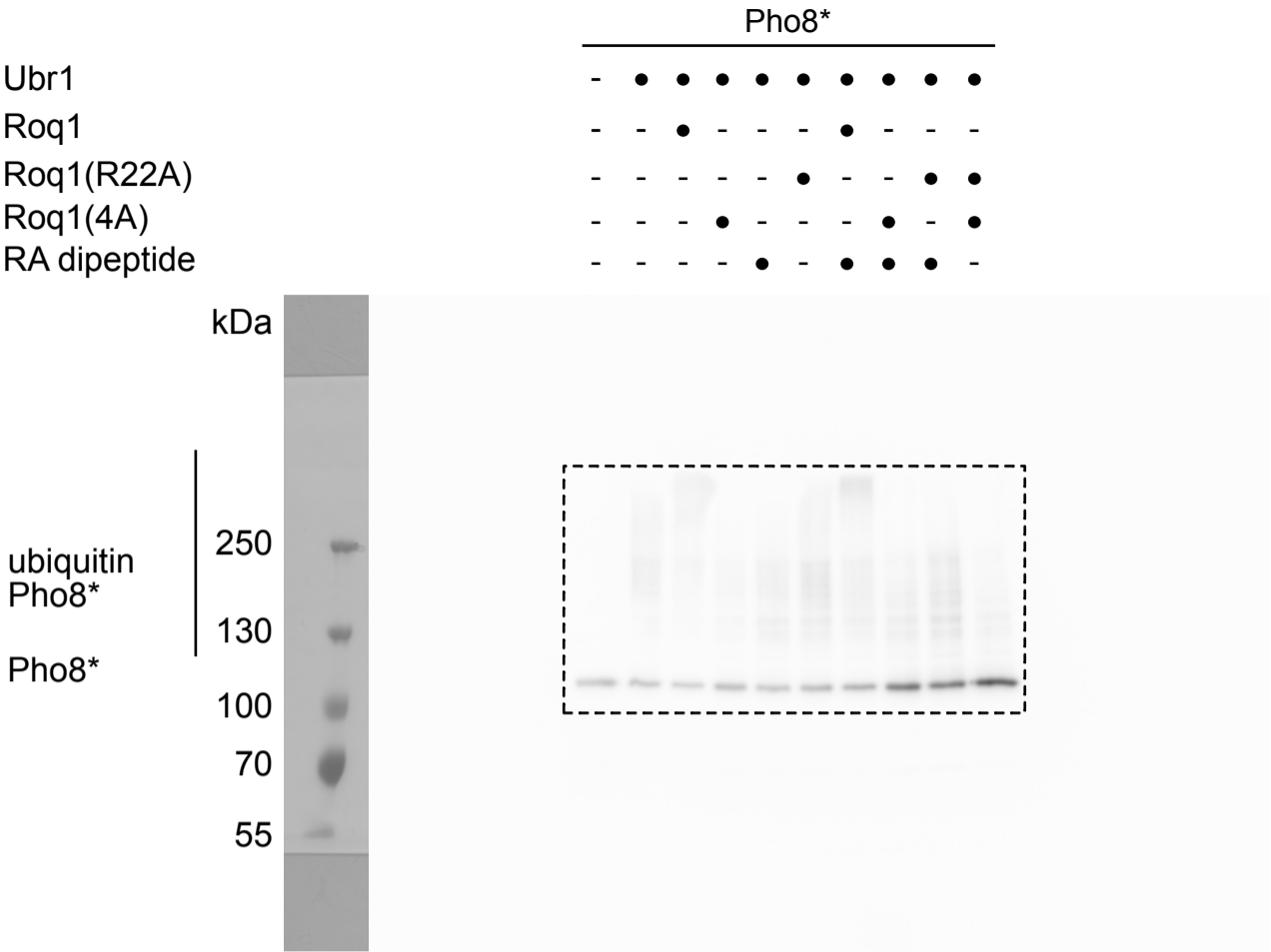

The boxed area was used for the final figure.

Supplement: Supplementary file 15 — Figure EV5 Source Data [file 44318_2025_375_MOESM15_ESM.zip › Figure EV5/Figure EV5D.pdf]

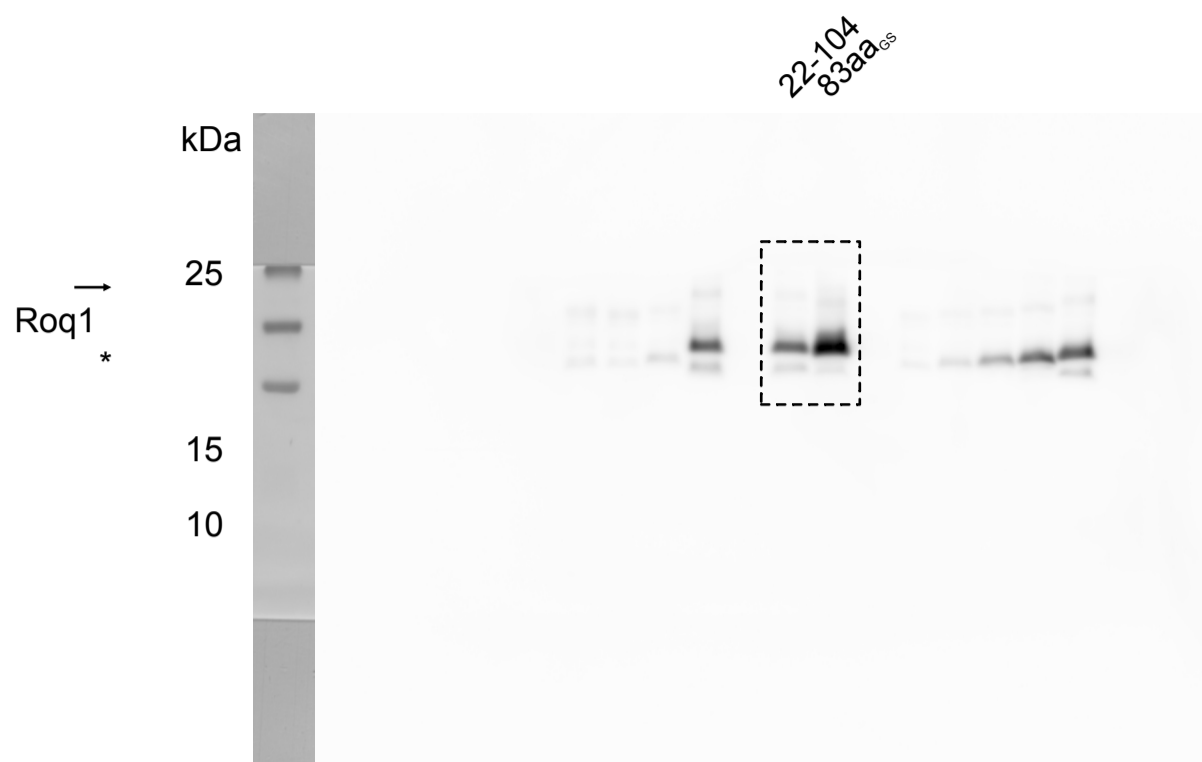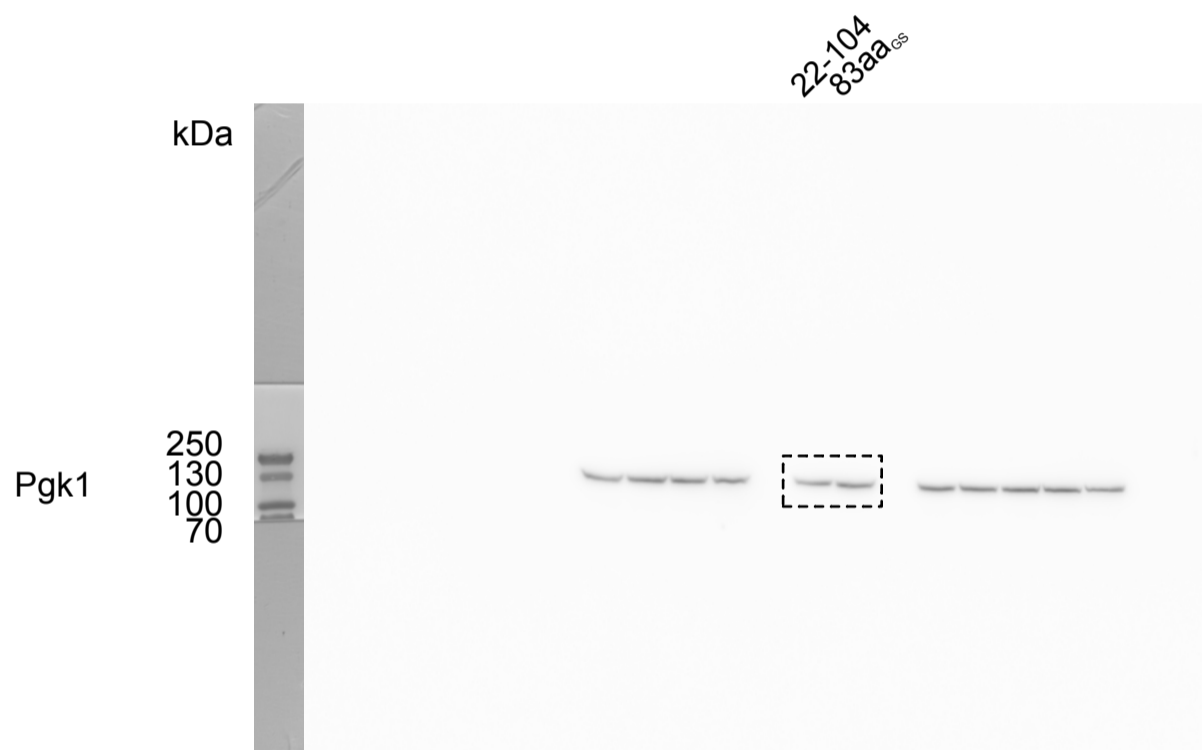

The boxed area was used for the final figure.

Supplement: Supplementary file 15 — Figure EV5 Source Data [file 44318_2025_375_MOESM15_ESM.zip › Figure EV5/Figure EV5E.pdf]

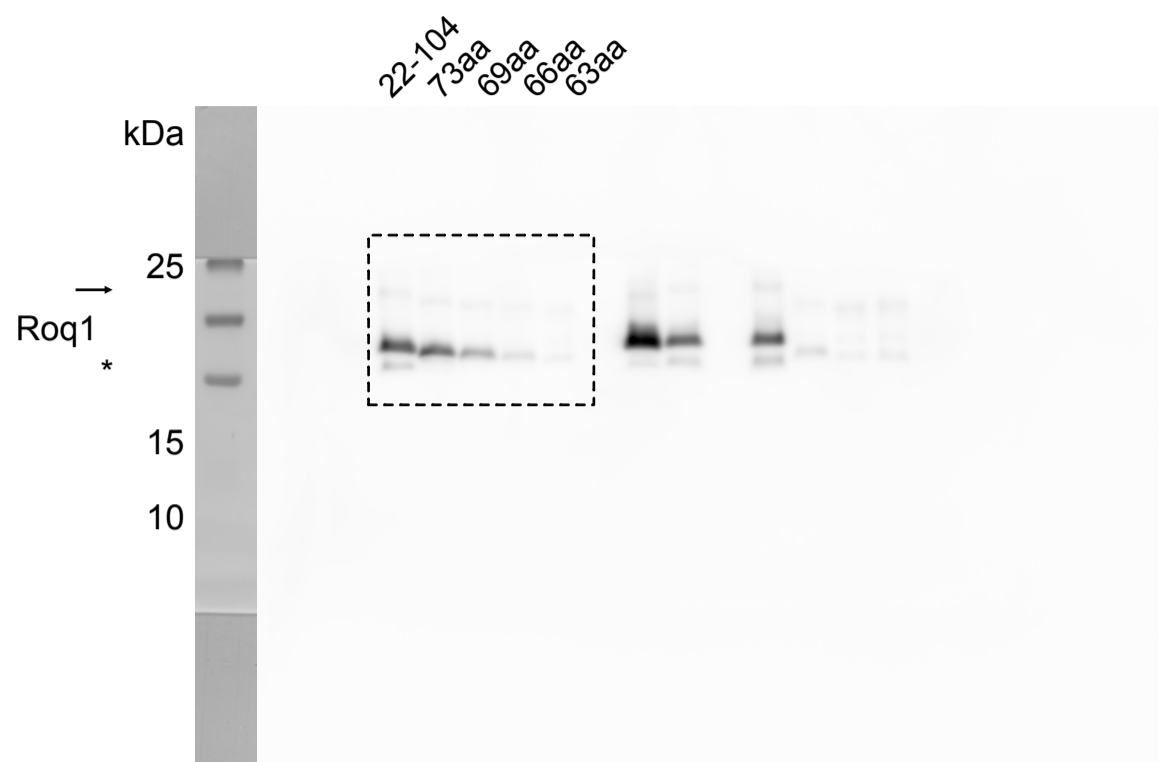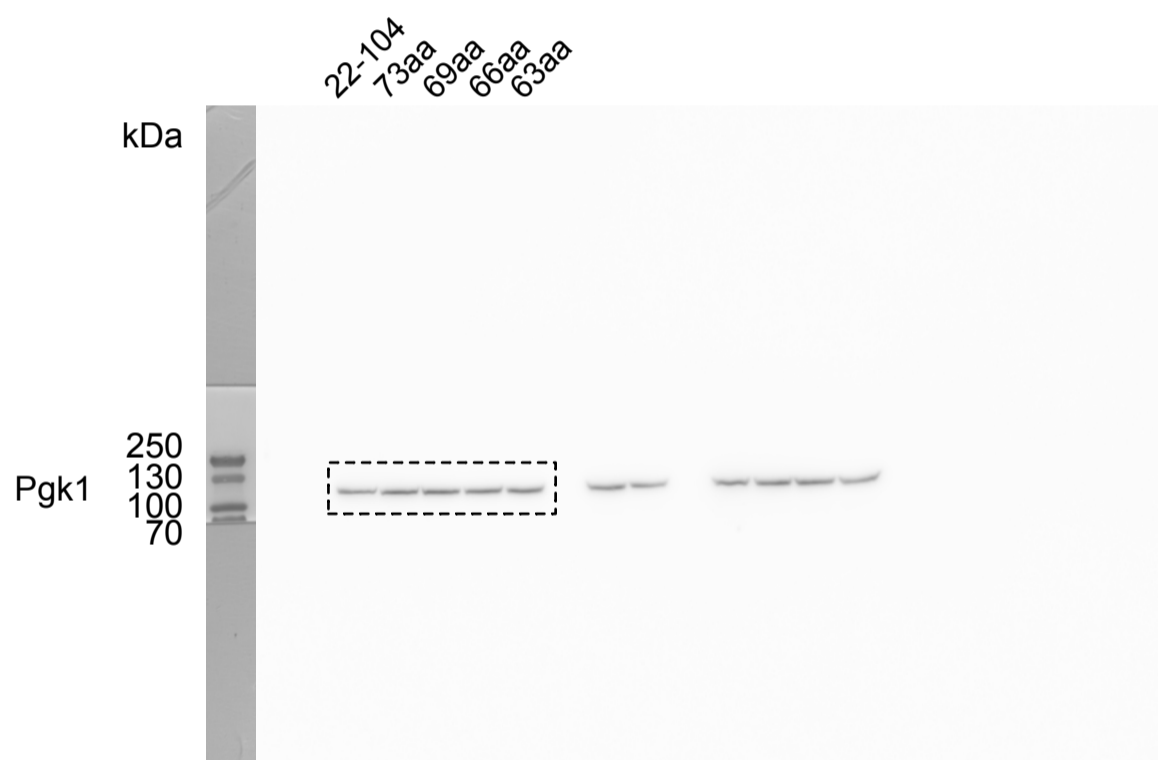

The boxed area was used for the final figure.

Supplement: Supplementary file 15 — Figure EV5 Source Data [file 44318_2025_375_MOESM15_ESM.zip › Figure EV5/Figure EV5F.pdf]

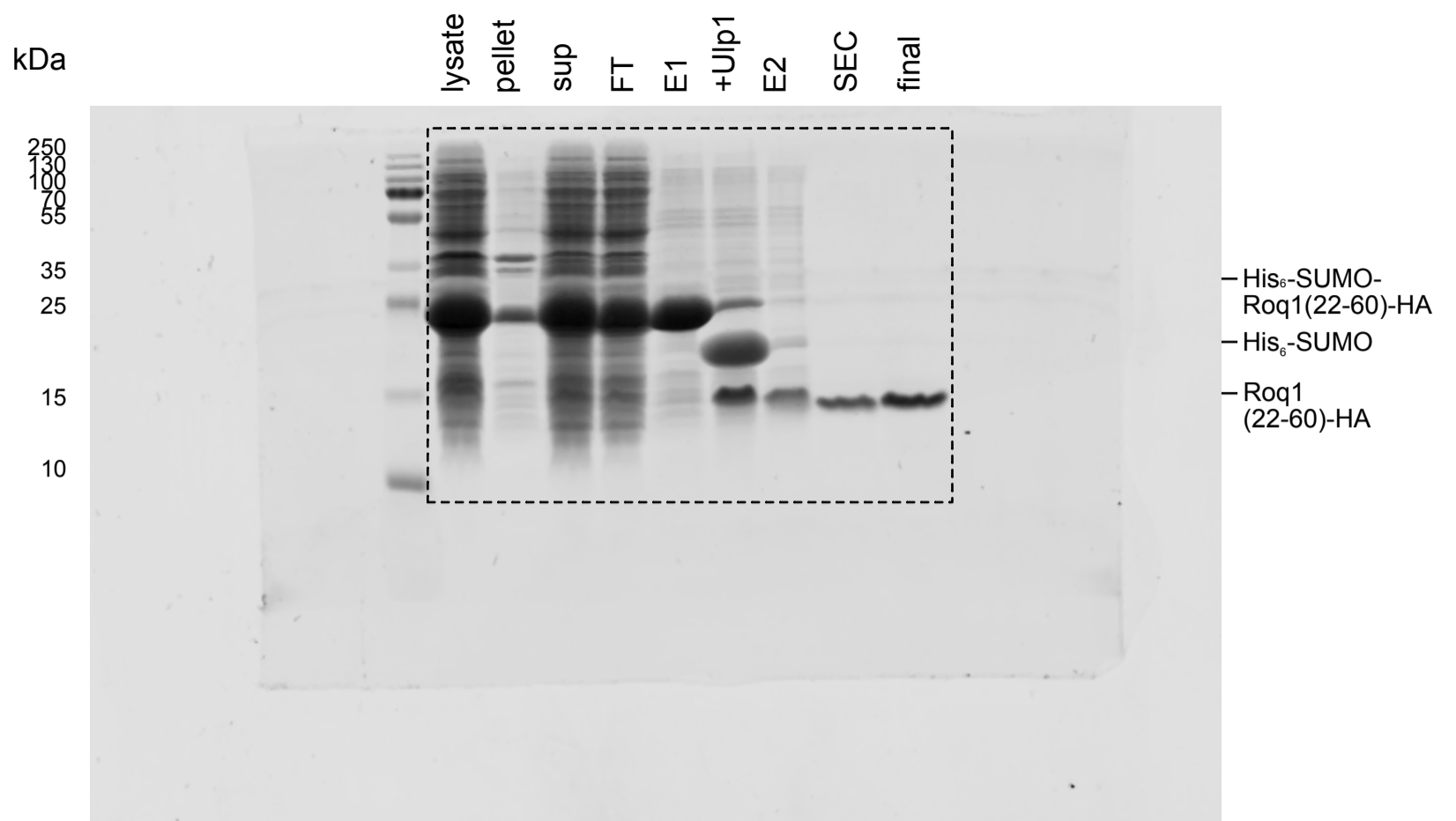

The boxed area was used for the final figure.

Supplement: Supplementary file 16 — Appendix Figure S1 Source Data [file 44318_2025_375_MOESM16_ESM.zip › Appendix Figure S1/Appendix Figure S1C.pdf]

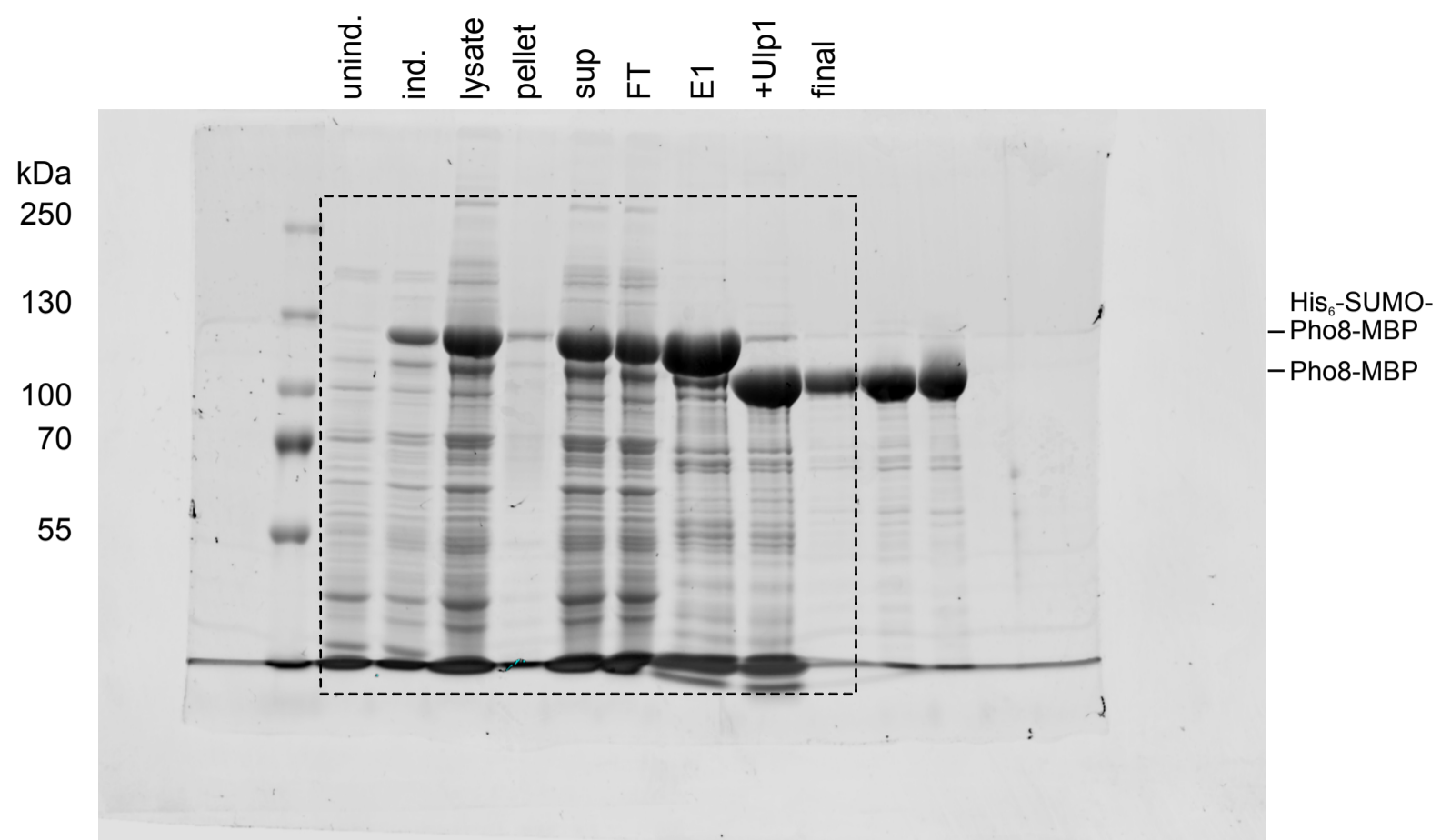

The boxed area was used for the final figure.

Supplement: Supplementary file 16 — Appendix Figure S1 Source Data [file 44318_2025_375_MOESM16_ESM.zip › Appendix Figure S1/Appendix Figure S1G.pdf]

purified Rad6

final

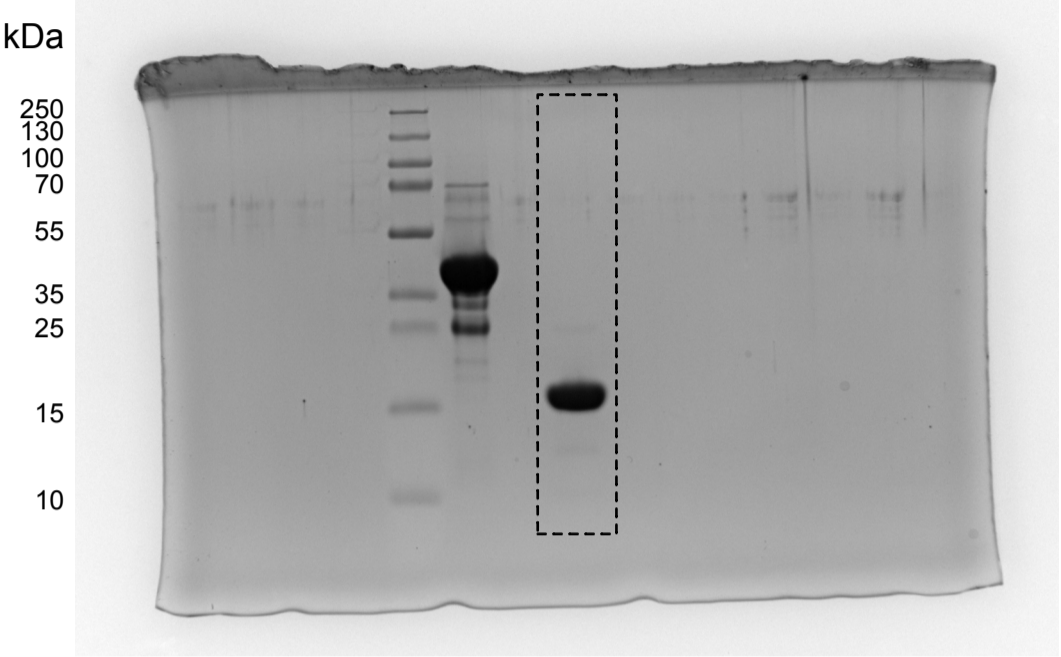

The boxed area was used for the final figure.

Supplement: Supplementary file 16 — Appendix Figure S1 Source Data [file 44318_2025_375_MOESM16_ESM.zip › Appendix Figure S1/Appendix Figure S1D.pdf]

**purified ubiquitin**

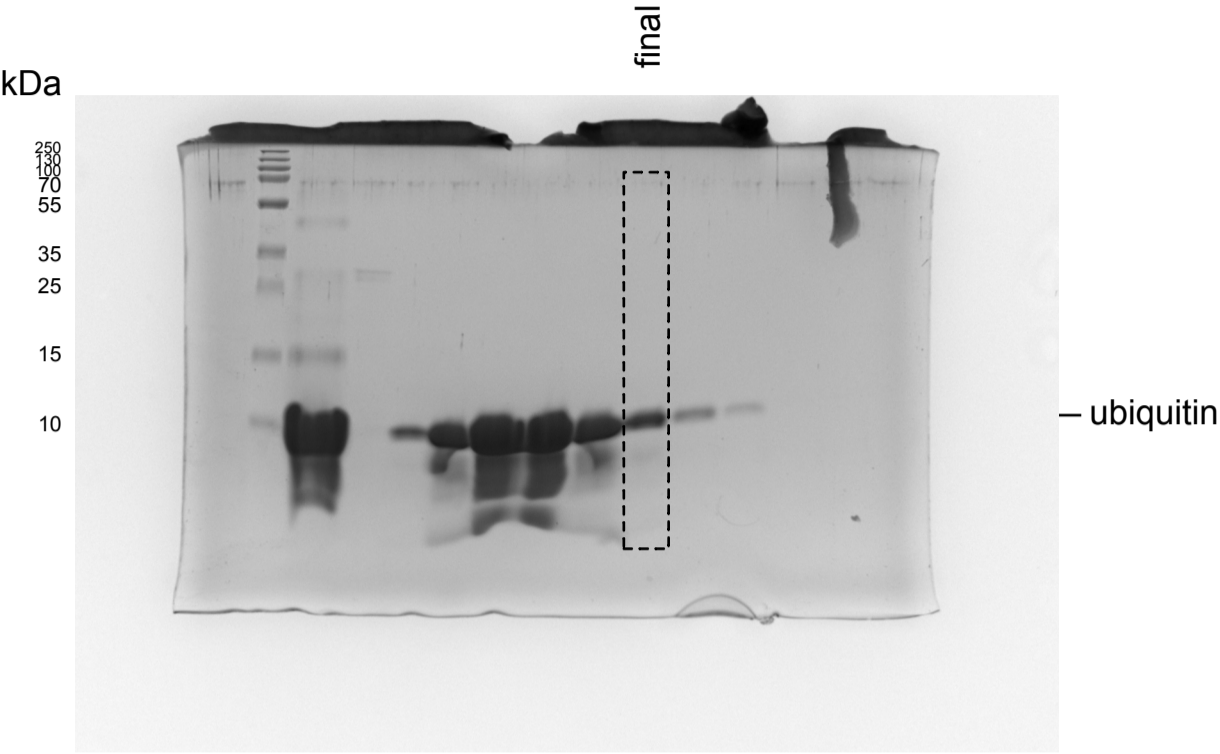

The boxed area was used for the final figure.

Supplement: Supplementary file 16 — Appendix Figure S1 Source Data [file 44318_2025_375_MOESM16_ESM.zip › Appendix Figure S1/Appendix Figure S1K.pdf]

**purified Cup9**

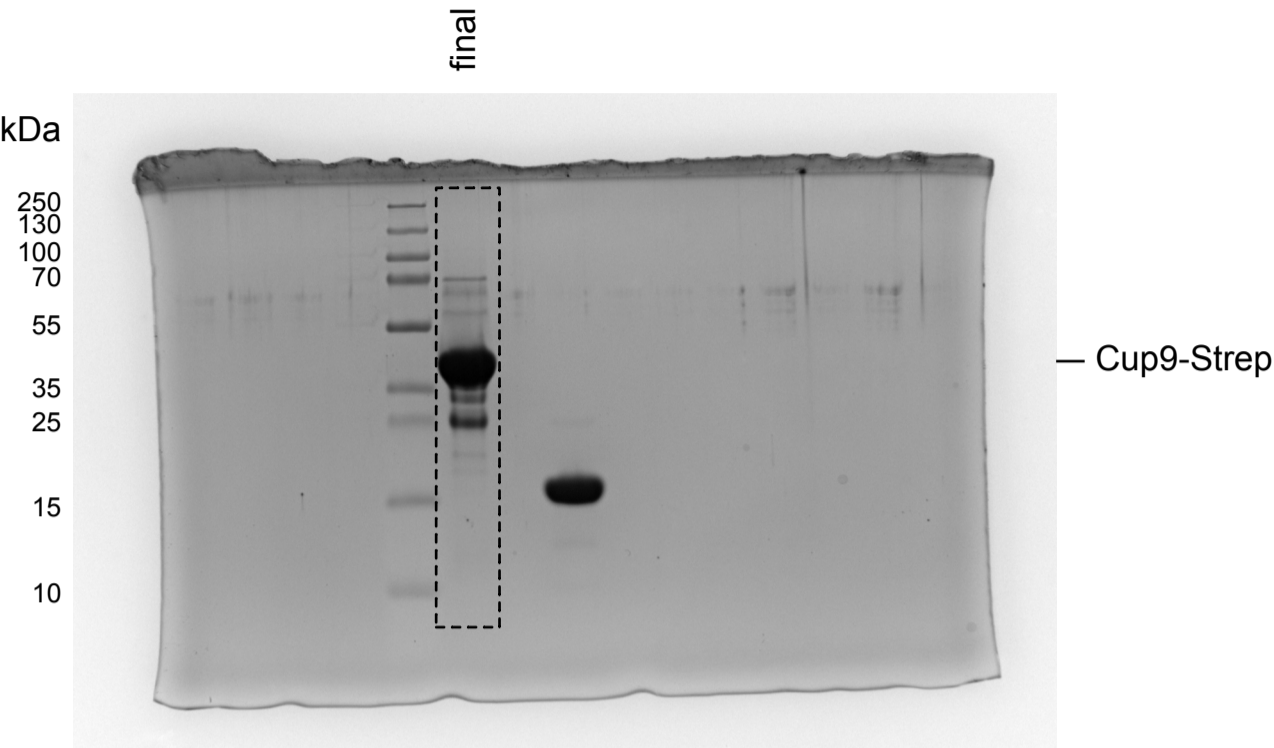

The boxed area was used for the final figure.

Supplement: Supplementary file 16 — Appendix Figure S1 Source Data [file 44318_2025_375_MOESM16_ESM.zip › Appendix Figure S1/Appendix Figure S1I.pdf]

# **purification F-GFP**

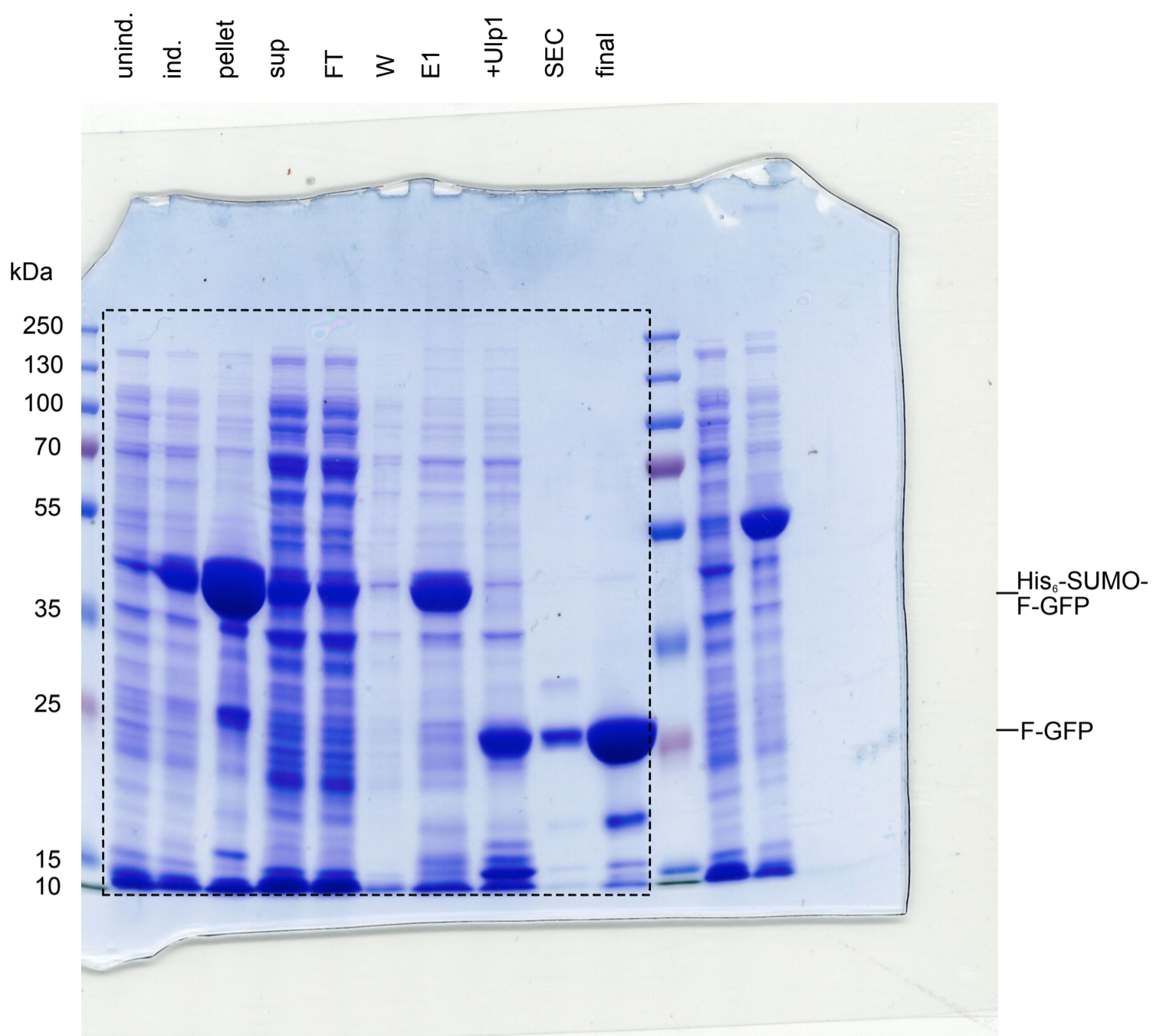

The boxed area was used for the final figure.

Supplement: Supplementary file 16 — Appendix Figure S1 Source Data [file 44318_2025_375_MOESM16_ESM.zip › Appendix Figure S1/Appendix Figure S1E.pdf]

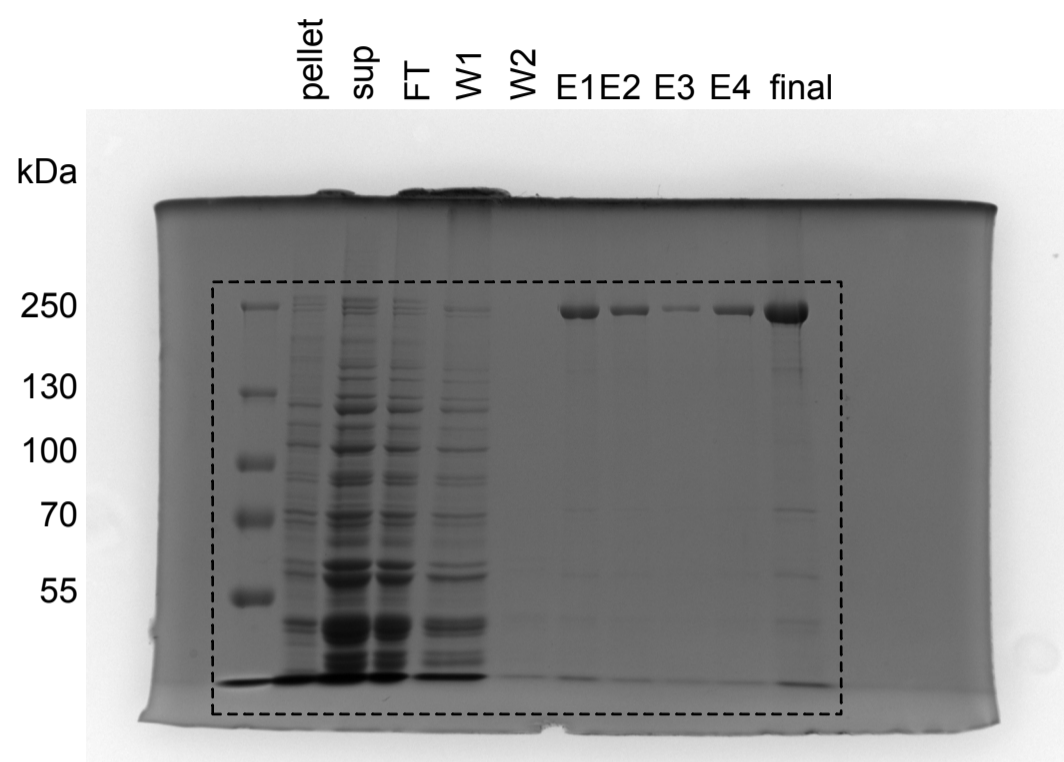

The boxed area was used for the final figure.

Supplement: Supplementary file 16 — Appendix Figure S1 Source Data [file 44318_2025_375_MOESM16_ESM.zip › Appendix Figure S1/Appendix Figure S1A.pdf]

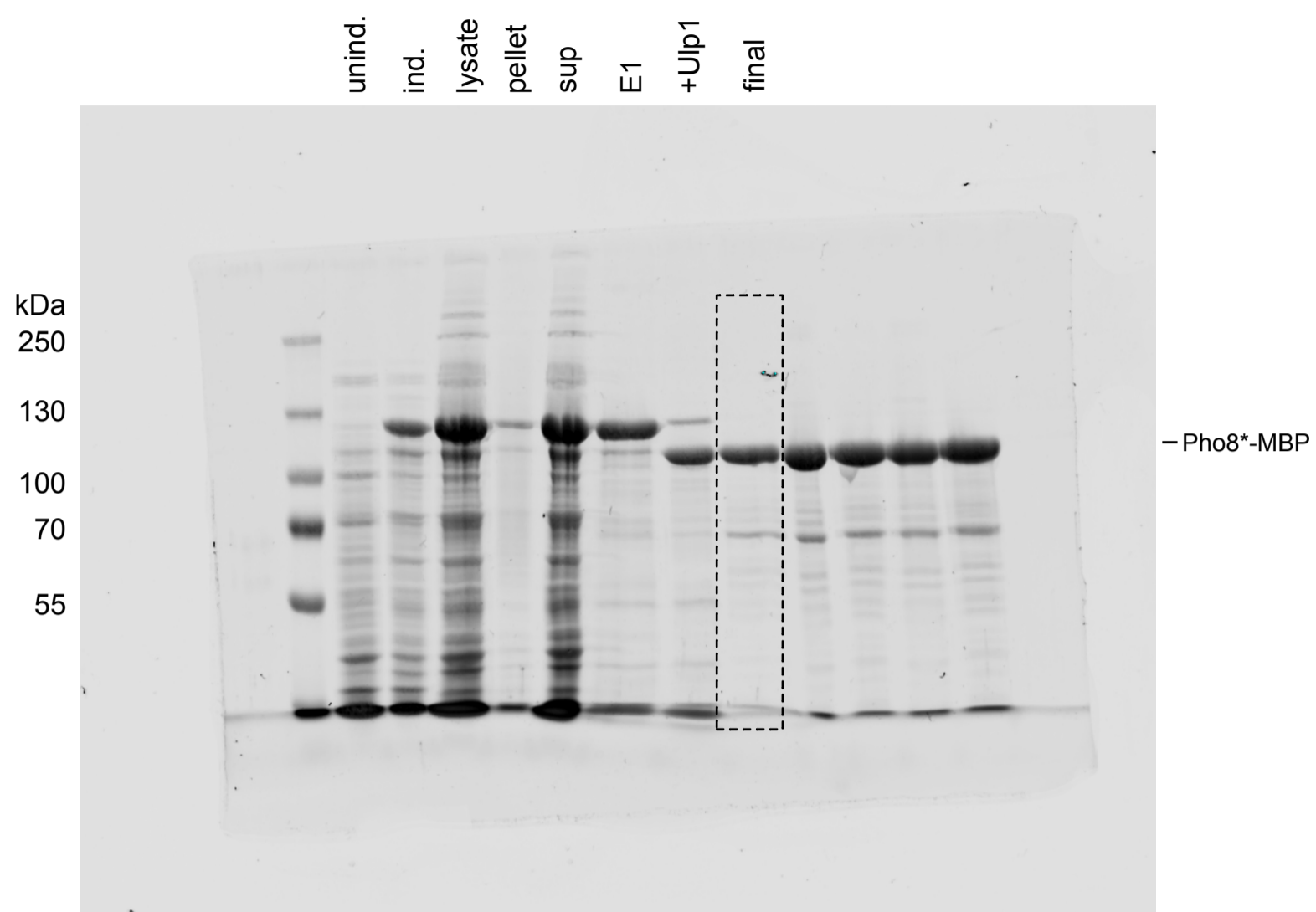

The boxed area was used for the final figure.

Supplement: Supplementary file 16 — Appendix Figure S1 Source Data [file 44318_2025_375_MOESM16_ESM.zip › Appendix Figure S1/Appendix Figure S1H.pdf]

**purified R-GFP**

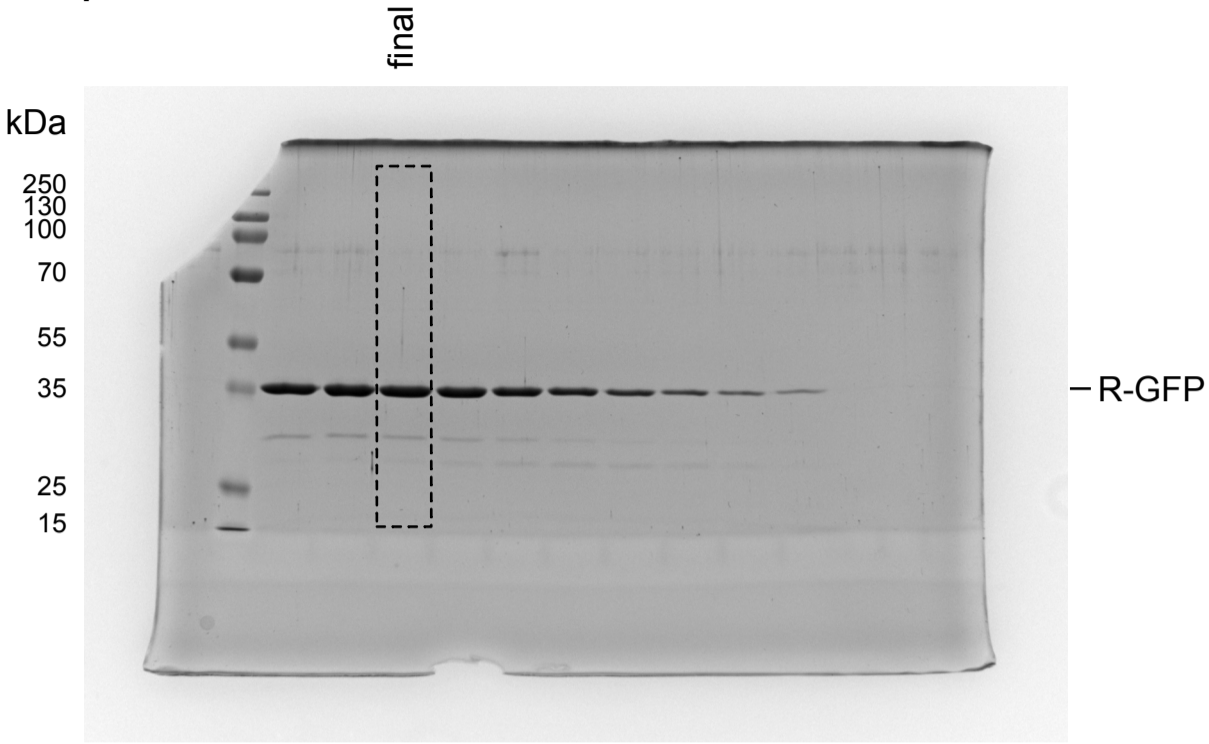

The boxed area was used for the final figure.

Supplement: Supplementary file 16 — Appendix Figure S1 Source Data [file 44318_2025_375_MOESM16_ESM.zip › Appendix Figure S1/Appendix Figure S1F.pdf]

**Coomassie**

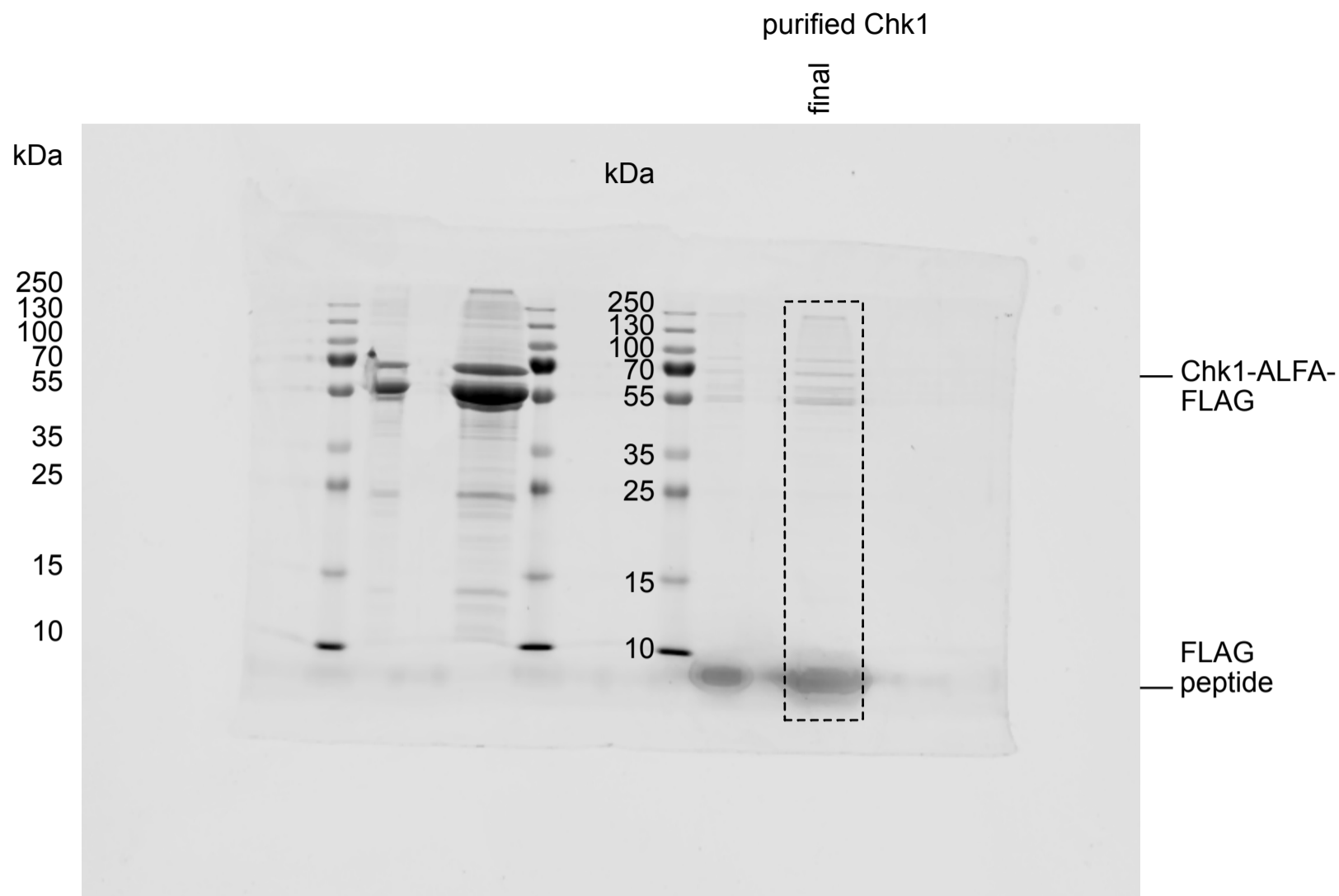

**Western Blot**

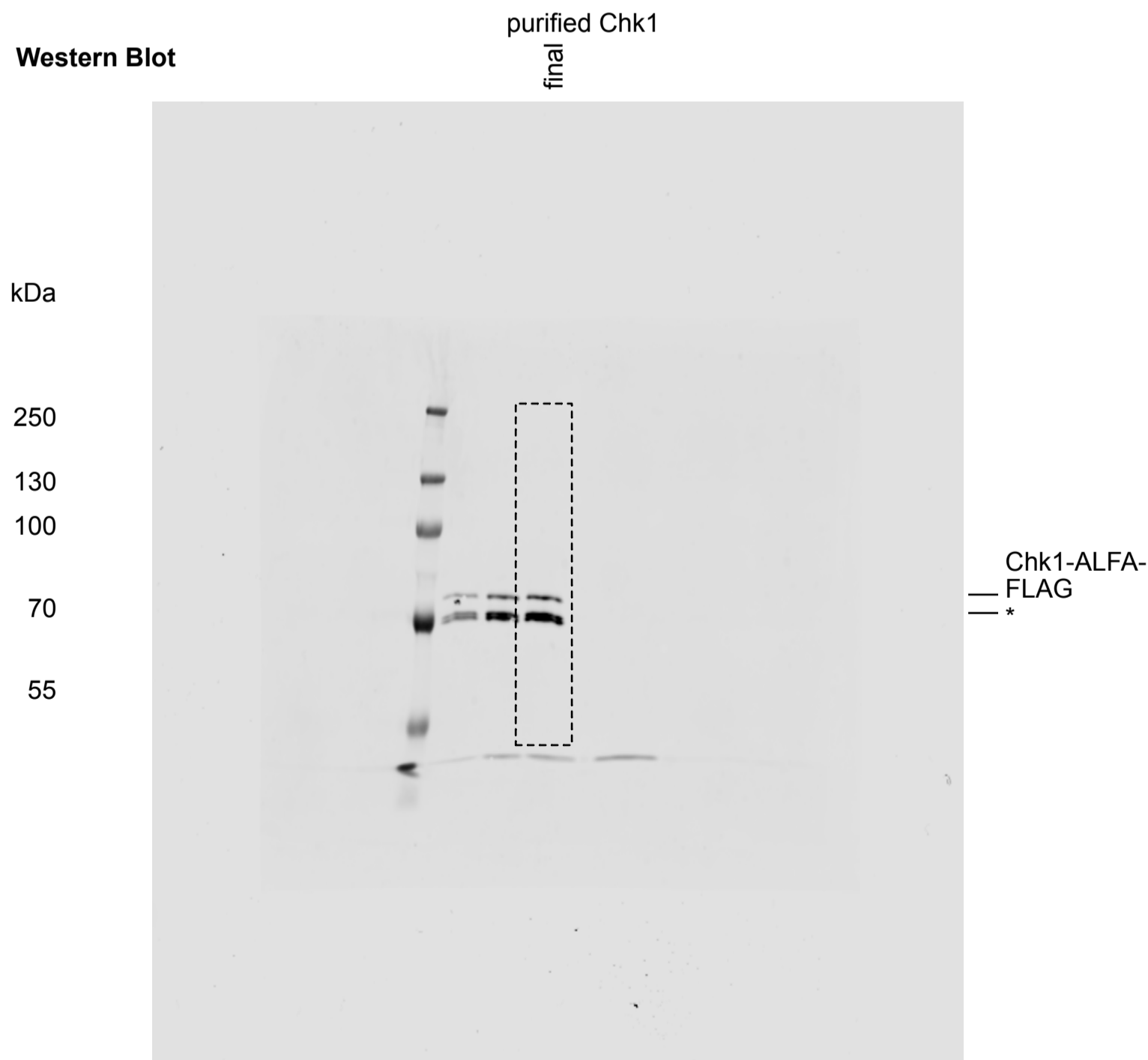

The boxed areas were used for the final figure.

Supplement: Supplementary file 16 — Appendix Figure S1 Source Data [file 44318_2025_375_MOESM16_ESM.zip › Appendix Figure S1/Appendix Figure S1B.pdf]

**purified Mgt1**

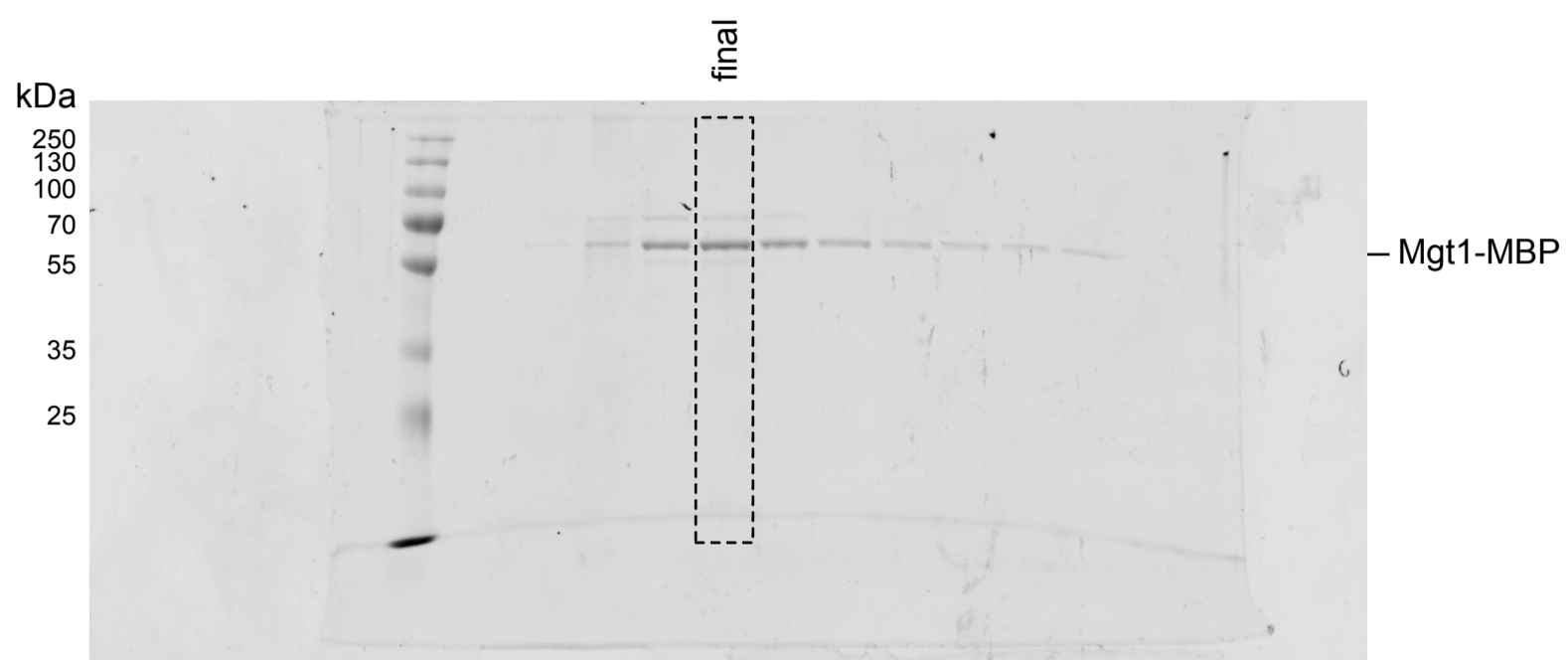

The boxed area was used for the final figure.

Supplement: Supplementary file 16 — Appendix Figure S1 Source Data [file 44318_2025_375_MOESM16_ESM.zip › Appendix Figure S1/Appendix Figure S1J.pdf]
