# Supplementary material for: Reprograming of the ubiquitin ligase Ubr1 by intrinsically disordered Roq1 through cooperating multifunctional motifs
Source: EMBO J. 2025 Feb 7;44(6):1774–803. doi: 10.1038/s44318-025-00375-7 (PMC11914429; doi:10.1038/s44318-025-00375-7)
Supplement: Supplementary file 17 — Expanded View Figures [file 44318_2025_375_MOESM17_ESM.pdf]

Expanded View Figures

Figure EV1. In vitro reconstitution of Ubr1 regulation by Roq1.

(**A**) Western blot of HA tag from ubiquitination assays with Roq1(22-104)-HA and Roq1(22-60)-HA in the absence of a designated Ubr1 substrate. Ubiquitination reactions were stopped after 30 min with buffer containing dithiothreitol and, where indicated, treated with NaOH to hydrolyze ester bonds between Roq1 and ubiquitin. Ubr1 catalyzes the formation of NaOH-sensitive conjugates of Roq1(22-104) and ubiquitin, which is almost completely lost by shortening Roq1 to Roq1(22-60). Note that the bands of unmodified Roq1(22-60) are weaker than those of unmodified Roq1(22-104) even though equal molar amounts of the two proteins were used for the assays. The reason for this difference is that the small Roq1(22-60) is only weakly bound by the nitrocellulose membrane used for western blotting so that its amounts are lower than they should be. Less ubiquitinated Roq1 is generated from Roq1(22-60) than from Roq1(22-104). WT, wild-type. (**B**) Cellular levels of the SHRED reporter Rtn1Pho8*-GFP after tunicamycin treatment for 5 h relative to levels in untreated cells, as measured by flow cytometry. The *roq1* mutant cells contained an empty plasmid (no Roq1), a plasmid encoding wild-type Roq1 (WT Roq1), ubiquitin-Roq1(22-104) or ubiquitin-Roq1(22-60). The ubiquitin fusions are processed by cells to yield Roq1(22-104) or Roq1(22-60) starting with R22. Bars are the mean ± s.e.m.; $n = 3$ biological replicates. (**C**) Western blot of Pho8 from solubility assays of Pho8*. Ubiquitination assays including Roq1(22-60) were carried out for 0 or 90 min and soluble and insoluble Pho8* were separated by centrifugation. T = total; S = supernatant; P = pellet. (**D**) Western blot of Pho8 from Pho8* ubiquitination assays with and without Ubr1 and Roq1(22-60). No Pho8* ubiquitination occurs in the absence of Ubr1. (**E**). Western blot of Pho8 from Pho8* ubiquitination assays with and without Roq1(22-60). Ubiquitination reactions were stopped after 90 min and, where indicated, treated with NaOH to hydrolyze ester bonds between Pho8* and ubiquitin. Ubiquitin-Pho8* conjugates were resistant to alkaline hydrolysis, showing that they consisted of amide rather than oxyester bonds. (**F**) Western blot of Pho8 from Pho8* ubiquitination assays with different concentrations of Roq1(22-60). Roq1(22-60) was omitted or used at molar ratios of 1:1, 2:1, 5:1 and 10:1 relative to Ubr1. (**G**) Western blot of ALFA tag from untreated and tunicamycin-treated control cells (strain SSY122) that do not express an ALFA-tagged protein or cells with chromosomally ALFA-tagged Ubr1 and Roq1 (strain SSY4598). The levels of Ubr1 and Roq1 are similar in cells exposed to tunicamycin-induced proteotoxic stress. Note that proteolytic cleavage of ALFA-tagged Roq1 is inefficient, likely because of the position of the tag at the extreme C-terminus (Szoradi et al, 2018). Asterisks mark non-specific bands. n.t., no treatment; Tm, tunicamycin. (**H**) Western blot of Pho8 from Pho8* ubiquitination assays without and with Roq1(22-104) or Roq1(22-60) for the times indicated.Source data are available online for this figure.

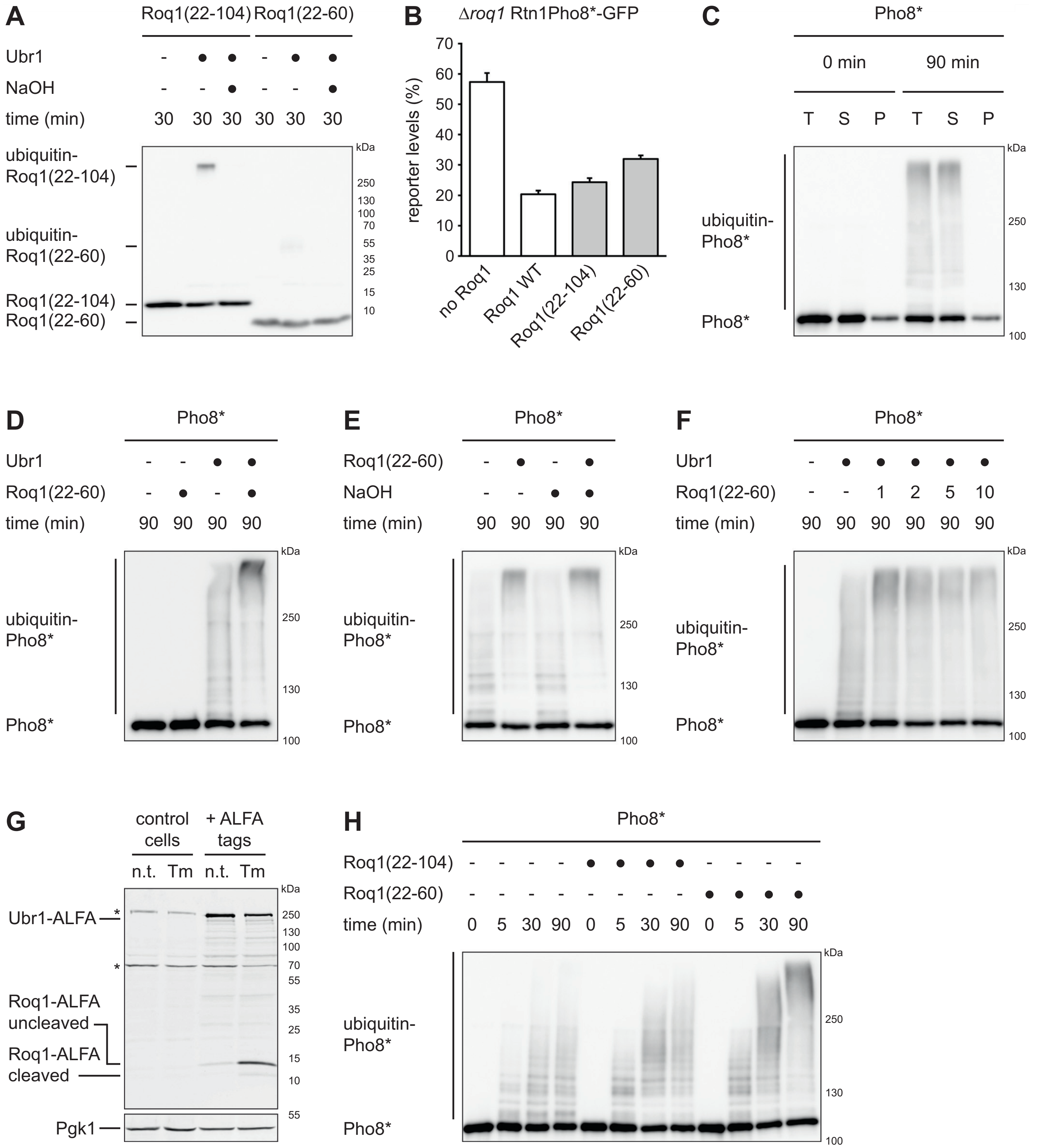
A
Roq1(22-104) Roq1(22-60)
Ubr1
NaOH
time (min)
30 30 30 30 30 30
ubiquitin-Roq1(22-104)
ubiquitin-Roq1(22-60)
Roq1(22-104)
Roq1(22-60)
kDa
250 130 100 70 55 35 25 15 10
B
Δroq1 Rtn1Pho8*-GFP
reporter levels (%)
0 10 20 30 40 50 60 70
no Roq1
Roq1 WT
Roq1(22-104)
Roq1(22-60)
C
Pho8*
0 min
90 min
T S P T S P
ubiquitin-Pho8*
Pho8*
kDa
250 130 100
D
Pho8*
Ubr1
Roq1(22-60)
time (min)
90 90 90 90
ubiquitin-Pho8*
Pho8*
kDa
250 130 100
E
Pho8*
Roq1(22-60)
NaOH
time (min)
90 90 90 90
ubiquitin-Pho8*
Pho8*
kDa
250 130 100
F
Pho8*
Ubr1
Roq1(22-60)
- - 1 2 5 10
time (min)
90 90 90 90 90 90
ubiquitin-Pho8*
Pho8*
kDa
250 130 100
G
control cells
+ ALFA tags
n.t. Tm n.t. Tm
Ubr1-ALFA
Roq1-ALFA uncleaved
Roq1-ALFA cleaved
Pgk1
kDa
250 130 100 70 55 35 25 15 10 55
H
Pho8*
Roq1(22-104)
Roq1(22-60)
time (min)
0 5 30 90 0 5 30 90 0 5 30 90
ubiquitin-Pho8*
Pho8*
kDa
250 130 100

 © The Author(s)

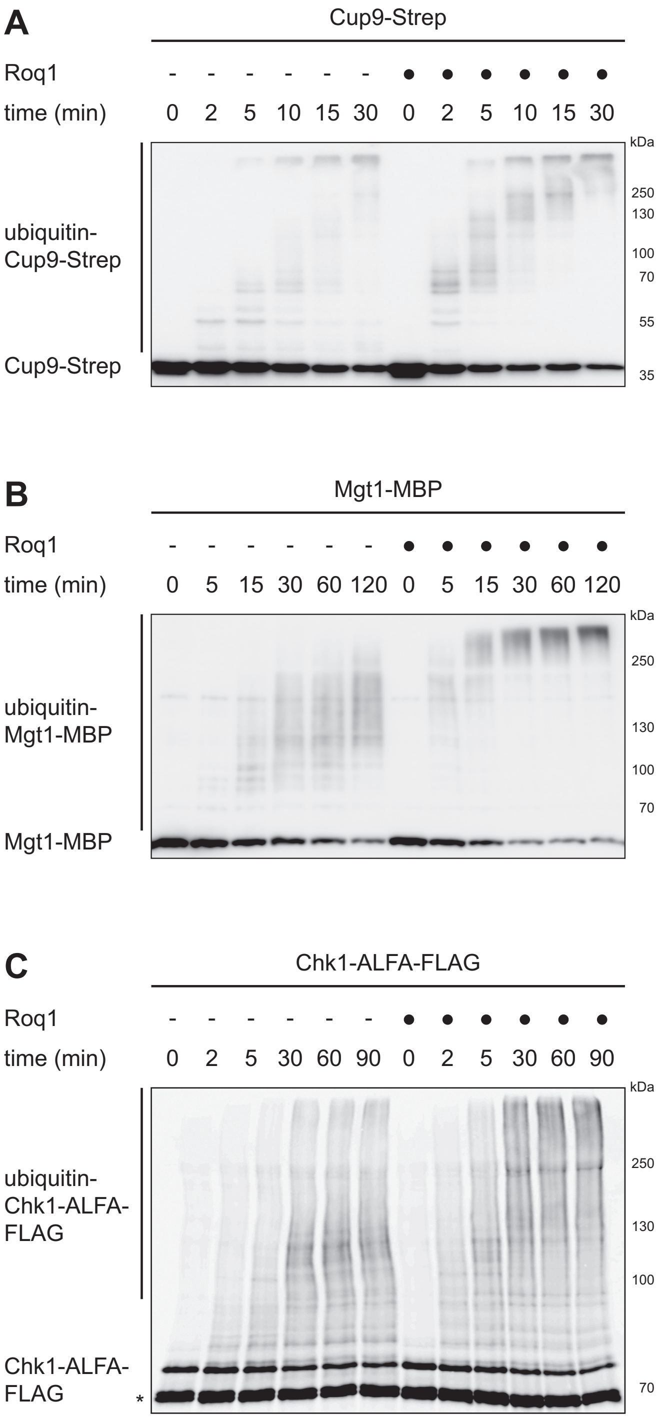

Figure EV2. Roq1 stimulates ubiquitination of folded Ubr1 substrate proteins with internal degrons.

(**A**) Western blot of Strep tag from Cup9-Strep ubiquitination assays without and with Roq1(22-60) for the times indicated. (**B**) Western blot of maltose-binding protein (MBP) tag from Mgt1-MBP ubiquitination assays without and with Roq1(22-60) for the times indicated. (**C**) Western blot of FLAG tag from Chk1-ALFA-FLAG ubiquitination assays without and with Roq1(22-60) for the times indicated. The asterisk denotes truncated Chk1 that arose during the expression and purification of Chk1-ALFA-FLAG.Source data are available online for this figure.

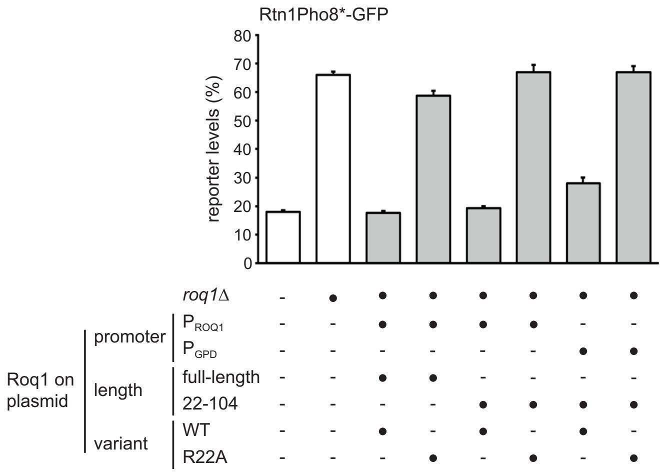

Figure EV3. Overexpressed Roq1(R22A) does not activate Ubr1.

Cellular levels of the SHRED reporter Rtn1Pho8*-GFP after tunicamycin treatment for 5 h relative to levels in untreated cells, as measured by flow cytometry. Cells were wild-type or lacked chromosomal *ROQ1*. The *roq1* mutants contained plasmids encoding Roq1 variants that were expressed under the endogenous *ROQ1* promoter (P_{ROQ1}) or the strong *GPD* promoter (P_{GPD}), were full-length or lacked the first 21 residues (22-104), and were otherwise wild-type or contained the R22A mutation. Roq1 with the R22A mutation did not support SHRED, even when overexpressed. Full-length Roq1 and ubiquitin-fused Roq1(22-104) are processed in cells by Ynm3 or ubiquitin proteases to yield Roq1(22-104). Ubiquitin-fused Roq1(22-104) was used when Roq1 was expressed under the *GPD* promoter to avoid that cleavage of Roq1 by Ynm3 becomes limiting for the amounts of Roq1(22-104) available for Ubr1 activation. Bars are the mean ± s.e.m.; $n = 3$ biological replicates.Source data are available online for this figure.

 © The Author(s)

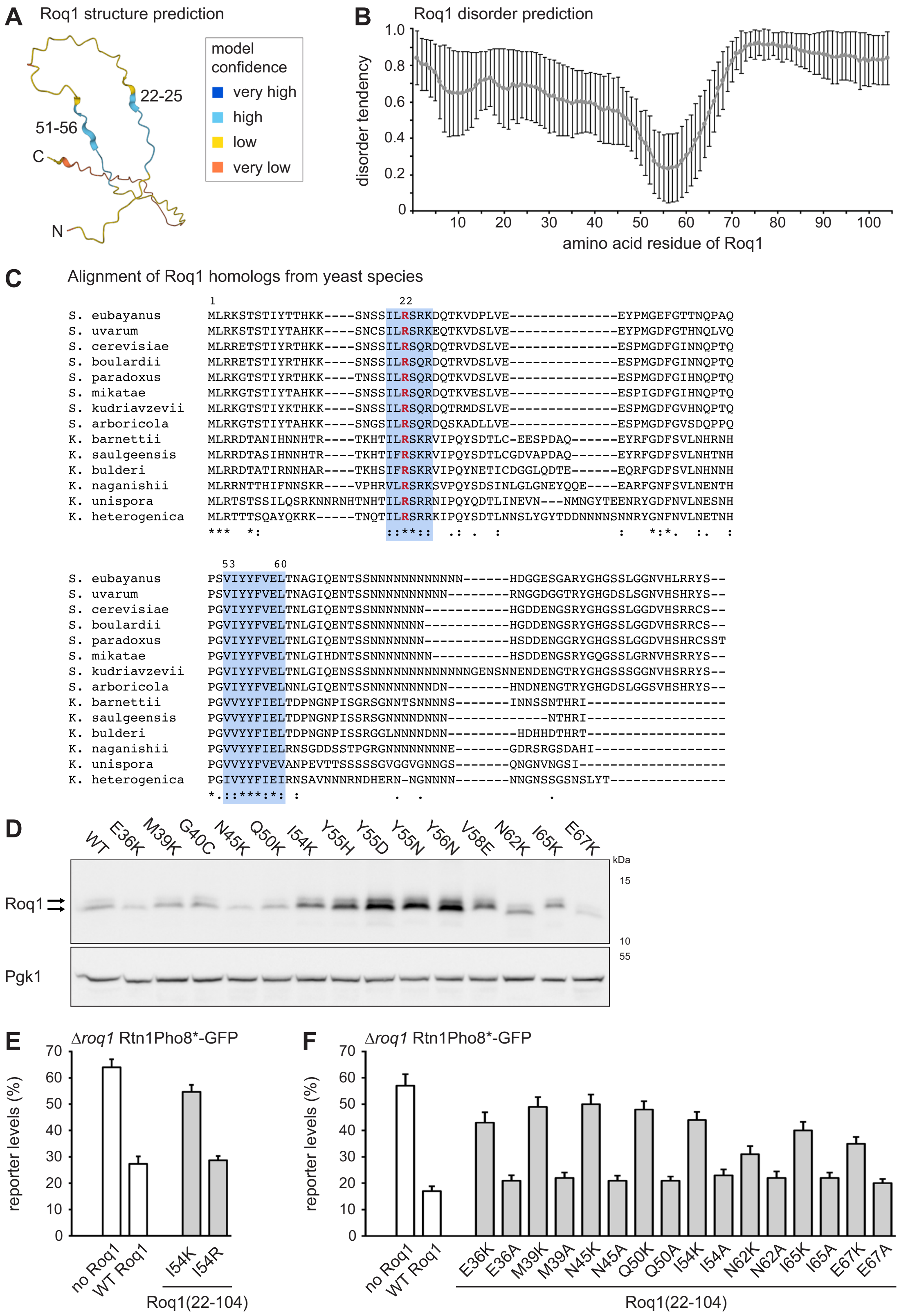
A
Roq1 structure prediction
model confidence
very high
high
low
very low
22-25
51-56
C
N
B
Roq1 disorder prediction
disorder tendency
amino acid residue of Roq1
C
Alignment of Roq1 homologs from yeast species
1
22
S. eubayanus MLRKSTSTIYTTHKK----SNSSILRSRKDQTKVDPLVE--------------EYPMGEFGTTNQPAQ
S. uvarum MLRKSTSTIYTAHKK----SNCSILRSRKEQTKVDSLVE--------------EYPMGDFGTHNQLVQ
S. cerevisiae MLRRETSTIYRTHKK----SNSSILRSQRDQTRVDSLVE--------------ESPMGDFGINNQPTQ
S. boulardii MLRRETSTIYRTHKK----SNSSILRSQRDQTRVDSLVE--------------ESPMGDFGINNQPTQ
S. paradoxus MLRKGTSTIYRTHKK----TNSSILRSQRDQTKVDSLVE--------------ESPMGDFGIHNQPTQ
S. mikatae MLRKGTSTIYTAHKK----SNSSILRSQRDQTKVESLVE--------------ESPIGDFGIHNQPTQ
S. kudriavzevii MLRKGTSTIYKTHKK----SNSSILRSQRDQTRMDSLVE--------------ESPMGDFGVHNQPTQ
S. arboricola MLRKGTSTIYTAHKK----SNGSILRSQRDQSKADLLVE--------------ESPMGDFGVSDQPPQ
K. barnettii MLRRDTANIHNNHTR----TKHTILRSKRVIPQYSDTLC-EESPDAQ------EYRFGDFSVLNHRNH
K. saulgeensis MLRRDTASIHNNHTR----TKHTIFRSKRVIPQYSDTLCGDVAPDAQ------EYRFGDFSVLNHTNH
K. bulderi MLRRDTATIRNNHAR----TKHSIFRSKRVIPQYNETICDGGLQDTE------EQRFGDFSVLNHNNH
K. naganishii MLRRNTTHIFNNSKR----VPHRVLRSRKSVPQYSDSINLGLGNEYQQE----EARFGNFSVLNENTH
K. unispora MLRTSTSSILQSRKNNRNHTNHTILRSRRNIPQYQDTLINEVN---NMNGYTEENRYGDFNVLNESNH
K. heterogenica MLRTTTSQAYQKRK-----TNQTILRSRRKIPQYSDTLNNSLYGYTDDNNNNSNNRYGNFNVLNETNH
53
60
S. eubayanus PSVIYYFVELTNAGIQENTSSNNNNNNNNNNNN------HDGGESGARYGHGSSLGGNVHLRRYS--
S. uvarum PSVIYYFVELTNAGIQENTSSNNNNNNNNNNN--------RNGGDGGTRYGHGDSLSGNVHSHRYS--
S. cerevisiae PGVIYYFVELTNLGIQENTSSNNNNNNNN-----------HGDDENGSRYGHGSSLGGDVHSRRCS--
S. boulardii PGVIYYFVELTNLGIQENTSSNNNNNNNN-----------HGDDENGSRYGHGSSLGGDVHSRRCS--
S. paradoxus PGVIYYFVELTNLGIQENTSSNNNNNNNN-----------HSDDENGGRYGHGSSLGGDVHSHRCSST
S. mikatae PGVIYYFVELTNLGIHDNTSSNNNNNNNNN----------HSDDENGSRYGQGSSLGRNVHSRRYS--
S. kudriavzevii PGVIYYFVELTNLGIQENSSSNNNNNNNNNNNNNGENSNNENDENGTRYGHGSSSGGNVHSRRYS--
S. arboricola PGVIYYFVELNNLGIQENTSSNNNNNNNNNDN--------HNDNENGTRYGHGDSLGGSVHSHRYS--
K. barnettii PGVVYYFIELTDPNGNPISGRSGNNTSNNNNS--------INNSSNTHRI------------------
K. saulgeensis PGVVYYFIELTDPNGNPISSRSGNNNNDNNN-------------NTHRI------------------
K. bulderi PGVVYYFIELTDPNGNPISSRGGLNNNNDNN---------HDHHDTHRT------------------
K. naganishii PGVVYYFIELRNSGDDSSTPGRGNNNNNNNNE-------GDRSRGSDAHI------------------
K. unispora PGVVYYFVEVANPEVTTSSSSSGVGGVGNNGS-------QNGNVNGSI-------------------
K. heterogenica PGIVYYFIEIRNSAVNNNRNDHERN-NGNNNN-------NNGNSSGSNSLYT---------------
D
WT
E36K
M39K
G40C
N45K
Q50K
I54K
Y55H
Y55D
Y55N
Y56N
V58E
N62K
I65K
E67K
kDa
15
10
55
Roq1
Pgk1
E
Δroq1 Rtn1Pho8*-GFP
reporter levels (%)
no Roq1
WT Roq1
I54K
I54R
Roq1(22-104)
F
Δroq1 Rtn1Pho8*-GFP
reporter levels (%)
no Roq1
WT Roq1
E36K
E36A
M39K
M39A
N45K
N45A
Q50K
Q50A
I54K
I54A
N62K
N62A
I65K
I65A
E67K
E67A
Roq1(22-104)

Figure EV4. Roq1 contains a functionally essential hydrophobic motif.

(**A**) Roq1 structure prediction by AlphaFold. (**B**) Roq1 disorder prediction. The plot shows the average disorder tendency across the Roq1(1–104) sequence on a scale of 0 to 1. Data are the mean of the predictions of twelve different algorithms for disorder prediction. Error bars show the standard deviation. (**C**) Multiple sequence alignment of Roq1 homologs from fourteen yeast species. The numbering of the residues corresponds to the *S. cerevisiae* sequence. * fully conserved residue, : strongly conserved residue. weakly conserved residue. (**D**) Western blot of HA tag and Pgk1 from *roq1* mutant strains expressing ubiquitin-Roq1(22-104)-HA(74) variants under the control of the strong *GPD* promoter. Roq1(22-104) naturally runs as a double band. The nature of the slower migrating band is unknown. Pgk1 served as a loading control. WT, wild-type. (**E**, **F**) Cellular levels of the SHRED reporter Rtn1Pho8*-GFP after tunicamycin treatment for 5 h relative to the levels in untreated cells, as measured by flow cytometry. The *roq1* mutant cells contained an empty plasmid (no Roq1), a plasmid encoding wild-type Roq1 (WT Roq1) or plasmids encoding variants of ubiquitin-Roq1(22-104). Ubiquitin-Roq1 fusions are processed by cells to yield Roq1(22-104). Bars are the mean ± s.e.m.; $n = 3$ biological replicates. Source data are available online for this figure.

 © The Author(s)

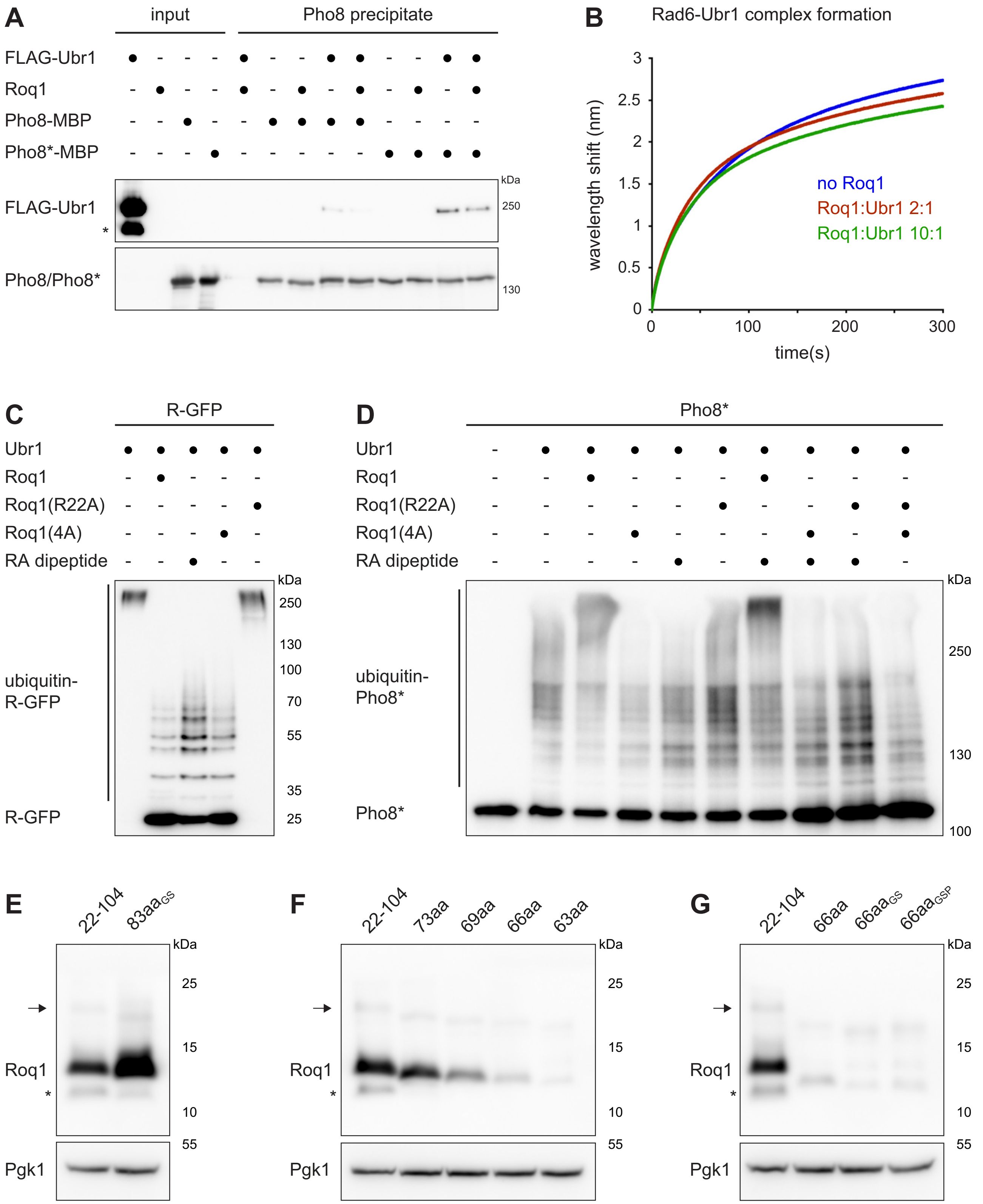
A
input
Pho8 precipitate
FLAG-Ubr1
Roq1
Pho8-MBP
Pho8*-MBP
kDa
FLAG-Ubr1
250
*
Pho8/Pho8*
130
B
Rad6-Ubr1 complex formation
wavelength shift (nm)
time(s)
no Roq1
Roq1:Ubr1 2:1
Roq1:Ubr1 10:1
C
R-GFP
Ubr1
Roq1
Roq1(R22A)
Roq1(4A)
RA dipeptide
ubiquitin-R-GFP
R-GFP
D
Pho8*
ubiquitin-Pho8*
Pho8*
E
22-104
83aaGS
Roq1
Pgk1
F
22-104
73aa
69aa
66aa
63aa
G
66aaGS
66aaGSP

Figure EV5. Effect of Roq1 on Pho8* recognition and Rad6 recruitment, effect of separating R22 and the hydrophobic motif different molecules, and expression levels of Roq1 linker variants.

(**A**) Western blots of FLAG tag and Pho8 from input and Pho8 precipitate of an in vitro pulldown assays with FLAG-Ubr1, Pho8/Pho8* fused to maltose-binding protein (MBP), and Roq1(22-60)-HA as indicated. Pho8 or Pho8*-MBP were precipitated with amylose resin. The asterisk denotes truncated Ubr1 that arose during the expression and purification of FLAG-Ubr1. (**B**) Biolayer interferometry of Rad6-Ubr1 complex formation with immobilized Rad6 and soluble Ubr1. Complex formation was tested in the absence of Roq1(22-104) and with Roq1(22-104):Ubr1 molar ratios of 2:1 or 10:1. Data are the mean of two independent experiments. (**C**) Western blot of GFP from R-GFP ubiquitination assays with Roq1(22-60), Roq1(22-60)(R22A), Roq1(22-60)(4A) or an RA dipeptide. Wild-type Roq1, the RA dipeptide and Roq1(4A) are able to bind the Ubr1 type-1 site and suppress ubiquitination of R-GFP, whereas Roq1(R22A) is not. 4A = Y55A,Y56A,F57A,V58A. (**D**) Western blot of Pho8 from Pho8* ubiquitination assays with combinations of Roq1(22-60), Roq1(22-60)(R22A), Roq1(22-60)(4A) and an RA dipeptide. Combining a type-1 site binder, Roq1(4A) or the RA dipeptide, with the hydrophobic motif-containing Roq1(R22A) does not reconstitute Roq1 activity. 4A = Y55A,Y56A,F57A,V58A. (**E**) Western blot of HA tag and Pgk1 from cells expressing Roq1(22-104)-HA(74) or Roq1($83aa_{GSP}$)-HA(74), in which the sequence between S23 and P51 of Roq1 was replaced with a GSP-based linker. The arrow indicates unprocessed ubiquitin-Roq1, in which the N-terminal ubiquitin has not been removed by ubiquitin proteases. The asterisk marks truncated Roq1. (**F**) Western blot of HA tag and Pgk1 from cells expressing Roq1(22-104)-HA(74) or Roq1 variants in which the sequence between S23 and P51 was successively shortened. The arrow indicates unprocessed ubiquitin-Roq1, in which the N-terminal ubiquitin has not been removed by ubiquitin proteases. The asterisks marks truncated Roq1. (**G**) Western blot of HA tag and Pgk1 from cells expressing Roq1(22-104)-HA(74), Roq1(63aa)-HA(74) with a shortened sequence between S23 and P51, Roq1($63aa_{GS}$)-HA(74) with a GS-based linker replacing the shortened sequence or Roq1($63aa_{GSP}$)-HA(74) with a GSP-based linker replacing the shortened sequence. The arrow indicates unprocessed ubiquitin-Roq1, in which the N-terminal ubiquitin has not been removed by ubiquitin proteases. The asterisk marks truncated Roq1. Source data are available online for this figure.

 © The Author(s)
