# Supplementary material for: Reprograming of the ubiquitin ligase Ubr1 by intrinsically disordered Roq1 through cooperating multifunctional motifs
Source: EMBO J. 2025 Feb 7;44(6):1774–803. doi: 10.1038/s44318-025-00375-7 (PMC11914429; doi:10.1038/s44318-025-00375-7)
Supplement: Supplementary file 8 — Source data Fig. 6 [file Figure 6A.pdf › Figure 6/Figure 6A.pdf]

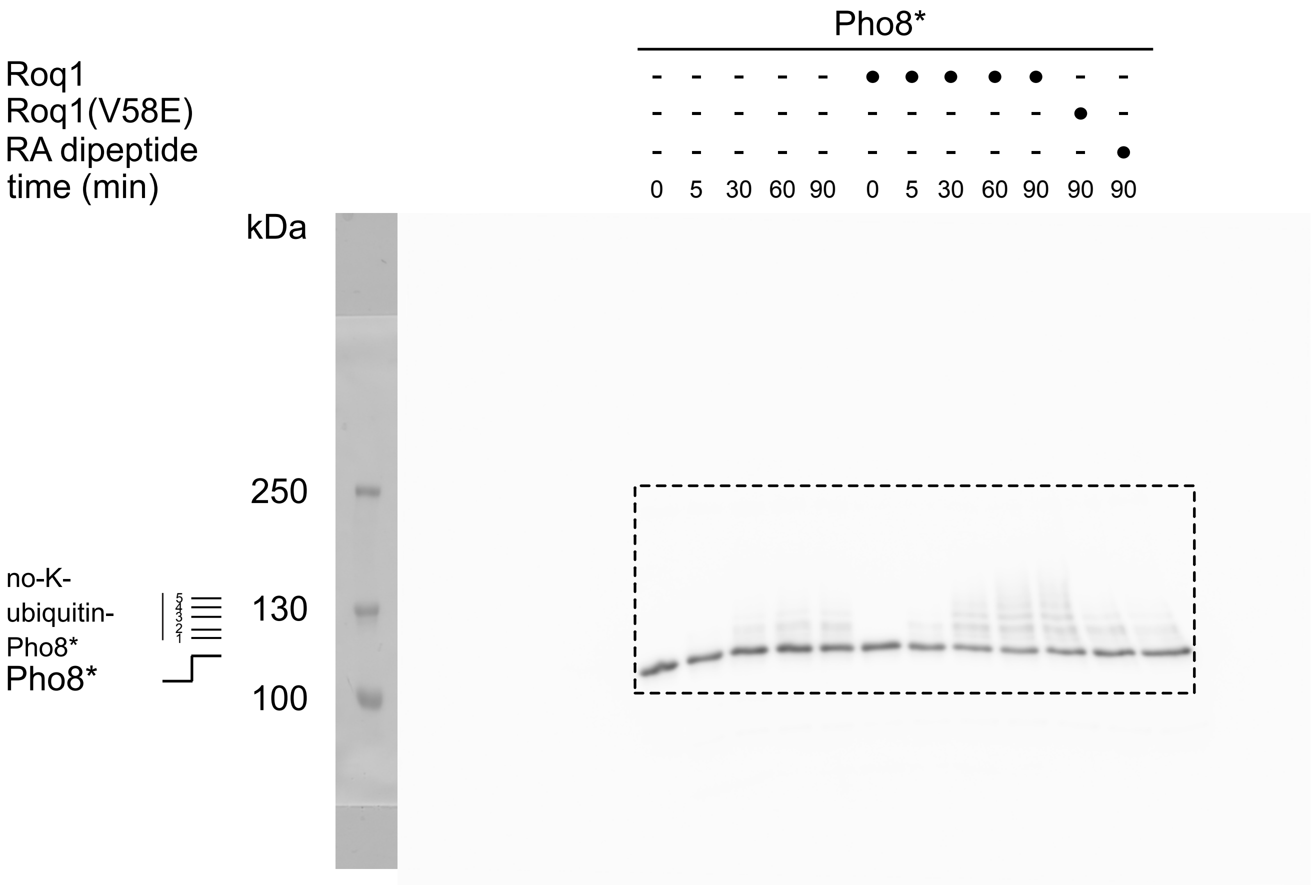

The boxed area was used for the final figure.
